# MALDI-TOF Mass Spectrometry Discriminates Known Species and Marine Environmental Isolates of *Pseudoalteromonas*

**DOI:** 10.3389/fmicb.2016.00104

**Published:** 2016-02-12

**Authors:** Kaveh Emami, Andrew Nelson, Ethan Hack, Jinwei Zhang, David H. Green, Gary S. Caldwell, Ehsan Mesbahi

**Affiliations:** ^1^Centre for Bacterial Cell Biology, Institute for Cell and Molecular Biosciences, Newcastle UniversityNewcastle upon Tyne, UK; ^2^Faculty of Health and Life Sciences, Northumbria UniversityNewcastle upon Tyne, UK; ^3^School of Biology, Newcastle UniversityNewcastle upon Tyne, UK; ^4^Medical Research Council Protein Phosphorylation and Ubiquitylation Unit, College of Life Sciences, University of DundeeDundee, UK; ^5^Microbial and Molecular Biology, Scottish Association for Marine Science, Scottish Marine InstituteOban, UK; ^6^School of Marine Science and Technology, Newcastle UniversityNewcastle upon Tyne, UK; ^7^Faculty of Science, Agriculture and Engineering, Newcastle UniversityNewcastle upon Tyne, UK

**Keywords:** *Pseudoalteromonas*, MALDI-TOF, mass spectrometry, North Sea, biotyping, marine bacteria, ribosomal RNA genes

## Abstract

The genus *Pseudoalteromonas* constitutes an ecologically significant group of marine *Gammaproteobacteria* with potential biotechnological value as producers of bioactive compounds and of enzymes. Understanding their roles in the environment and bioprospecting for novel products depend on efficient ways of identifying environmental isolates. Matrix Assisted Laser Desorption/Ionization-Time of Flight Mass Spectrometry (MALDI-TOF MS) biotyping has promise as a rapid and reliable method of identifying and distinguishing between different types of bacteria, but has had relatively limited application to marine bacteria and has not been applied systematically to *Pseudoalteromonas*. Therefore, we constructed a MALDI-TOF MS database of 31 known *Pseudoalteromonas* species, to which new isolates can be compared by MALDI-TOF biotyping. The ability of MALDI-TOF MS to distinguish between species was scrutinized by comparison with 16S rRNA gene sequencing. The patterns of similarity given by the two approaches were broadly but not completely consistent. In general, the resolution of MALDI-TOF MS was greater than that of 16S rRNA gene sequencing. The database was tested with 13 environmental *Pseudoalteromonas* isolates from UK waters. All of the test strains could be identified to genus level by MALDI-TOF MS biotyping, but most could not be definitely identified to species level. We conclude that several of these isolates, and possibly most, represent new species. Thus, further taxonomic investigation of *Pseudoalteromonas* is needed before MALDI-TOF MS biotyping can be used reliably for species identification. It is, however, a powerful tool for characterizing and distinguishing among environmental isolates and can make an important contribution to taxonomic studies.

## Introduction

Members of the bacterial genus *Pseudoalteromonas* belong to a large and cosmopolitan group of marine bacteria (Ivanova et al., 2004a, 2014; Radjasa et al., 2007; Skovhus et al., 2007; Bian et al., 2012), many of which are of ecological and biotechnological interest. A key ecological feature of this genus is their diverse array of biotic associations with marine eukaryotes (Holmström and Kjelleberg, 1999). The genus includes species that produce growth promoters for macroalgae (Dimitrieva et al., 2006), induce larval settlement and metamorphosis in both vertebrates and invertebrates (Huang et al., 2007; Wesseling et al., 2015), mediate microalgae bloom termination events (Mayali and Azam, 2004; Kim et al., 2009), and are pathogens of a range of organisms—many of which are of economic significance (Sawabe et al., 1998; Costa-Ramos and Rowley, 2004; Pujalte et al., 2007; Choudhury et al., 2015). Further to their ecological importance, pseudoalteromonads are known to produce a range of biotechnologically valuable enzymes, some of which are functional at low temperatures (Margesin and Schinner, 1994; Ivanova et al., 2003). Additional industrial interest resides in their potential to synthesize antibiotics (Bowman, 2007), prevent biological fouling of maritime structures and vessels (Holmström et al., 2002), and in defining their role in food spoilage (Broekaert et al., 2013).

The first description of a pseudoalteromonad, a fish pathogen, was by Bein (1954), although originally as *Flavobacterium piscicida*. Buck et al. (1963) moved this species to the genus *Pseudomonas*. On the basis of 16S rRNA gene sequence data, Gauthier et al. (1995) formally described *Pseudoalteromonas* as a distinct genus containing *P. piscicida* and several species previously assigned to the genus *Alteromonas*. Ivanova et al. (2004a) transferred this genus and its closest relative *Algicola* to the family *Pseudoalteromonadaceae*. For more information on the currently-recognized species of *Pseudoalteromonas*, see http://www.bacterio.net/pseudoalteromonas.html. While 16S rRNA gene sequence data are accepted for the definition of phylogenetic relationships between bacterial species, they sometimes lack the specificity required to distinguish between close relatives (Tindall et al., 2010). This is true in the case of the many species of *Pseudoalteromonas* that display 16S rRNA gene sequence identities as high as 98–99.9% (Ivanova et al., 2004a, 2014). To circumvent this lack of phylogenetic resolution, other molecular tools such as *gyrB* gene sequences have been demonstrated to be useful for discrimination of many different bacteria, including *Pseudoalteromonas* (Venkateswaran and Dohmoto, 2000). Nevertheless, alternative methods that discriminate between phenotypically different species are required.

The discovery and identification of new marine bacterial isolates by conventional molecular methods such as 16S rRNA gene sequencing remains relatively expensive and time consuming, despite widening access to next generation sequencing platforms and allied bioinformatics tools. As sequencing technologies advance it seems inevitable that species identification by whole genome sequencing (or some form thereof) will become the mainstay of bacteriology. However, there remains a need for rapid, reliable, affordable and high resolution approaches for bacterial identification. One such approach is mass spectrometry–based chemotaxonomy utilizing matrix-assisted laser desorption ionization-time of flight mass spectrometry (MALDI-TOF MS). When used correctly, MALDI-TOF MS efficiently provides reliable mass spectra, which are dominated by high-abundance proteins, especially ribosomal proteins (Schumann and Maier, 2014). The method typically shows high reproducibility among different laboratories provided that consistent conditions and instrumentation are used (Schumann and Maier, 2014; Singhal et al., 2015). Because of its efficiency and reliability, over the past decade MALDI-TOF MS has become an established taxonomic and diagnostic tool for identification and sub-typing of bacteria in clinical and environmental contexts (for example Ruelle et al., 2004; Demirev and Fenselau, 2008; Schumann and Maier, 2014; Singhal et al., 2015). Examples of the application of MALDI-TOF MS to marine bacterial chemotaxonomy include studies of *Vibrio* (Dieckmann et al., 2010; Oberbeckmann et al., 2011) and *Alteromonas* (Ng et al., 2013). Ng et al. (2013), for example, found the method comparable and complementary to 16S rRNA gene sequencing for classification of *Alteromonas*. More pertinently to the current study, Dieckmann et al. (2005) used MALDI-TOF MS profiles to separate species of sponge-associated *Pseudoalteromonas* and *Alteromonas*. In addition, they found species-specific biomarkers sufficient to discriminate three *Pseudoalteromona*s species. Further, several strains of *Pseudoalteromonas* isolated from ballast water have been characterized using MALDI-TOF MS (Emami et al., 2012).

For routine bacterial identification by MALDI-TOF MS, the standard approach is to compare the mass spectra of isolates with the mass spectra of type strains—this approach is termed MALDI-TOF MS biotyping. A major limiting factor in the use of MALDI-TOF MS biotyping for characterizing aquatic bacteria is the lack of suitable mass spectra databases. To begin to address this constraint we have constructed the first MALDI-TOF MS library for *Pseudoalteromonas* species. To investigate the value of this database, we tested it with 13 unknown environmental isolates: 3 from algae and 10 from seawater. In so doing, we demonstrated that the resolution of MALDI-TOF MS exceeds that of the 16S rRNA gene alone and provides a useful approach to distinguish between closely related but functionally distinct *Pseudoalteromonas* strains. The construction of a MALDI-TOF MS database for *Pseudoalteromonas* provides a valuable resource complementary to the available genomic tools for detailed characterization of *Pseudoalteromonas* isolates as part of a polyphasic approach to bacterial taxonomy.

## Materials and methods

### *Pseudoalteromonas* type strains

*Pseudoalteromonas* type strains were purchased from DSMZ (German Collection of Microorganisms and Cell Cultures GmbH, Braunschweig, Germany), or JCM (Japan collection of microorganisms). Each strain was cultured according to the supplier's instructions, then transferred to marine agar (MA, Difco™) plates and incubated at the recommended temperature. The species that were used for construction of the library (GenBank/EMBL/DDBJ accession numbers in brackets) were *P. agarivorans* DSM14585^T^ (AJ417594), *P. aliena* DSM16473^T^ (AY387858), *P. arabiensis* JCM 17292^T^(AB576636), *P. arctica* DSM18437^T^ (DQ787199), *P. atlantica* DSM6839^T^ (X82134), *P. aurantia* DSM6057^T^ (X82135), *P. carrageenovora* DSM6820^T^ (X82136), *P. citrea* DSM8771^T^ (X82137), *P. denitrificans* DSM6059^T^ (X82138), *P. espejiana* DSM9414^T^ (X82143), *P. elyakovii* JCM 21269^T^ (AB000389), *P. flavipulchra* DSM14401^T^ (AF297958), *P. haloplanktis* DSM6060^T^ (X67024), *P. issachenkonii* DSM15925^T^ (AF316144), *P. lipolytica* JCM 15903^T^ (FJ404721), *P. marina* DSM17587^T^ (AY563031), *P. mariniglutinosa* DSM15203^T^ (AJ507251), *P. nigrifaciens* DSM8810^T^ (X82146), *P. paragorgicola* DSM14403^T^ (AY040229), *P. peptidolytica* DSM14001^T^ (AB681334), *P. prydzensis* DSM14232^T^ (U85855), *P. piscicida* JCM 20779^T^ (AB090232), *P. rubra* DSM6842^T^ (X82147), *P. spongiae* JCM 12884^T^ (AY769918), *P. spiralis* DSM16099^T^ (AJ314842), *P. telluritireducens* DSM16098^T^ (AJ314843), *P. tetraodonis* DSM9166^T^ (AF214730), *P. translucida* DSM14402^T^ (AY040230), *P. tunicata* DSM14096^T^ (AAOH01000015), *P. ulvae* DSM15557^T^ (AF172987), and *P. undina* DSM6065^T^ (X82140).

### Isolation of bacteria from seawater

Water from the North Sea coastal area off the Dove Marine Laboratory, Cullercoats, UK (55°02′00″ N, 001°27′00″W) was collected in a 50,000 l tank. One liter samples were taken and passed through 2 μm filters, and then each filter was washed with 2 ml of sterile seawater. The washouts were spread on marine agar plates. Colonies with different morphologies on this medium were selected and purified through the third generation (NS isolates). Alternatively, bacteria collected from various marine environments in the Celtic Sea (water collection depths of between 5 and 35 m) and in the region of Oban (Scotland) were serially diluted tenfold in sterile seawater and subsequently spread onto ONR7a agar (Dyksterhouse et al., 1995), supplemented with *n*-hexadecane as the sole carbon source. The plates were sealed and incubated at 25°C and colonies that grew were re-streaked on *n*-hexadecane-supplemented ONR7a agar to confirm hydrocarbon utilization and to achieve colony purity (isolates DG1810, DG1838, and TG12).

### Isolation of bacteria from algae

A sterile swab was used for isolation from surface swabs from seaweeds and then to inoculate the bacteria on marine agar plates. The inoculated plates were incubated for 3 d at 15°C until colonies were observed. Isolation of bacteria from marine microalgae (*Gymnodinium catenatum, Pseudo-nitzschia multiseries, Pseudo-nitzschia seriata*) involved serial tenfold dilution of algal cultures in sterile seawater and growth on marine agar at 21–25°C. Colonies (isolates DG1135, DG1152, and DG1425) were treated as described in the above section.

### Preparation of genomic dna and PCR cloning of 16S rRNA gene

Genomic DNA extraction was carried out using the UltraClean® Microbial DNA isolation kit (MoBio) according to the manufacturer's instructions. PCR reactions were conducted using the 27f (5′-AGAGTTTGATYMTGGCTCAG-3′) and 1497r (5′-TACGGYTACCTTGTTACGACT-3′) primer pair (Suzuki and Giovannoni, 1996). Each reaction was in a total volume of 50 μl which contained Standard buffer (New England Biolabs), 0.5 μM each primer, 200 μM each dNTP, 15 μg BSA (Promega), 1.25 U Taq DNA polymerase (New England Biolabs), and 1 μl of genomic DNA. The cycling conditions were 95°C for 5 min followed by 30 cycles of 95°C for 30 s, 55°C for 30 s, 72°C for 2 min followed by a final extension step at 72°C for 10 min.

PCR products (~1465 bp) were cleaned up using the GenElute PCR clean up kit (Sigma-Aldrich) according to the manufacturer's instructions. Bidirectional DNA sequencing was performed by Geneius Laboratories Ltd (Cramlington, UK). Chromatograms were checked using Chromas Lite (v2.1.1) and consensus sequences were constructed using CAP3 (Huang and Madan, 1999). The sequences were deposited in Genbank® under the accession numbers listed in Table 1. Alternatively, bacterial 16S rRNA gene amplicon sequencing and analysis followed methods described by Green et al. (2004).

**Table 1:** ***Pseudoalteromonas* environmental isolates, their origins and their resemblance to type strains of known species according to 16S rRNA gene sequence and MALDI-TOF mass spectrum**.

**Isolate**	**Origin**	**16S rDNA Accession**	**EzTaxon**		**Biotyper**	
			**Closest species**	**Differences/Total length**	**Closest species**	**Score**
DG1135	*Gymnodinium catenatum* (dinoflagellate) algal culture	AY258115	*P. phenolica*^a^	10/1382	*P. translucida*	2.19
DG1152	*Pseudo-nitzschia multiseries* (diatom) algal culture	AY548764	*P. marina*	2/1371	*P. marina*	2.02
DG1425	*Pseudo-nitzschia seriata* (diatom) algal culture	KF482376	*P. marina*	2/1319	*P. carrageenovora*	2.01
DG1628	Scottish seawater oil-enrichment	EU239912	*P. carrageenovora*	0/1383	*P. carrageenovora*	**2.41**
DG1664	Scottish seawater oil-enrichment	EU239890	*P. elyakovii*	0/1385	*P. elyakovii*	1.97
DG1810	Celtic Sea oil-enrichment	KF482377	*P. tetraodonis*	0/1347	*P. haloplanktis*	**2.31**
DG1838	Celtic Sea oil-enrichment	KF482378	*P. elyakovii*	6/1377	*P. haloplanktis*	2.19
TG12	Scottish seawater oil-enrichment	EF685033	*P. arctica* or *P. translucida*	9/1438 or 9/1437	*P. elyakovii*	2.00
NS-07	North Sea	AB607136	*P. prydzensis*	14/1359	*P. mariniglutinosa*	1.88
NS-10	North Sea	AB607137	*P. tetraodonis* or *P. issachenkonii*	2/1396 or 2/1395	*P. haloplanktis*	2.25
NS-20	North Sea	AB607145	*P. undina*	2/1377	*P. haloplanktis*	2.27
NS-34	North Sea	AB607157	*P. undina*	4/1412	*P. haloplanktis*	**2.38**
NS-36	North Sea	AB607158	*P. prydzensis*	19/1449	*P. prydzensis*	2.08

*The EZTaxon database was queried with the sequences identified by their accession numbers. Biotyper scores are for comparisons to the MALDI-TOF MS library developed in house. Scores ≥2.30, indicating “confident” species identification according to Bruker Daltonics (MALDI Biotyper 3.1 User Manual, Bruker Daltonics, 2012), are given in bold*.

a *Not included in this study*.

### 16S rRNA gene analysis

The 16S rRNA gene sequences of the environmental isolates were checked for similarities to sequences in the EZTaxon database of bacterial type strain sequences (http://www.ezbiocloud.net/eztaxon; Kim et al., 2012). The sequences of the *Pseudoalteromonas* type strains and environmental isolates analyzed by MALDI-TOF MS, together with outgroup species (*Vibrio* and *Alteromonas*), were aligned with the Silva Incremental Sequence Aligner (http://www.arb-silva.de/aligner/; Pruesse et al., 2012) and the alignment was manually inspected and edited as required using SeaView version 4.5.2 (Gouy et al., 2010). Phylogenetic analysis was performed by Bayesian inference using MrBayes 3.2.5 (Ronquist et al., 2012). To ensure consistent treatment of all sequences, positions with missing data at the beginning and end of the alignment were removed. The program was run with the GTR (general time reversible) model, a gamma distribution of rates and a proportion of sites that were allowed to be invariable. Four simultaneous Monte Carlo chains were used and because convergence was slow, the program was run for 10,000,000 generations. The current tree was saved every 100 generations giving 100,001 trees. A 50% majority-rule consensus tree was created with a burn-in fraction of 25% (burn-in fractions of 1 and 50% and three independent runs gave the same consensus tree and almost identical clade credibility values).

### Sample preparation for MALDI-TOF MS analysis

All reagents used for MALDI-TOF MS sample extraction and analyses were of analytical reagent grade. The method for MALDI-TOF MS analysis of bacterial cell lysates has been described (Emami et al., 2012). Briefly, for each isolate, five single colonies of actively growing cultures were used to prepare the lysates. The material was thoroughly suspended in 300 μl of double-distilled water, then 900 μl of ethanol (HPLC grade; 99.9%) was added, and the components were mixed thoroughly. The samples in ethanol-water were centrifuged and the supernatant removed completely under vacuum. For extraction, 50 μl of formic acid (70% in water) was added to the bacterial pellet, the components were mixed thoroughly, and then 50 μl of acetonitrile was added and mixed thoroughly again. After centrifugation at 13000 *g* for 2 min, the supernatant was transferred to a fresh centrifuge tube, and then 1 μl of the supernatant containing the bacterial extract was transferred to a sample position on a ground steel MALDI target plate (Bruker Daltonics) and was allowed to dry at room temperature. Subsequently, the samples were each overlaid with 1 μl of MALDI matrix (see below) and air-dried again. For analysis of intact cells, a 200 μl pipette tip was used to pick up a small colony that had been freshly grown on marine agar at 25°C. The cells were transferred to the MALDI target plate, followed by addition of 1 μl of freshly prepared saturated solution of α-cyano-4-hydroxy-cinnamic acid (HCCA; see below). The sample/matrix mixture on the plate was air dried before analysis by MALDI-TOF MS.

### Preparation of HCCA matrix for MALDI-TOF MS analysis

A saturated solution of HCCA was prepared by adding 1 ml of 50% acetonitrile: 2.5% trifluoro-acetic acid (TFA) to 10 mg of HCCA (Bruker Daltonics). The mixture was subjected to sonication in a water-bath for 15 min at room temperature then centrifuged at 13000 *g* for 2 min. The supernatant was used for ionization of the proteins.

### MALDI-TOF MS parameters

For database construction and sample identification, measurements were performed in AutoExecute mode using an UltraFlex II MALDI TOF/TOF mass spectrometer (Bruker Daltonics GmbH, Leipzig, Germany) with ion source 1: 25 kV; ion source 2: 23.50 kV; and a 50.0 Hz nitrogen laser. Spectra were recorded in the positive linear mode for the relative molecular mass to charge ratio (*m/z*) range of 2000–20,000. Each spectrum was obtained by averaging 600 laser shots. The spectra were externally calibrated by using a Bacterial Test Standard that covers a mass range of 3600–17,000 Da (Bruker Daltonics).

### Mass fingerprint database

The reference database (main spectra) for type strains was constructed by using the data obtained through FlexControl software (V 3.0, Bruker Daltonics). For each isolate, 30 spectra were collected by spotting 6 replicate samples on the target plate and reading each 5 times. The resulting spectra were overlaid using the FlexAnalysis software (V 3.0, Bruker Daltonics). Outliers were deleted from the data set, and then from each overlapping set of spectra a single spectrum was selected for the analysis.

### Bacterial identification by mass spectrometry

For identification of microorganisms and mass spectra matching, the raw spectra of the unknown bacteria were imported into the Biotyper software (V 3.1, Bruker Daltonics). The analyses were performed by standard pattern matching against the main spectra of the type strains. The Biotyper software was then used to identify unknown bacterial species. The program considers parameters such as mass to charge ratio and intensity of the peaks then generates scores of 0–3. Scores of 2 and above are considered to confirm genus identification and scores of 2.30 and above considered sufficient for correct species identification (MALDI Biotyper 3.1 User Manual, Bruker Daltonics, 2012).

### Mass spectra dendrogram and heat map

Biotyper software was employed to construct a composite correlation index (CCI) distance matrix for the type strains and environmental isolates. From the distance matrix, a dendrogram was constructed by the neighbor-joining method (Saitou and Nei, 1987) using MEGA6 (Tamura et al., 2013) and a heat map was generated in Microsoft Excel (Microsoft Corporation).

### Comparison of 16S rDNA and MALDI-TOF MS trees

The likelihoods of the 16S rDNA and MALDI-TOF MS trees on the basis of the 16S rDNA sequences were calculated and compared in PAUP^*^ (Swofford, 2002) version 4.0a146 using the values for nucleotide frequency, proportion of invariant sites, nucleotide substitution rates, and shape parameter of the gamma distribution in the output from MrBayes. Likelihoods were compared using the weighted Shimodaira-Hasegawa test (Shimodaira, 2002).

## Results

**Figure 1:**
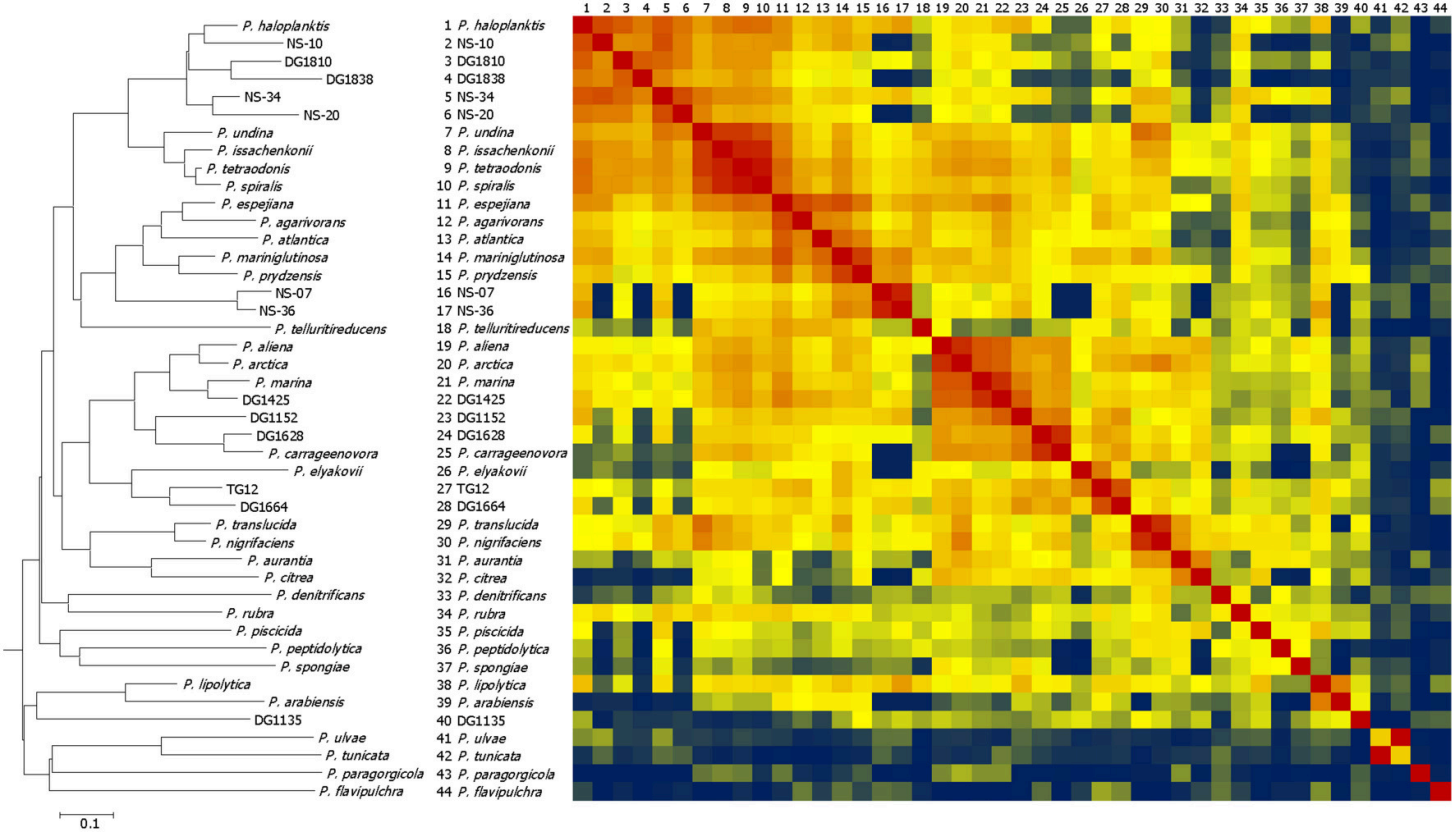
**Dendrogram and heat map comparing MALDI-TOF mass spectra of *Pseudoalteromonas* type strains and environmental isolates**. The heat map represents the distance matrix generated by using Biotyper software composite correlation index (CCI) analysis. The dendrogram was generated from the distance matrix using the neighbor joining method. In the heat map red represents the highest similarity, yellow represents the 50th percentile of similarity, and blue represents the lowest similarity. The isolates are ordered in the same way on the heat map as on the dendrogram. Numbers on the top of the heat map correspond to those on the side.

Cell lysates from 31 type strains of *Pseudoalteromonas* and 13 environmental isolates that were identified as *Pseudoalteromonas* by 16S rDNA sequencing were analyzed by MALDI-TOF MS. The mass spectra are presented in Supplementary Figure 1. Nearly all the type strains had characteristic peaks at *m/z* 4236 and *m/z* 5095 in common (Supplementary Figures 2A,B). To show the overall phenotypic grouping of the type strains and environmental isolates, a CCI distance matrix representing similarity of their high-abundance protein fingerprints was generated. Figure 1 shows a heat map representing this distance matrix in conjunction with a dendrogram grouping the isolates on the basis of their CCI distances. Most pairs of strains had composite correlation indices of 0.3 or above (shown yellow, orange, or red in the Figure 1 heat map), but some values were < 0.01 (shown deep blue in the Figure 1 heat map).

**Figure 2:**
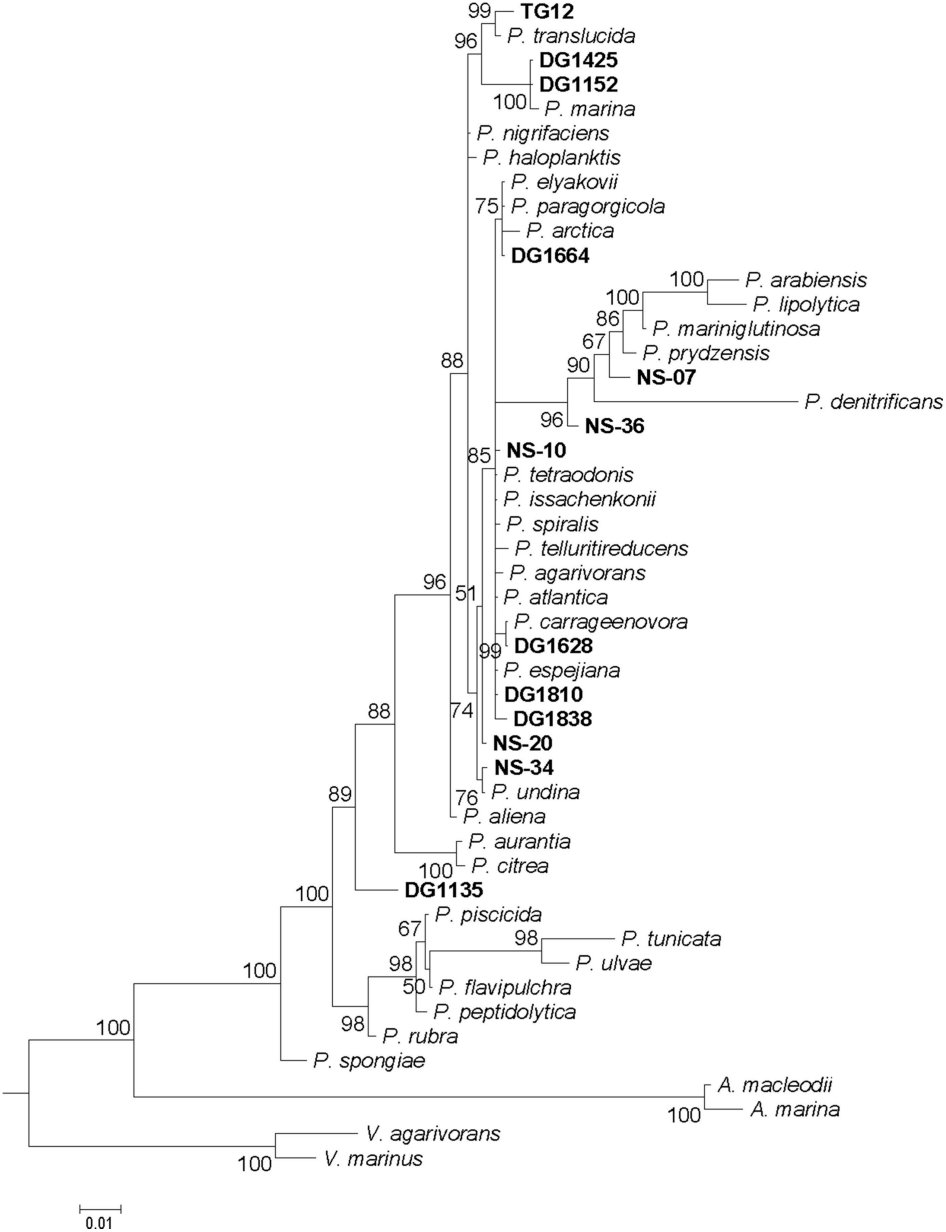
**Phylogenetic tree of 16S rRNA gene sequences of *Pseudoalteromonas* type strains and environmental isolates**. Tree is a 50% majority-rule consensus tree generated using Bayesian inference. Scale bar: 0.01 nucleotide substitutions per site. Out-grouping was performed with *Vibrio* and *Alteromonas* species. Credibility values equal to or greater than 50% are shown.

Analysis of 16S rRNA gene sequences was used to investigate how the environmental isolates (North Sea or algal associated) are related to known species and to evaluate the extent to which differences in MALDI-TOF mass spectra reflect phylogeny. A 50% majority-rule consensus phylogenetic tree was constructed by Bayesian inference (Figure 2). The clustering patterns produced by MALDI-TOF MS broadly corresponded to the 16S rDNA phylogenetic tree, with the more closely related strains generally having more similar spectra. However, the branching patterns are different: the only specific common branches between the two trees are three pairs of known species and one of a known species and an environmental isolate (see below): *P. arabiensis* and *P. lipolytica, P. citrea* and *P. aurantia, P. tunicata* and *P. ulvae*, and *P. carrageenovora* and DG1628. For the 16S rDNA sequences the 50% majority-rule consensus phylogenetic tree was a significantly better fit than the tree produced by clustering the MALDI-TOF mass spectra (*P* < 0.0001). Some mass shifts could be observed in the spectra of closely related species, possibly because of changes in amino acids (an example is in the comparison of the spectra of *P. tunicata* and *P. ulvae* below). As previously found (Ivanova et al., 2014), the *Pseudoalteromonas* phylogenetic tree includes a large group of closely-related species whose relationships were not clearly defined by 16S rDNA sequence analysis. Most of these had clearly similar but not identical MALDI-TOF mass spectra. The mass spectra of *P. undina, P. issachenkonii, P. spiralis*, and *P. tetraodonis* were particularly similar, whereas the spectrum of *P. paragorgicola* was highly distinctive (Figure 1). The group of closely-related species with relatively similar mass spectra also accommodates all the environmental isolates except DG1135. Differences between mass spectra of environmental isolates and the most similar spectra of type strains were in many cases as large as differences between each spectrum of a type strain and the most similar spectrum of another type strain.

The MALDI-TOF mass spectra of the *Pseudoalteromonas* type strains were used to construct an in-house database. The match of each type strain's mass spectrum to the spectra of the other type strains in the database, as measured by the Biotyper score (MALDI Biotyper 3.1 User Manual, Bruker Daltonics, 2012), was tested. For most type strains, the closest match to any other type strain scored < 2.30, the recommended standard threshold (MALDI Biotyper 3.1 User Manual, Bruker Daltonics, 2012) for “secure” species identifications (data not shown). The spectra of some type strains (*P. denitrificans, P. flavipulchra, P. paragorgicola, P. peptidolytica, P. piscicida, P. rubra, P. spongiae, P. tunicate*, and *P. ulvae*) were clearly distinct; they did not match the spectrum of any other type strain with a score ≥1.70, the recommended standard threshold for “probable” genus identification. In contrast, the scores for nearly all the matches among *P. issachenkonii, P. spiralis, P. tetraodonis*, and *P. undina* were at least 2.30 (the score for *P. undina* matched to *P. issachenkonii* was 2.252). The database 16S rDNA sequences for *P. issachenkonii* and *P. tetraodonis* are identical except for compensating single-base indels, but in the 16S rDNA tree (Figure 2) these species did not group specifically with *P. spiralis* and *P. undina*. Similarly, *P. agarivorans* and *P. espejiana* could not be definitively distinguished as separate species by MALDI-TOF MS biotyping: the score for *P. agarivorans* matched to *P. espejiana* was 2.293 and the score for *P. espejiana* matched to *P. agarivorans* was 2.339. The 16S rDNA sequences of these species differ by only three nucleotides. Although slightly below the threshold for species identity, the *P. aliena* and *P. arctica* spectra were very similar: the score for *P. aliena* matched to *P. arctica* was 2.241 and the score for *P. arctica* matched to *P. aliena* was 2.284.

**Figure 3:**
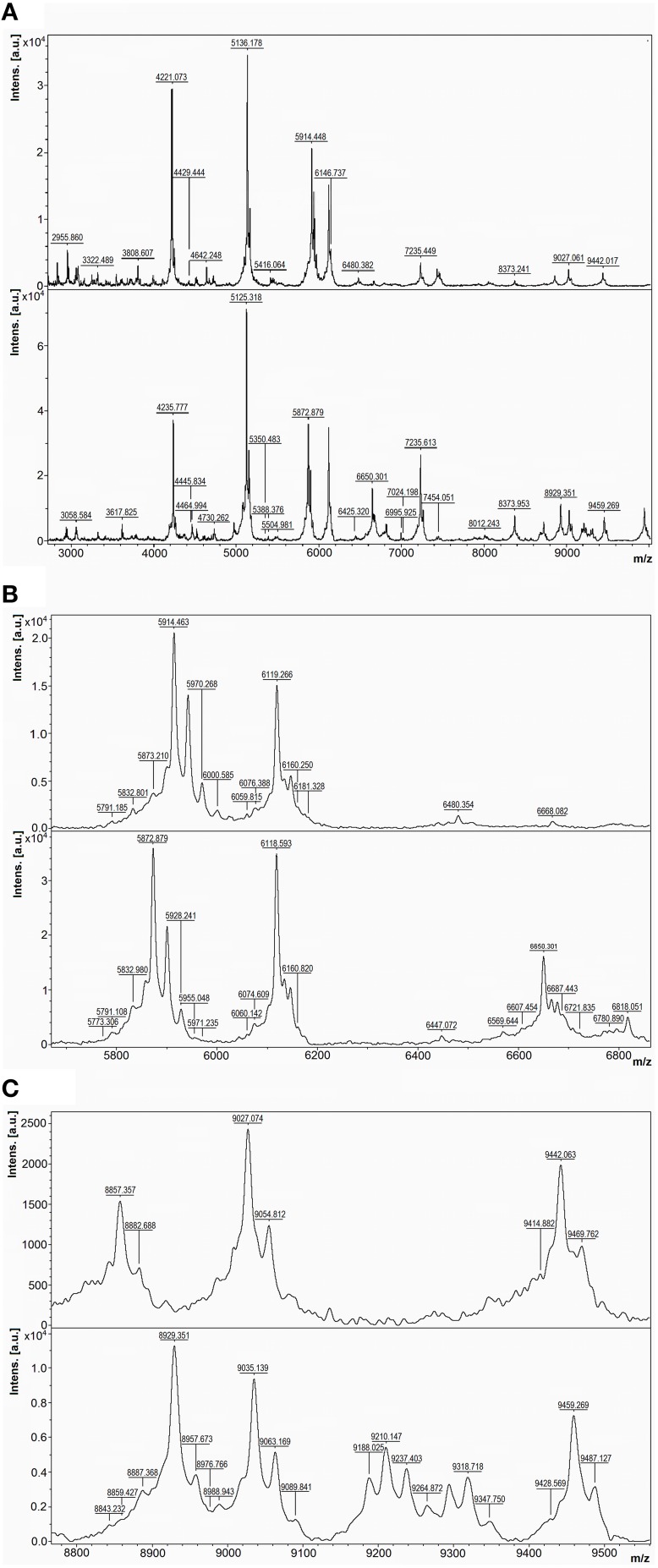
**Comparison of MALDI-TOF mass spectra of *P. tunicata* and *P. ulvae***. Top, *P. tunicata* DSM14096; bottom, *P. ulvae* DSM15557. **(A)** Mass spectra of the two type strains at *m/z* 2800-10,000, where the most peaks are. **(B)** Zoomed mass spectra showing peak shifts at *m/z* 5800–6000 and discriminative peaks at *m/z* 6400–6900. **(C)** Zoomed mass spectra showing discriminative peaks at *m/z* 8800–9500.

**Figure 4:**
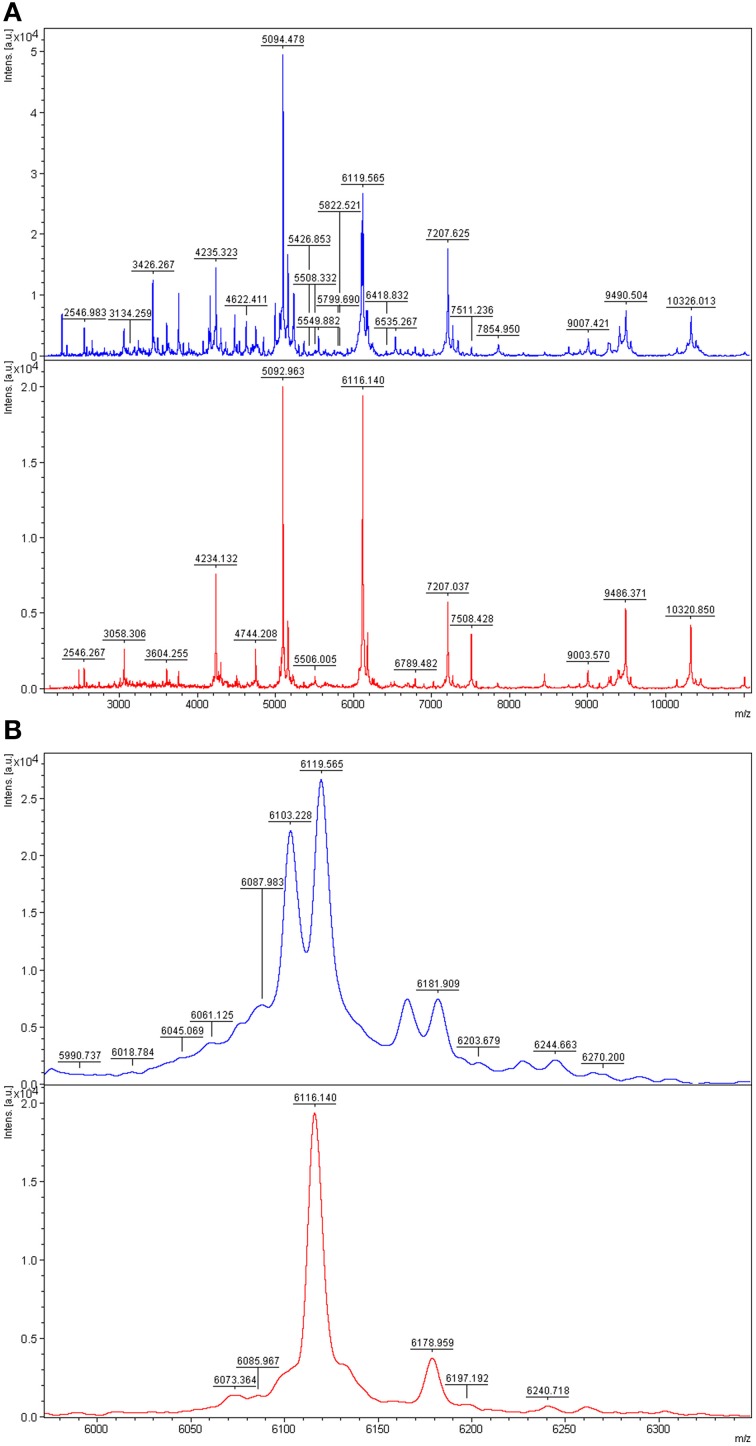
**Comparison of MALDI-TOF mass spectra of *P. aurantia* and *P. citrea***. Top, *P. aurantia* DSM6057; bottom, *P. citrea* DSM8771. **(A)** Mass spectra of the two isolates at *m/z* 2000–11,000, where the most peaks are. **(B)** Zoomed mass spectra showing discriminative peaks at about *m/z* 6103 and 6160 in the *P. aurantia* spectrum.

A good example of differences between the mass spectra of pairs of species that have related 16S rDNA sequences and related MALDI-TOF mass spectra is the case of *P. tunicata* and *P. ulvae*. The 16S rRNA gene sequences of these species have 98.0% identity and formed a well-supported clade (Figure 2). Their mass spectra clustered together (Figure 1) but the reciprocal Biotyper scores were < 1.70. Comparison of these mass spectra (Figure 3) showed that as well as several major shared peaks, *P. ulvae* had several unique peaks (e.g., *m/z* 6650, 8929, and 9180–9350) that clearly discriminate it from *P. tunicata*, which had a unique peak at *m/z* 8857, for example. The mass difference of 42 Da between groups of three peaks in *P. tunicata* (largest peak at *m/z* 5915) and *P. ulvae* (largest peak at *m/z* 5873) could be due to amino acid substitution events. Another example of the resolution of MALDI-TOF MS is the discrimination between *P. aurantia* and *P. citrea* (Figure 4). These species group together in Figures 1, 2. Their 16S rRNA genes have 99.8% identity over 1434 nucleotides. The MALDI-TOF MS spectrum of *P. aurantia*, however, contained peaks around *m/z* 6103 and 6165 that were different from the spectrum of *P. citrea* (Figure 4B). These differences were observed consistently, including in two sets of independent purchases from DSMZ, one in 2012 and the other in 2014.

**Figure 5:**
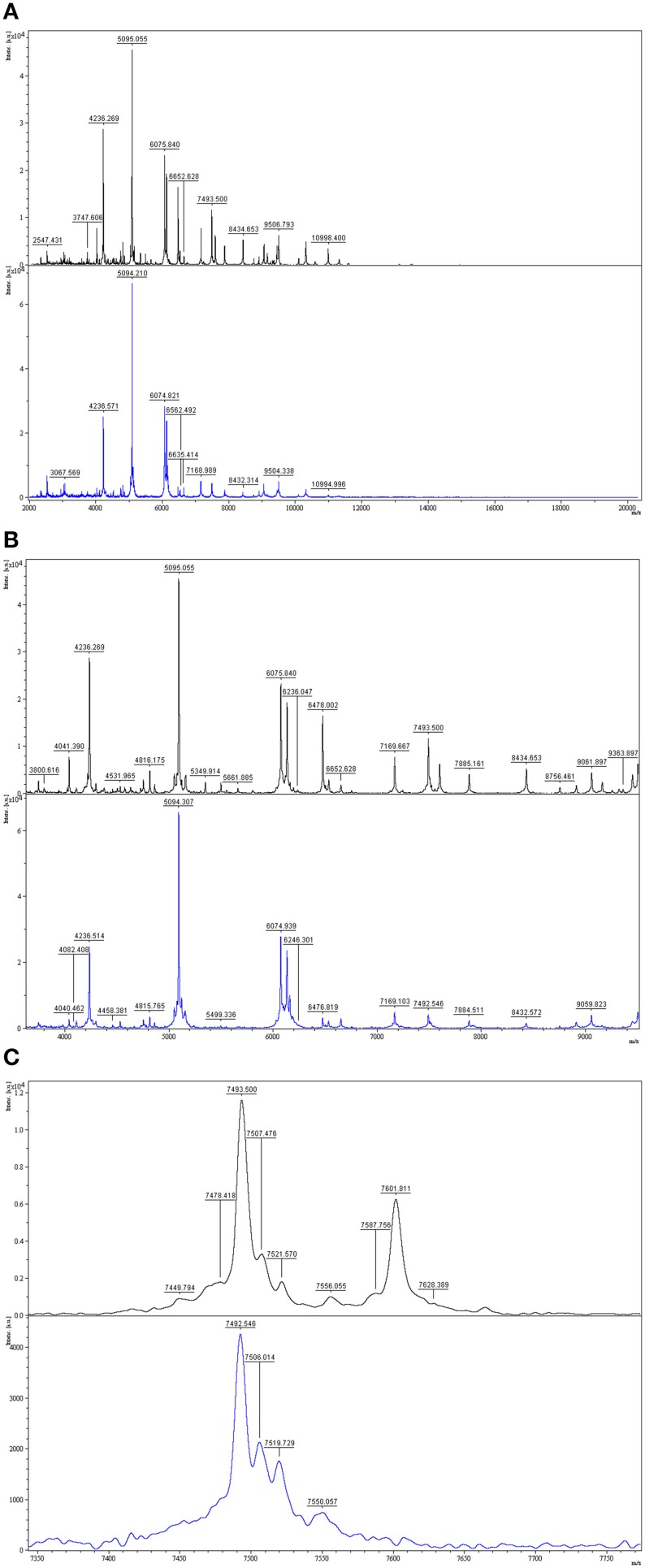
**Comparison of MALDI-TOF mass spectra of environmental isolate DG1628 and *P. carrageenovora***. Top, DG1628; bottom, *P. carrageenovora* DSM6820. **(A)** Mass spectra of the two isolates at *m/z* 2000-20000. **(B,C)** Zoomed mass spectra showing discriminative peak at *m/*z 7601 in the DG1628 spectrum.

In order to evaluate the efficacy of the in-house MALDI-TOF MS database for identification of unknown isolates, we tested the 13 environmental isolates that were identified as pseudoalteromonads by 16S rDNA sequencing. For each of these isolates, the identity of the known species with the most similar 16S rDNA sequence (Table 1) was determined by querying the EzTaxon database (Kim et al., 2012). All 13 isolates were also identified as pseudoalteromonads by MALDI-TOF MS biotyping, the identification being “secure” (score ≥2.00) in 11 cases and “probable” (score < 2.00 but ≥1.70) in two (Table 1). However, only three of the environmental isolates could be identified “securely” (score ≥2.30) to the species level (Table 1). DG1628 was identified as *P. carrageenovora* with a score of 2.41. Consistent with this score, DG1628 grouped with *P. carrageenovora* in the MALDI-TOF MS heat map and dendrogram (Figure 2). The only substantial difference between the two mass spectra was a peak at *m/z* 7602 in the DG1628 spectrum that was not present in the *P. carrageenovora* spectrum (Figure 5). The two 16S rRNA gene sequences were identical. The remaining isolates that were biotyped “securely” to the species level, DG1810 and NS-34, did not give concordant results by 16S rRNA gene analysis (Table 1). These isolates were identified as *P. haloplanktis* and grouped with *P. haloplanktis* in the MALDI-TOF MS dendrogram (Figure 1), whereas the 16S rDNA sequence of DG1810 was identical to that of *P. tetraodonis* and from its 16S rDNA sequence NS-10 was most closely related to *P. issachenkonii* and *P. tetraodonis*.

Among isolates with Biotyper scores < 2.30 for the closest match, isolates DG1838, NS-10, and NS-20 were identified as “probable” *P. haloplanktis* by MALDI-TOF MS and also clustered with DG1810, NS-10, and NS-34 in the MALDI-TOF MS dendrogram. The mass spectra of all these isolates formed a well-defined group (Figure 1) with *P. issachenkonii, P spiralis, P. undina*, and *P. tetraodonis*. From the 16S rDNA sequence, the closest relative of isolates DG1125 and DG1425 was identified as *P. marina*. The closest Biotyper match for isolate DG1152 was to *P. marina*, but with a score only just above 2.0 (Table 1), whereas the closest match for DG1425 was to *P. carrageenovora* with a similar score. In the dendrogram (Figure 1), each isolate was on the same branch as its most probable species. However, the heat map shows that the similarity between the mass spectra of DG1152 and DG1425 was higher than the similarity between the spectra of DG1152 and *P. carrageenovora*. Isolates NS-07 and NS-36 were not closely identified to any of the species in the database (highest Biotyper scores of 1.88 and 2.08, respectively), and their 16S rDNA sequences differed substantially from those of the closest species, *P. prydzensis*. However, both the MALDI-TOF MS and 16S rDNA sequence analysis linked these strains to their MALDI-TOF MS assigned species (Figures 1, 2). The mass spectra of isolates DG1664 and TG12 were most similar to each other's, but their highest Biotyper scores were for *P. carrageenovora* and *P. elyakovii*, respectively. The 16S rDNA sequence of isolate DG1664 was identical to that of *P. elyakovii*, while the species with the most similar sequences to that of TG12 were *P. arctica* and *P. translucida*. Finally, isolate DG1135 had a highly distinctive mass spectrum (Figure 1). Although the closest Biotyper match was to *P. translucida*, Figure 1 shows that these spectra did not cluster together. The species with the most similar 16S rDNA sequence in the EzTaxon database was *P. phenolica*, a pigmented species, which was not included in this study.

## Discussion

The availability of MALDI-TOF mass spectra for the majority of known *Pseudoalteromonas* species greatly increases the value of MALDI-TOF MS biotyping for this important genus. All the environmental *Pseudoalteromonas* isolates we examined can be identified to genus level with confidence, in many cases to a group of species, and in some cases to species level. Previous studies have only examined spectra of a few known *Pseudoalteromonas* species. For example, Emami et al. (2012) identified isolates from ballast water as *Pseudoalteromonas* by comparing their MALDI-TOF spectra with that of an isolate that was identified as *P. tetraodonis* by 16S rDNA sequence analysis. Without spectra of any other known species it was not possible to refine the identification.

Dieckmann et al. (2005) introduced the use of MALDI-TOF MS to distinguish *Pseudoalteromonas* species in a study of bacteria associated with sponges. They analyzed intact cells rather than cell lysates. Mass spectra from intact cells belonging to a wide range of genera, including *Pseudoalteromonas*, were compared by Salaün et al. (2010), but they did not report details of their spectra. Dieckmann et al. (2005) noted that peaks at *m/z* 4235, 6075, and 9220 seemed to be characteristic of the genus, but in their spectra the last of these is relatively weak. Most of our spectra have equivalent peaks at or near *m/z* 4235 and 6075, but not at 9220, indicating similarity but not identity between spectra of intact cells and of lysates. We have compared spectra of lysates and intact cells directly. They contained many common peaks and had similar discriminatory power but were not identical (data not shown). We chose to analyze lysates because this approach has several advantages over analysis of intact cells: identifications have been found to be more reliable (Schumann and Maier, 2014), cell suspensions in ethanol-water and cell lysates can be stored for further use whereas whole cell analysis needs freshly-grown bacteria, the risk of contaminating the instrument is reduced because bacteria are killed, and spreading of the sample on target plates is more uniform.

The spectra of most *Pseudoalteromonas* species have marked similarities, but some species have highly distinctive spectra with no Biotyper scores over 2.0 for any other *Pseudoalteromonas* species. Even so, the spectra share patterns of marker peaks that are characteristic of *Pseudoalteromonas*. The genus *Pseudoalteromonas* contains pigmented and non-pigmented species (Ivanova et al., 2014). The pigmented species fall into several groups, while the non-pigmented species are probably members of a single large clade with very similar 16S rDNA sequences (Ivanova et al., 2014). The pigmented species tend to produce bioactive compounds, whereas the non-pigmented ones tend to produce enzymes of biotechnological significance (Bowman, 2007). It should be noted, however, that some “non-pigmented” species have the ability to form pigments on some media (Ivanova et al., 2004b). Most of the more distinctive mass spectra (*P. aurantia* and below in Figure 1) belong to pigmented species that have the most divergent 16S rDNA sequences as well. A notable exception is *P. paragorgicola*, which has a highly distinctive MALDI-TOF mass spectrum even though it is a member of the group of species with highly similar rDNA sequences. Interestingly, this species is pigmented (Ivanova et al., 2002), whereas its closest relatives are predominantly non-pigmented (Ivanova et al., 2014). Thus, it seems that *P. paragorgicola* has evolved in a distinctive way. Analysis of the peptides making up the MALDI-TOF MS spectrum could shed light on why the spectrum is so distinctive. In any case, the *P. paragorgicola* spectrum illustrates that although species with similar MALDI-TOF MS spectra are generally related to one another, the converse is not necessarily true: closely-related species can have dissimilar spectra.

MALDI-TOF MS is capable of discriminating between directly related *Pseudoalteromonas* species such as *P. tunicata* and *P. ulvae*, whose 16S rRNA genes have 98.0% identity (Figures 1, 3). The ability to discriminate between these species illustrates the practical potential of the method, as *P. tunicata* has been found to have substantially greater antifouling activity than *P. ulvae* (Holmström et al., 2002). This level of discrimination is similar to that observed by Salaün et al. (2010). Directly related species had distinctive spectra even when they had nearly identical 16S rRNA gene sequences. As a well-defined example, scrutiny of the mass spectra of *P. citrea* and *P. aurantia* revealed biomarker peaks in their spectra that could be used to differentiate between the two species, which share a 16S rRNA gene sequence identity of 99.8% (Figure 4). Similarly, Dieckmann et al. (2005) found that *Pseudoalteromonas* species with only three base pair differences over 1500 base pairs of 16S rRNA genes could be differentiated by their MALDI-TOF mass spectral patterns.

Because of the rapidity and sensitivity of MALDI-TOF MS, it has considerable potential for application in large-scale screening for isolates that could produce novel bioactive molecules and enzymes to add to those that have already been found in *Pseudoalteromonas* species. At present, the extent of diversity within *Pseudoalteromonas* is unknown—a crucial knowledge gap that is slowing the biotechnological exploitation of this genus as well as our capacity to further elucidate its ecological roles. New species are being described gradually: one in the *International Journal of Systematic and Evolutionary Microbiology* in 2013, two in 2014, for example (Matsuyama et al., 2013, 2014; Zhao et al., 2014), while the GenBank/EMBL/DDBJ DNA sequence database contained more than 4000 16S rRNA gene sequences identified only as “*Pseudoalteromonas* sp.” in July 2015. Thus, when a new isolate is found, a key question is whether or not it belongs to a known species. Only one of our isolates can be identified to the species level with high confidence: combining MALDI-TOF MS and 16S rDNA sequence analysis indicates that isolate DG1628 is *P. carrageenovora*, a non-pigmented species that was the first organism found to be able to degrade λ-carrageenan (Weigl and Yaphe, 1966). Identification of *P. carrageenovora* from Scottish waters adds to accumulating evidence that this species is globally widespread. The type strain was collected near Halifax, Nova Scotia, Canada because of its ability to degrade carrageenan (Yaphe and Baxter, 1955; see also https://www.dsmz.de/catalogues/details/culture/DSM-6820.html). Subsequently, it has been reported from Japan (Nandakumar et al., 2002), and there are sequence database entries from South Africa, Indonesia, and China.

Besides DG1628, two other isolates (DG1152, DG1664) have the same closest species by both 16S rDNA sequence and MALDI-TOF biotyping (*P. marina* and *P. elyakovii*, respectively), and have rDNA sequences that are identical (DG1164) or nearly identical (DG1152) to the type strain sequence, suggesting that they can be identified to the species level. However, the highest Biotyper scores for both are ~2.0, which implies that these isolates could be different species despite the close similarity of their 16S rDNA sequences. Isolate NS-36 also has the same closest species, *P. prydzensis*, by 16S rDNA sequence and MALDI-TOF biotyping. However, the NS-36 16S rDNA sequence differs from the *P. prydzensis* type strain sequence by 19 bases, enough to indicate that it is a different species. The MALDI-TOF mass spectrum of isolate NS-07 is similar to that of NS-36. The mass spectra of these two isolates are more similar to one another than to the mass spectrum of any known species, but their 16S rRNA gene sequences differ at 17 positions out of 1358. Among known species, NS-07 is most closely related to *P. prydzensis* according to its rDNA sequence, whereas its MALDI-TOF mass spectrum is most similar to that of *P. mariniglutinosa*. *P. prydzensis* and *P. mariniglutinosa* themselves have similar MALDI-TOF mass spectra. From their 16S rDNA sequences, these two species, isolates NS-07 and NS-36 and several other species form a well-defined clade. The combination of 16S rDNA sequences and MALDI-TOF spectra suggests that isolates NS-07 and NS-36 represent distinct, novel species in this clade. Further polyphasic analysis will be needed to test this conclusion.

For several isolates, the species with the most similar MALDI-TOF mass spectrum is different from the species with the most similar 16S rDNA sequence. Isolates NS-10, NS-20, NS-34, DG1810, and DG1838 have very similar MALDI-TOF mass spectra, and the type strain with the most similar mass spectrum is *P. haloplanktis*. However, from their 16S rRNA gene sequences, their closest known relatives are *P. tetraodonis, P. issachenkonii, P. undina*, and *P. elyakovii*, respectively. All these type strains and environmental isolates are in the large group with very similar 16S rDNA sequences, so that their phylogeny is poorly resolved. Thus, the occurrence of discrepancies between evidence from different methods is not surprising. The type strains of *P. tetraodonis, P. issachenkonii, P. undina*, and *P. elyakovii* have MALDI-TOF mass spectra that are more similar to each other's than to those of any of the new isolates. Thus, isolates NS-10, NS-20, NS-34, DG1810, and DG1838 may represent one or more new species.

In some cases, the closest similarity of spectra is between environmental isolates. There is a clear pairing between the spectra of isolates DG1664 and TG12, for both of which the best (but relatively low) Biotyper match is to *P. elyakovii*. However, these isolates have distinct 16S rDNA sequences (98.7% identity), so that they are unlikely to represent a single new species. Another complex case is that of isolate DG1425. This is most closely related to *P. marina* according to its 16S rDNA sequence, but the highest Biotyper score is for *P. carrageenovora*. However, there is a link between the mass spectra of isolate DG1425 and *P. marina*: the highest CCI for DG1425 is with the spectrum of isolate DG1152, which itself is most similar to that of *P. marina*.

The only environmental isolate in this study that is outside the large group of closely-related and mostly non-pigmented *Pseudoalteromonas* species according to its 16S rDNA sequence is DG1135. The closest match by biotyping is to *P. translucida*, but inspection of the heat map and dendrogram indicates that this resemblance is misleading: the CCI distances between the DG1135 spectrum and most other spectra are relatively high. In the dendrogram the spectrum is linked distantly to those of *P. lipolytica* and *P. arabiensis*. This result shows that the profile of resemblances of spectra to multiple species provides additional information beyond that obtainable from a simple biotyping analysis.

In conclusion, the availability of a database of MALDI-TOF mass spectra for *Pseudoalteromonas* type strains makes it possible to identify new isolates as *Pseudoalteromonas* species with confidence and in many cases to identify related species, but identification to the species level is typically not possible. This is only partly because of the high diversity of the MALDI-TOF mass spectra. An important reason is the extent of biodiversity that is still uncharted even within this culturable genus, so that the isolates tested probably represent undescribed species. Definition of new bacterial species currently requires polyphasic methods combining biochemical data, phylogenetic analysis of appropriate genes and DNA-DNA hybridization (Vandamme et al., 1996). Conversely, definitive identification of organisms as members of known species requires application of at least a subset of these polyphasic methods. Although genome sequencing may replace DNA hybridization (Chun and Rainey, 2014; Ramasamy et al., 2014), it will be some time before it can be routinely applied to large numbers of isolates. Analysis of 16S rDNA sequences is very powerful for identifying taxa, but in the absence of additional evidence is not definitive. MALDI-TOF MS introduces additional phenotypic markers (Dieckmann et al., 2005; Ng et al., 2013) and is fast and highly discriminatory. Moreover, because it represents several gene products, MALDI-TOF MS reflects a higher proportion of the genome than 16S rDNA. At least some of the differences between patterns of 16S rDNA sequence relatedness and MALDI-TOF mass spectral similarity may be due to differences in the evolutionary history of the relevant genes; intra-genomic evolutionary diversity is being increasingly recognized in bacteria (Kamneva and Ward, 2014). Further investigation of *Pseudoalteromonas* genome evolution and of the proteins responsible for the MALDI-TOF mass spectra should elucidate the causes of the differences. In any case, MALDI-TOF mass spectra should ideally be a component of taxonomic description, for which data sharing and public availability of data are essential: there is a need for a user-friendly way of sharing data files between researchers. Developing such a system remains a challenge. Once sufficient data are available, MALDI-TOF MS analysis can take its place as a key step in identification of marine bacterial isolates. Isolates that give low Biotyper scores with known species or where the MALDI-TOF MS and 16S rRNA gene phylogeny do not agree should be further investigated as they are likely to represent novel species.

## Author contributions

KE Conceived, designed, and conducted the study, analyzed the data and co-wrote and approved the manuscript. AN Designed the work, conducted, analyzed, and interpreted the 16S rRNA work and co-wrote and approved the manuscript. EH Conceived, analyzed, and interpreted the phylogenetic data and co-wrote and approved the manuscript. JZ Contributed environmental isolates, contributed to discussion and interpretation of data, and approved the manuscript. DG Contributed environmental isolates, conceived the project and interpreted, and co-wrote and approved the manuscript. GC Designed the project, interpreted the data, co-wrote, and approved the manuscript and contributing funding support. EM Contributed funding support, interpreted data, co-wrote, and approved the final manuscript.

### Conflict of interest statement

KE is Director of Pakogreen Ltd., that contributed materially to this study. The other authors declare that the research was conducted in the absence of any commercial or financial relationships that could be construed as a potential conflict of interest.

## Supplementary material

Supplementary Figure 1 **Mass spectra of *Pseudoalteromonas* type strains**. The strains are in alphabetical order as listed in the Materials and Methods Section.

Supplementary Figure 2 **Overlaid mass spectra of *Pseudoalteromonas* type strains where the most common peaks were observed**. Nearly all the isolates have a peak at *m/z* 4236 **(A)** and most of them have a peak at *m/z* 5095 **(B)**.
