# Supplementary figures and images for: MALDI-TOF Mass Spectrometry Discriminates Known Species and Marine Environmental Isolates of Pseudoalteromonas
Source: Front Microbiol. 2016 Feb 12;7:104. doi: 10.3389/fmicb.2016.00104 (PMC4751257; doi:10.3389/fmicb.2016.00104)

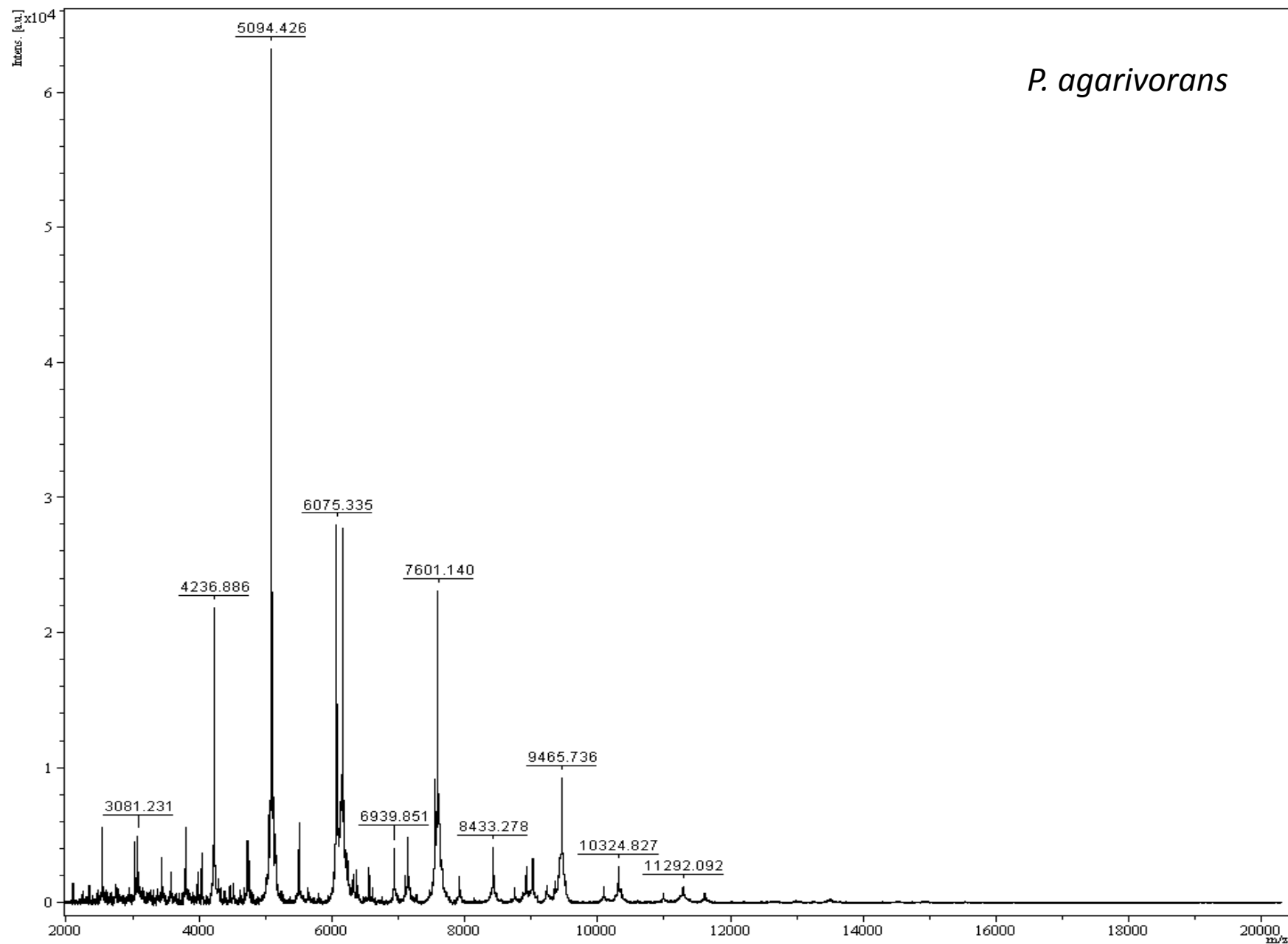

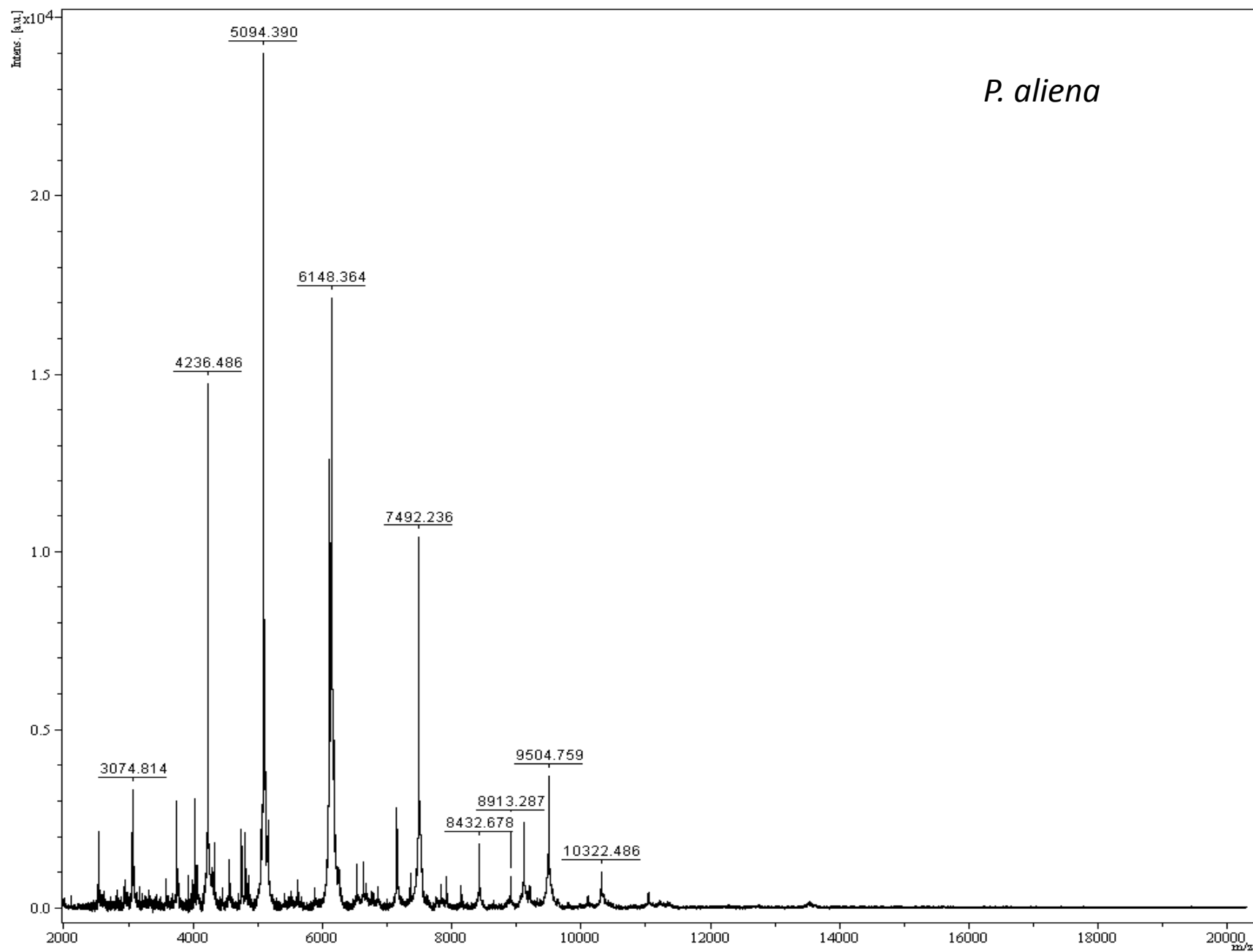

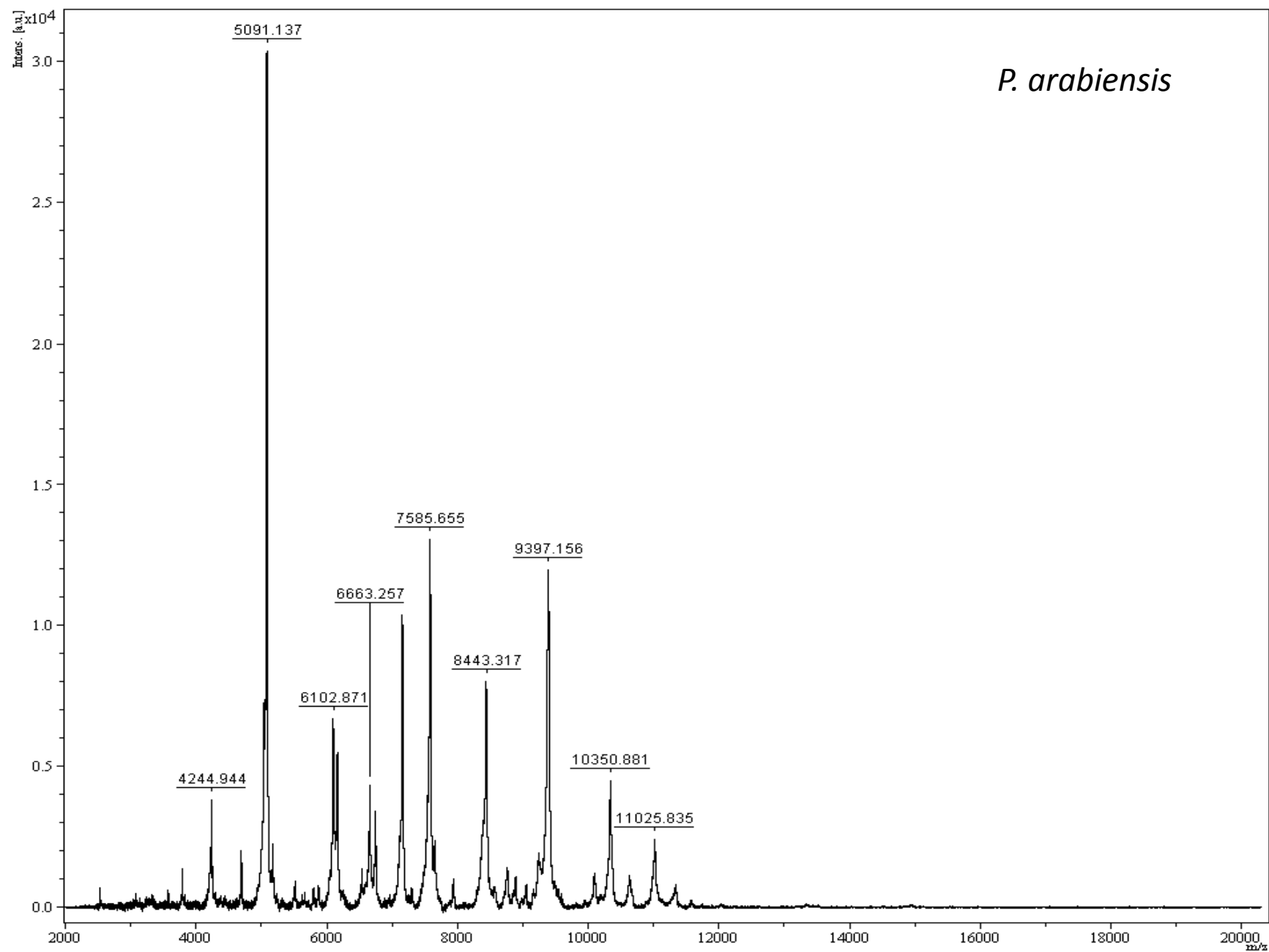

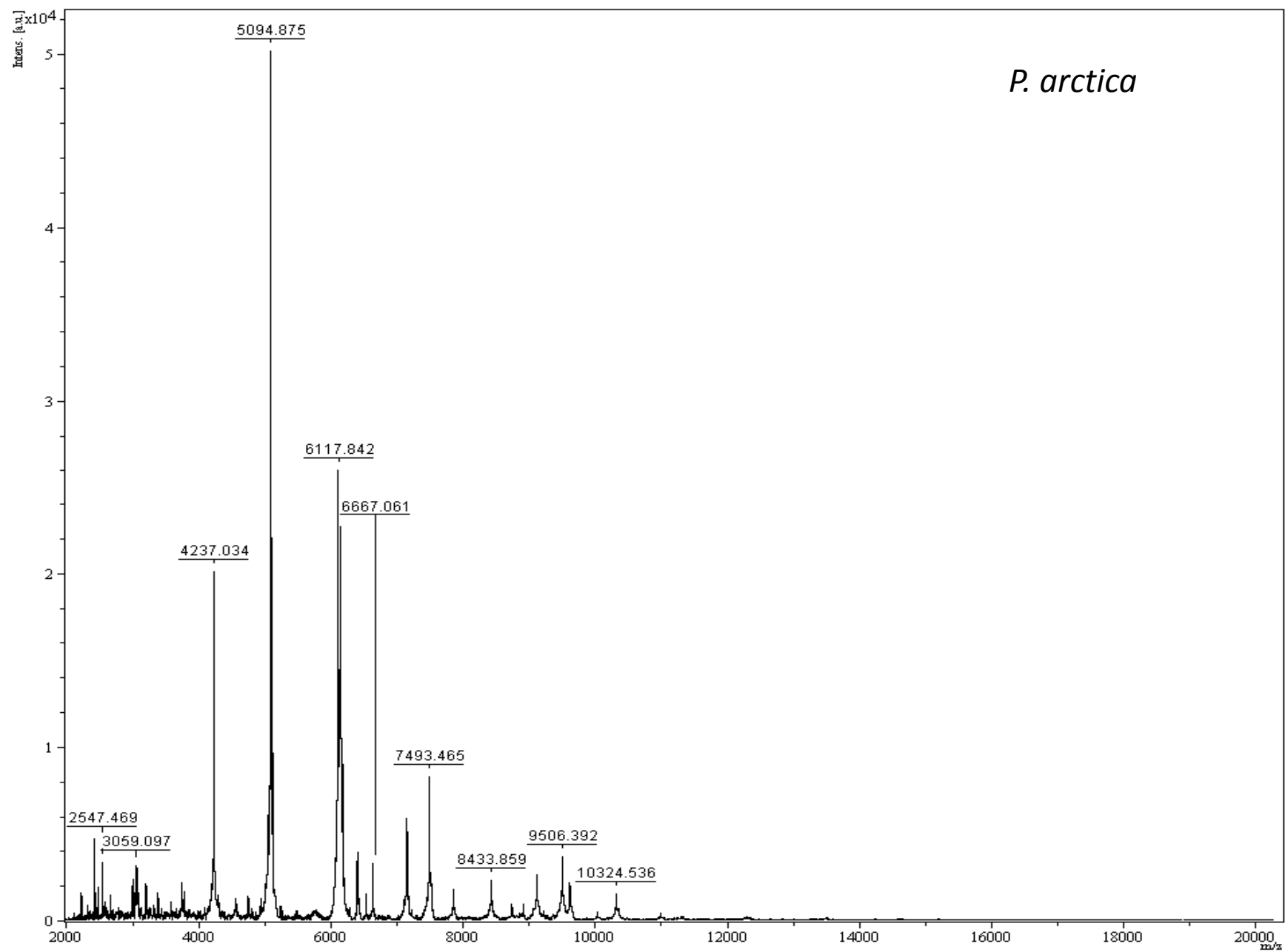

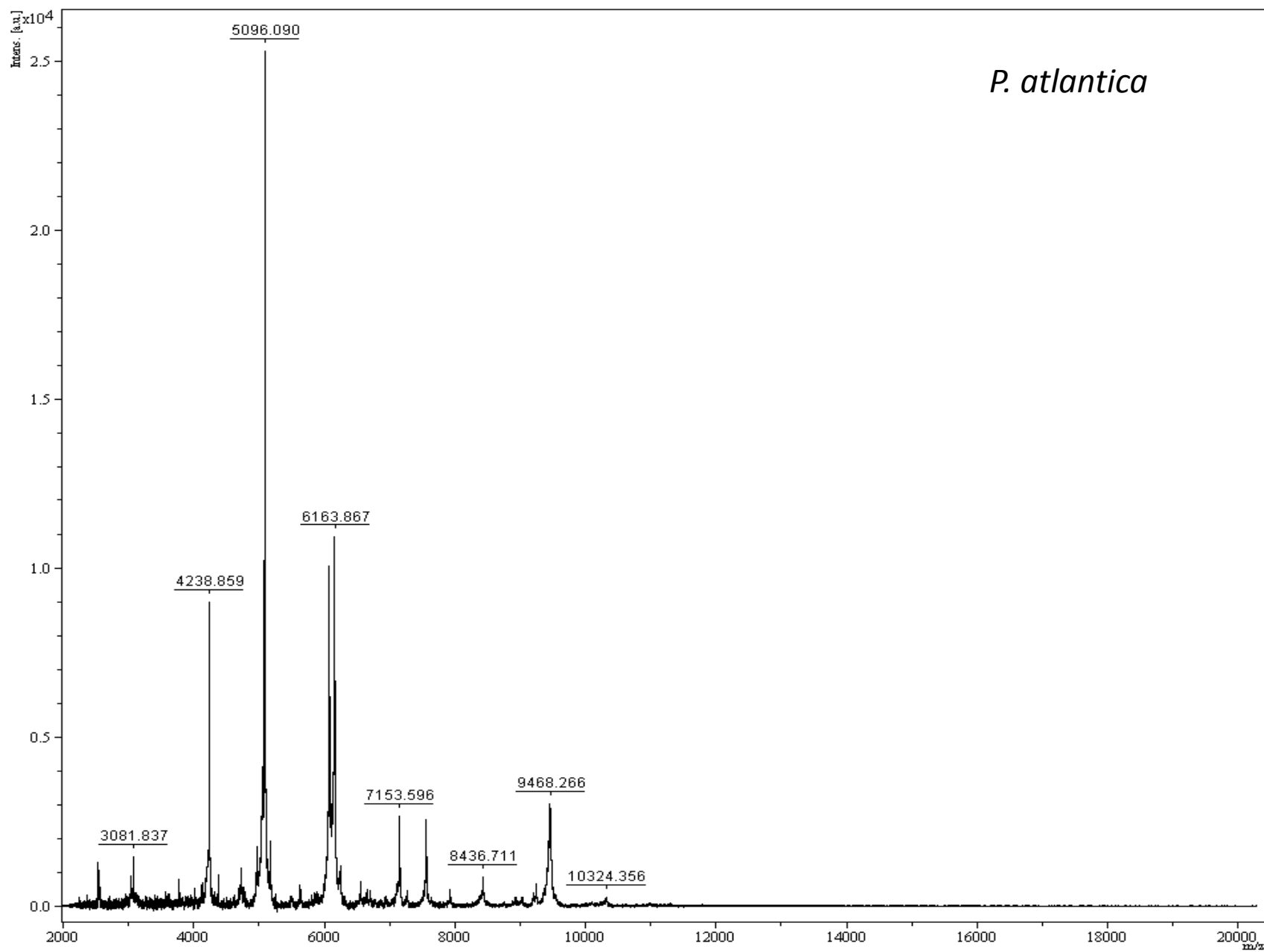

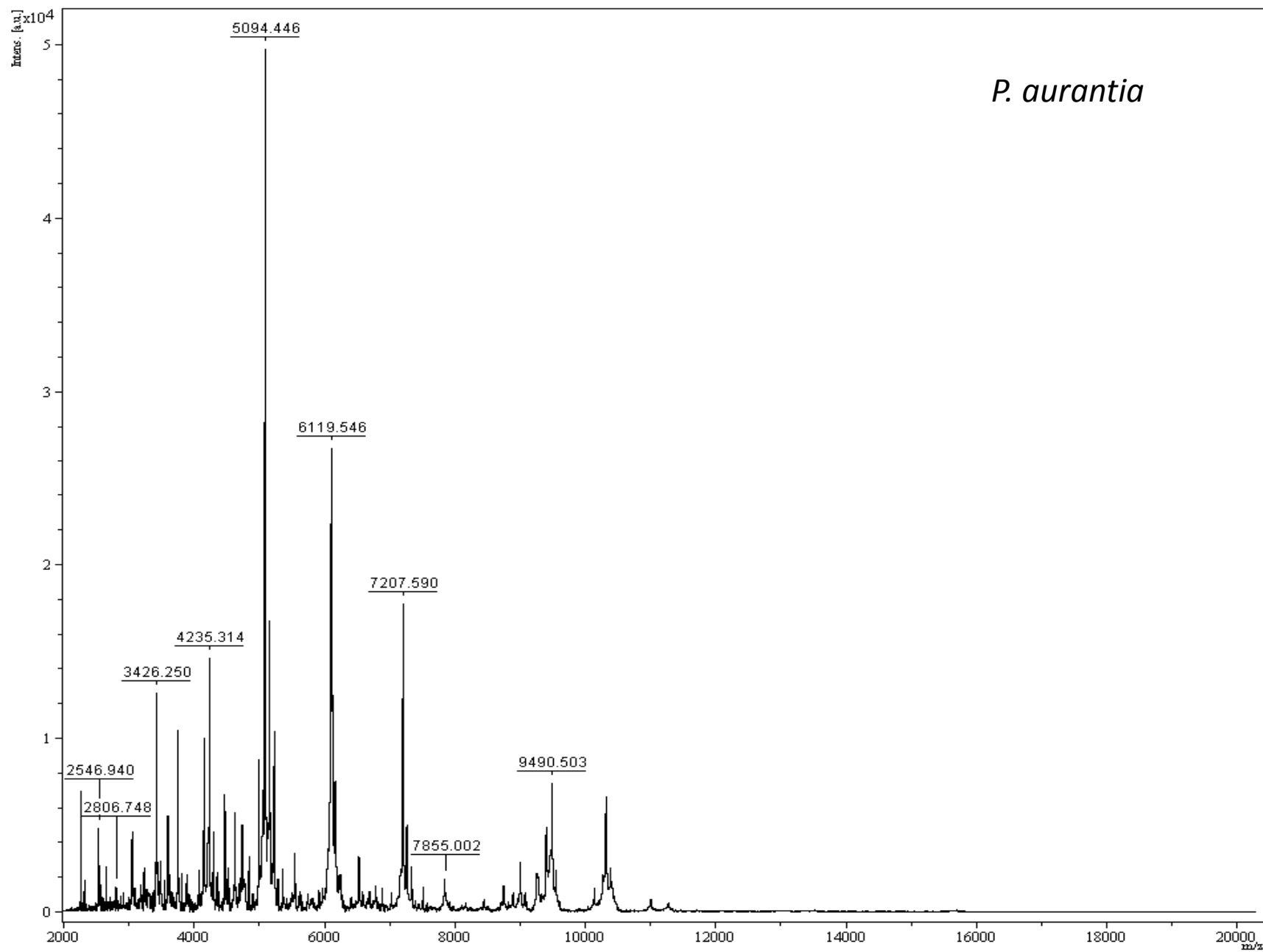

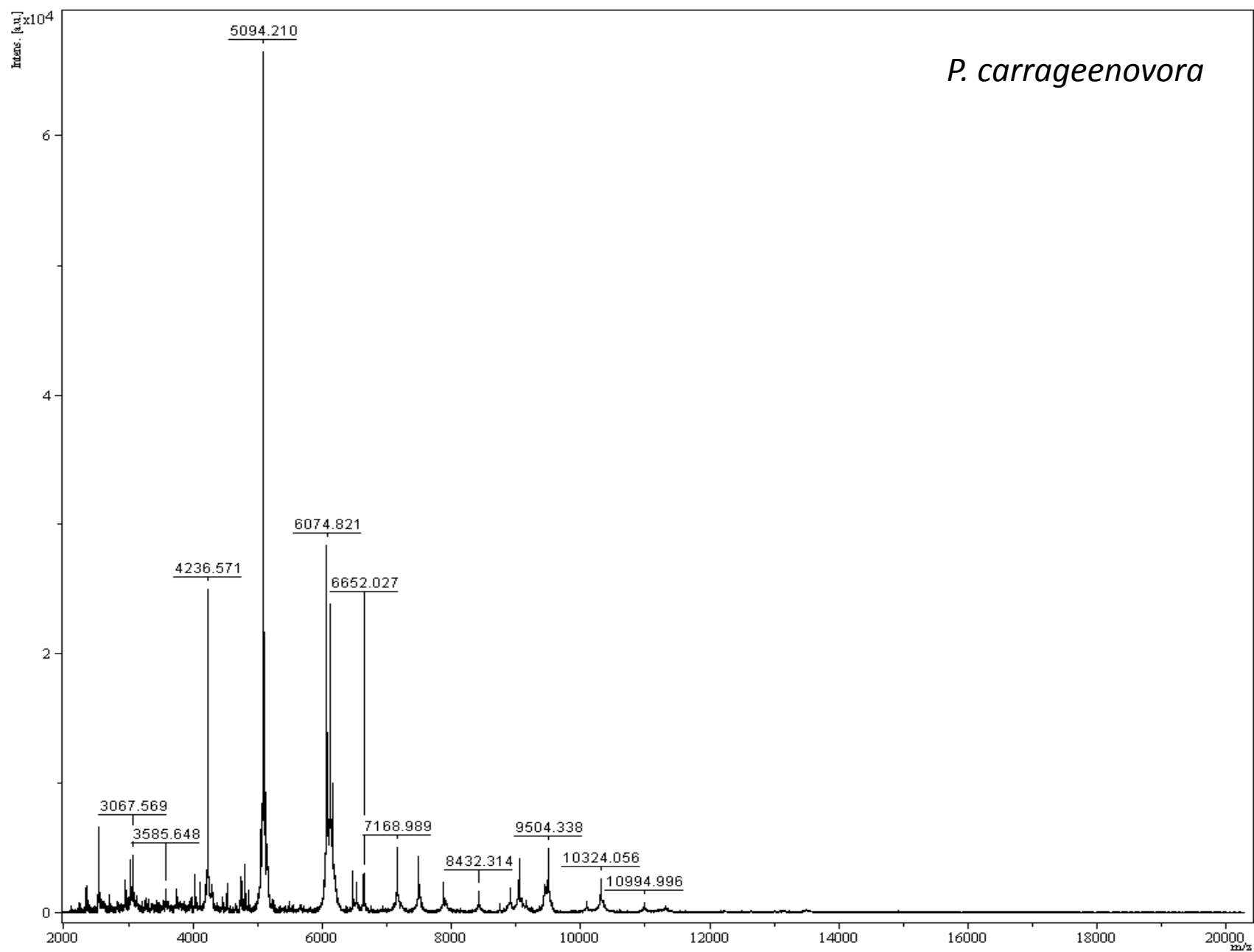

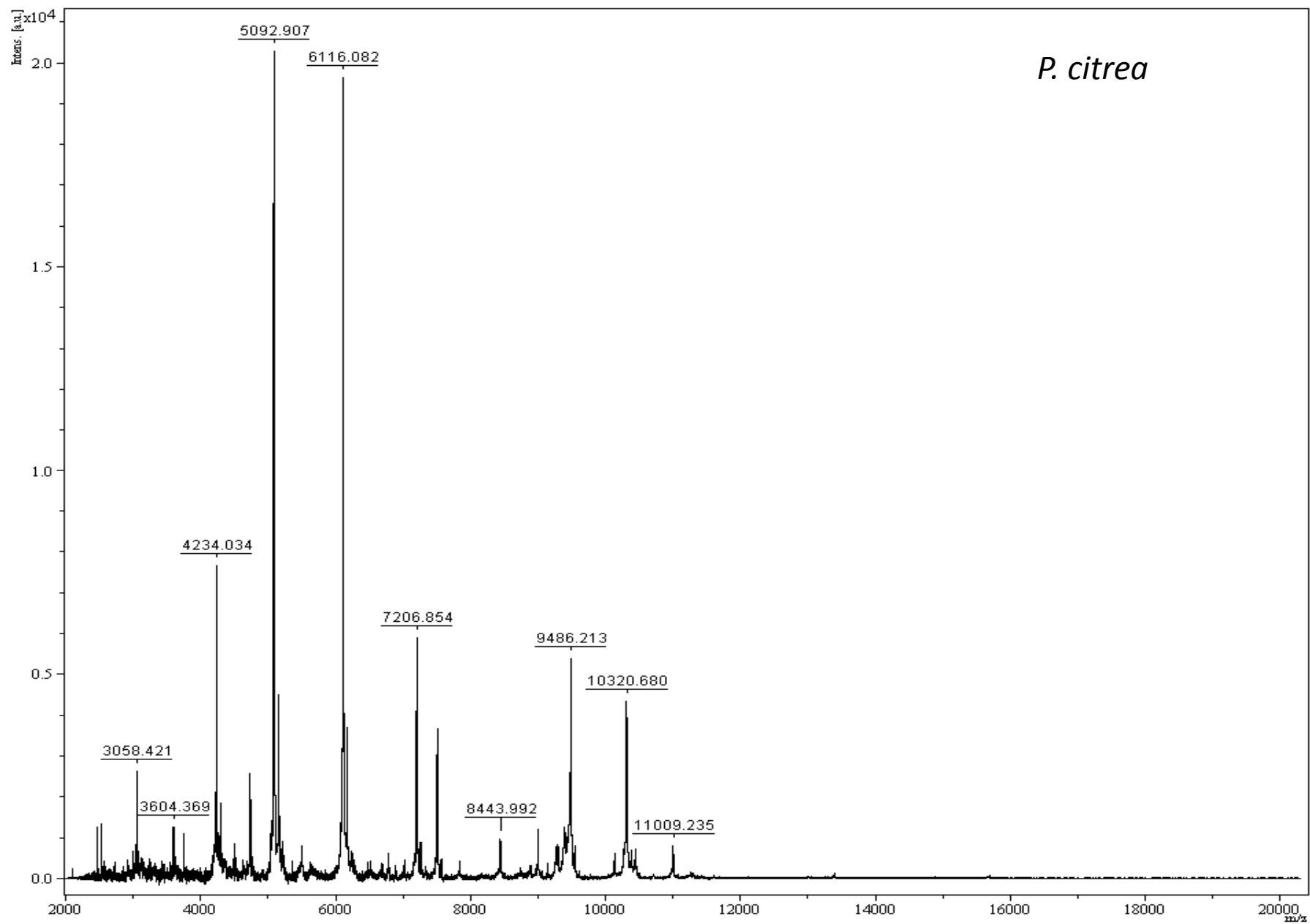

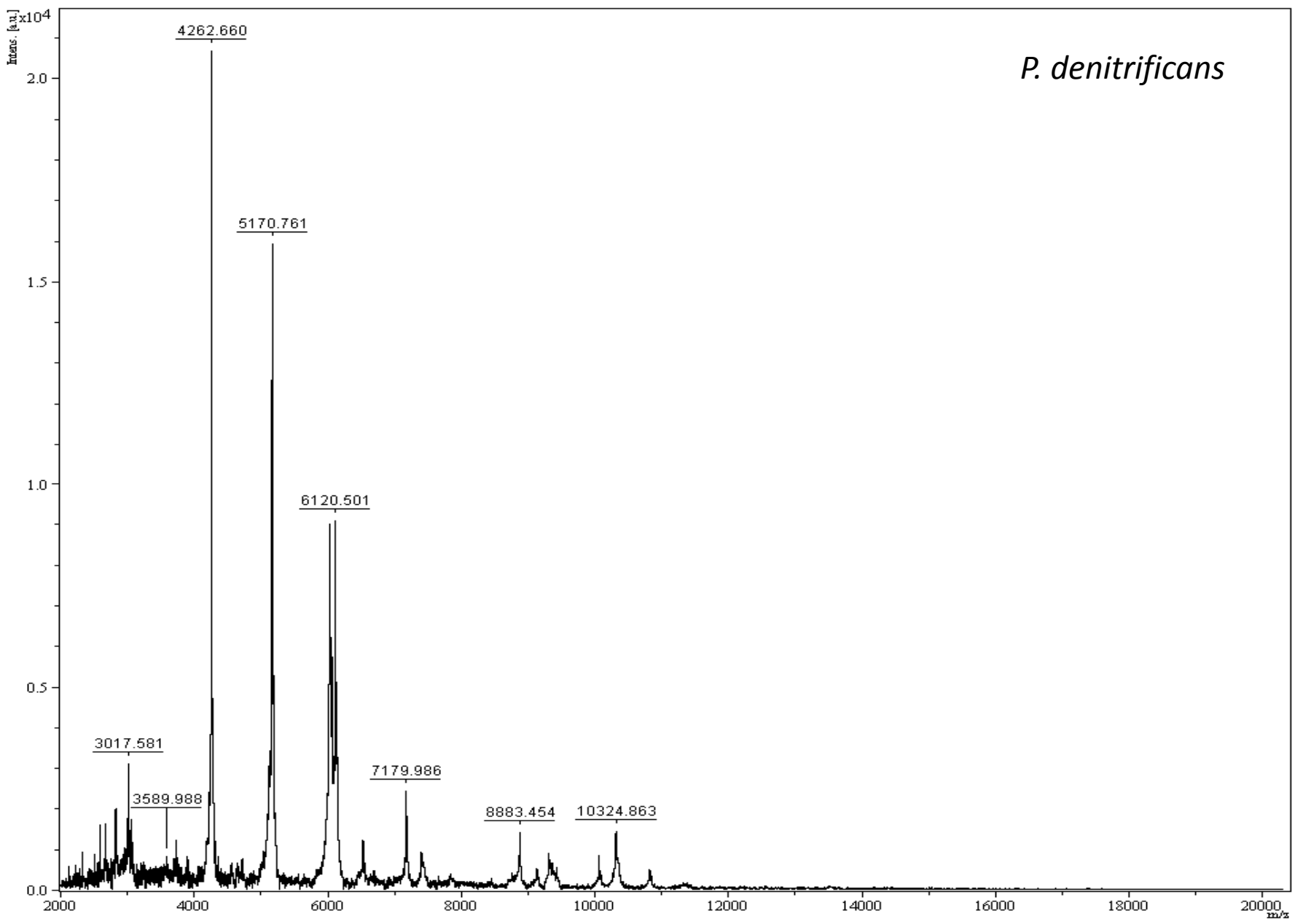

*P. denitrificans*

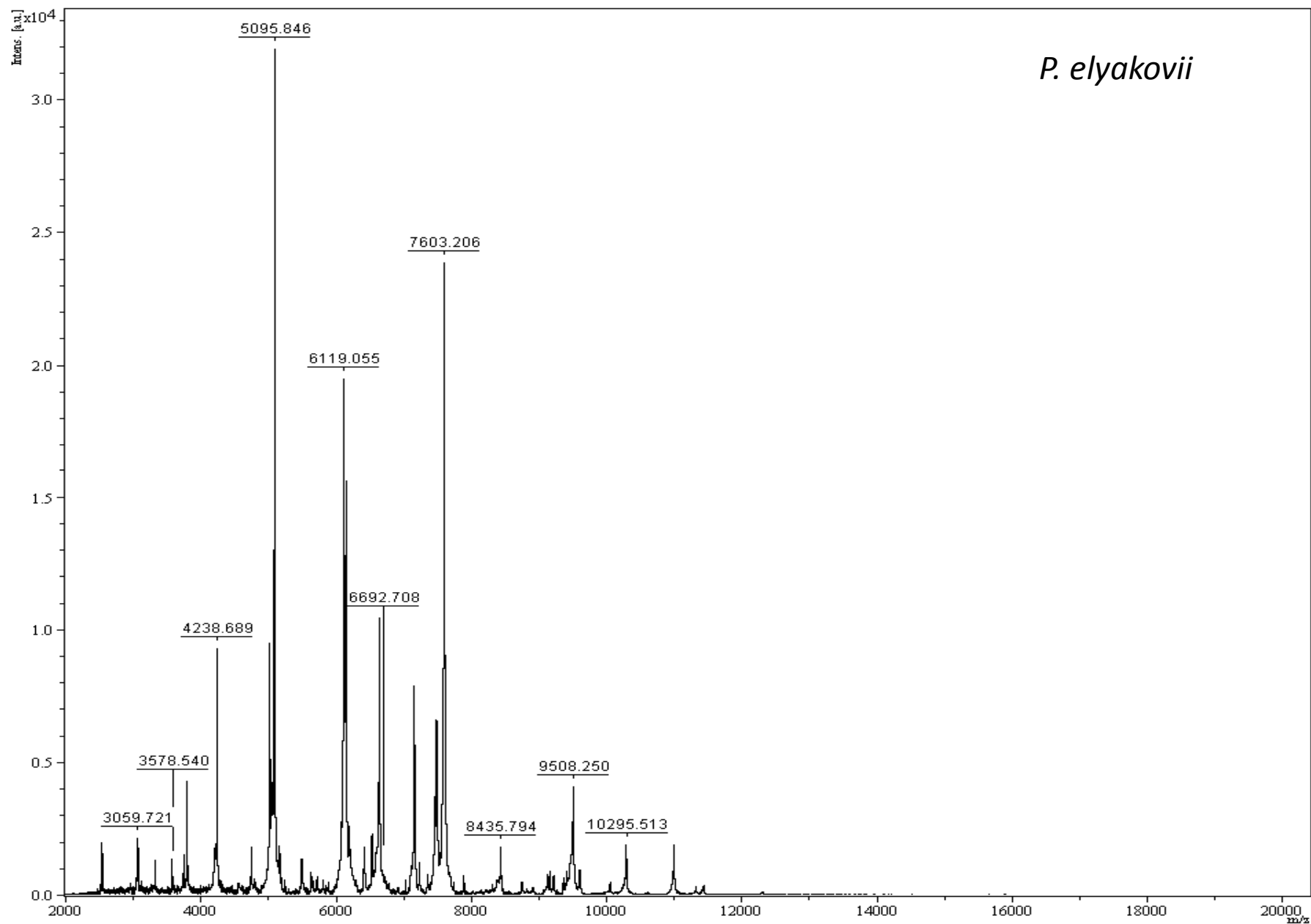

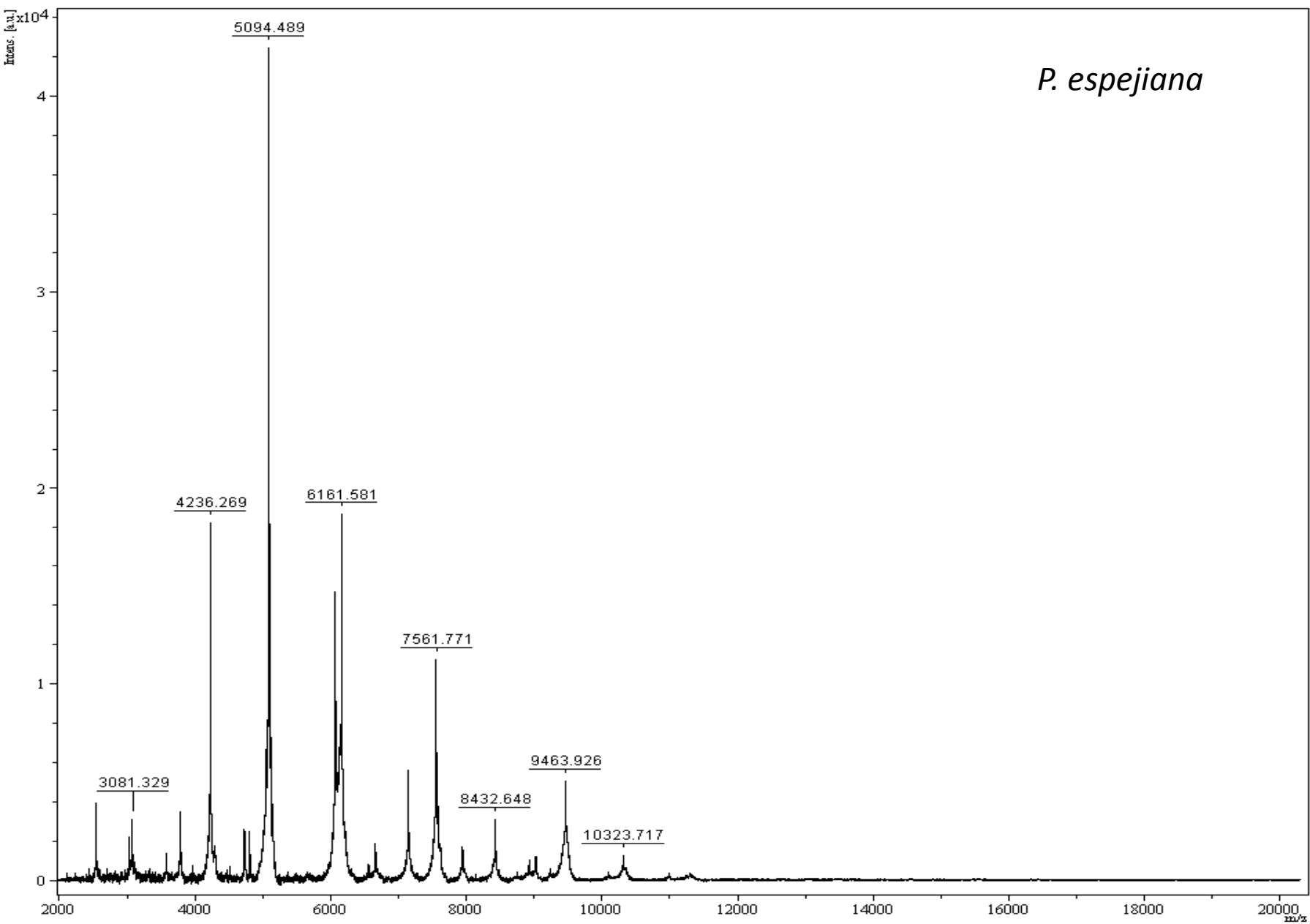

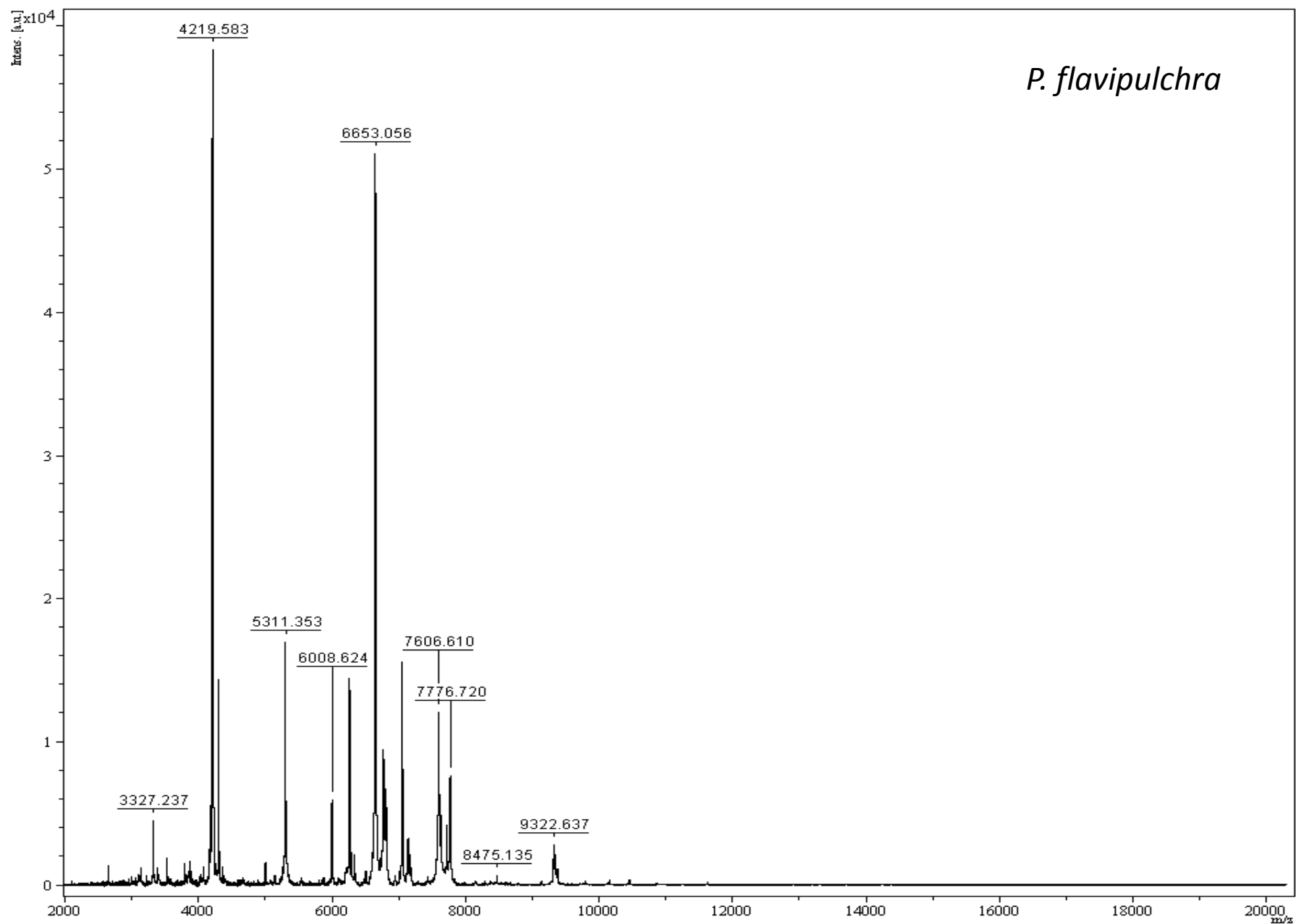

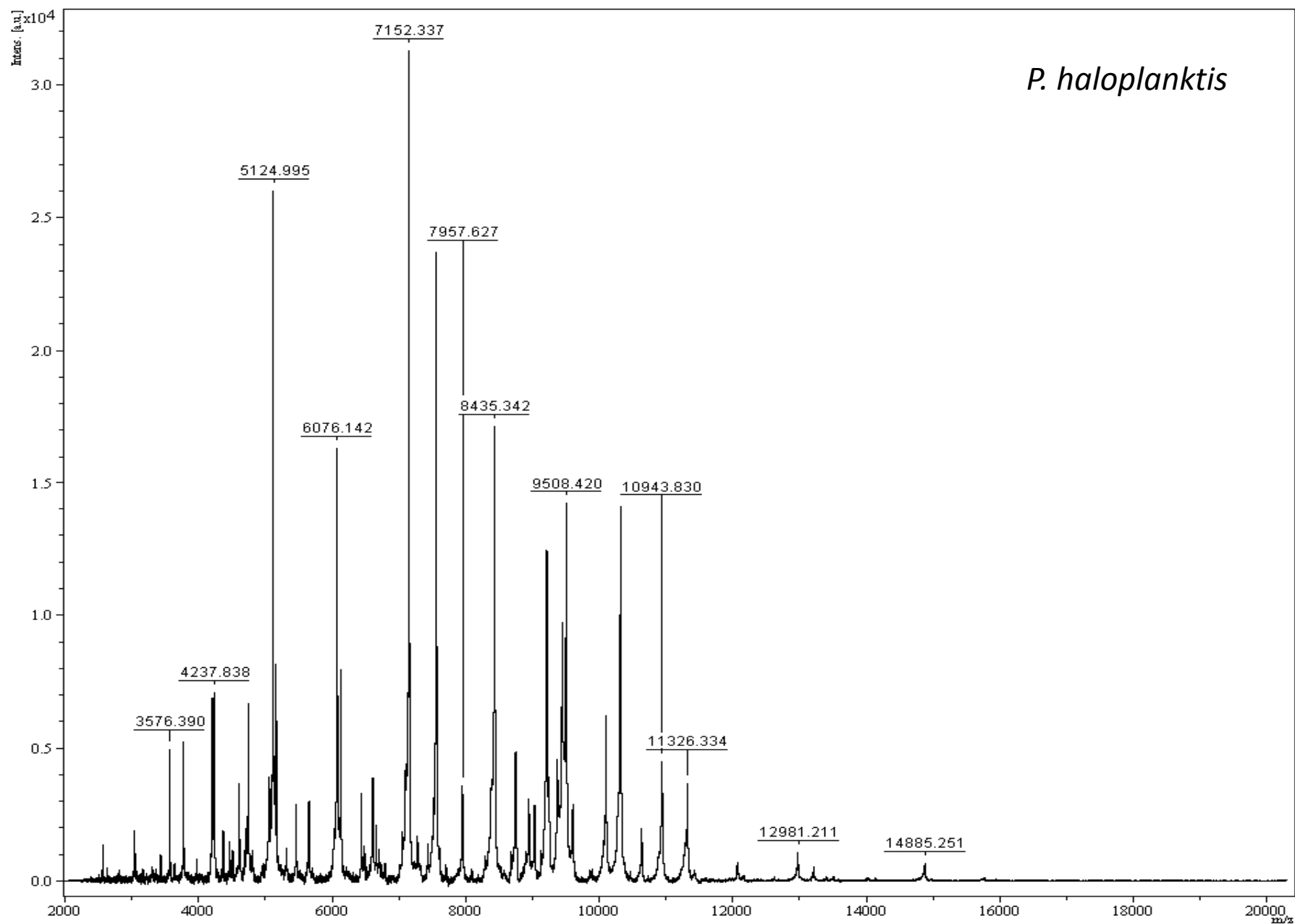

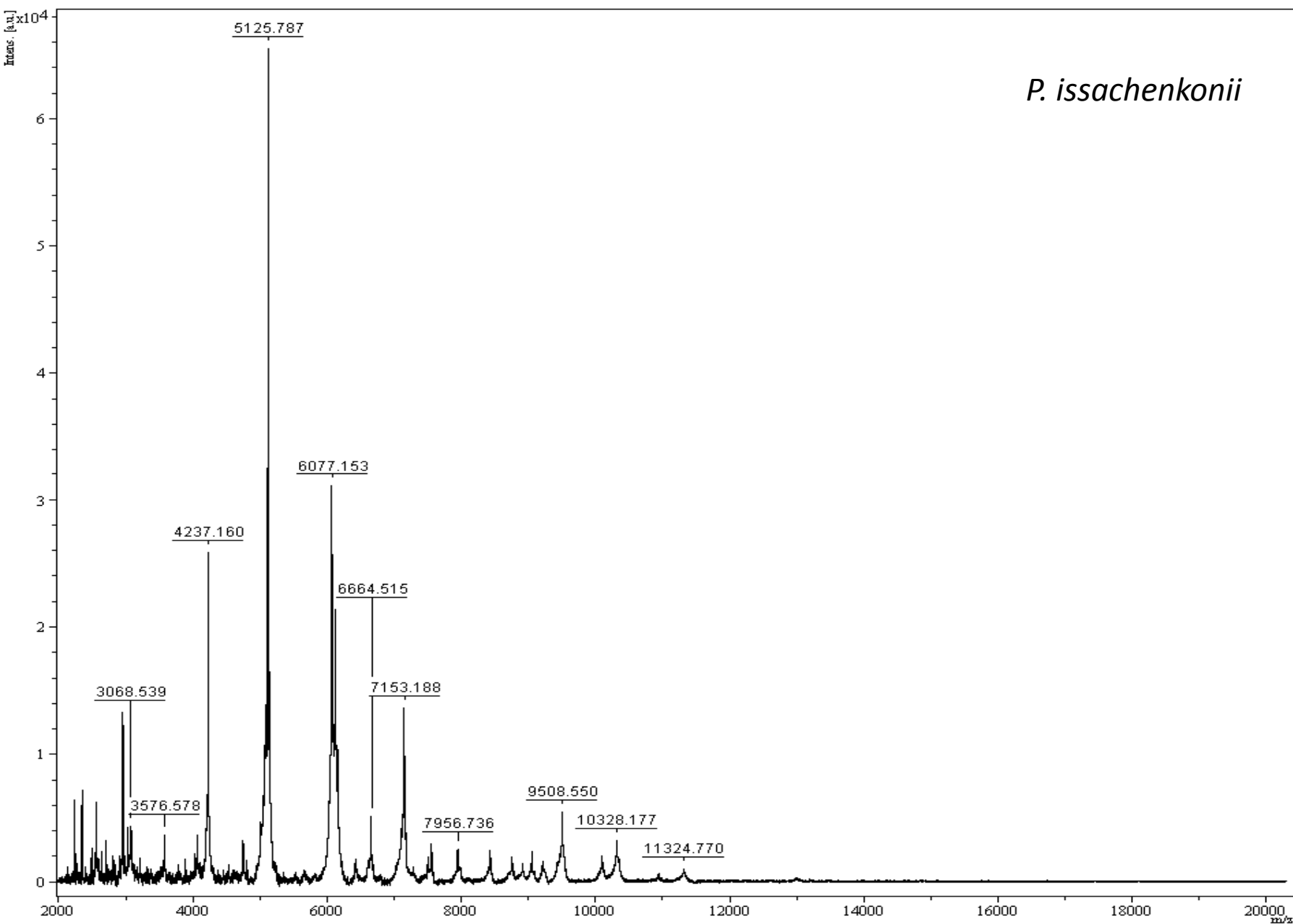

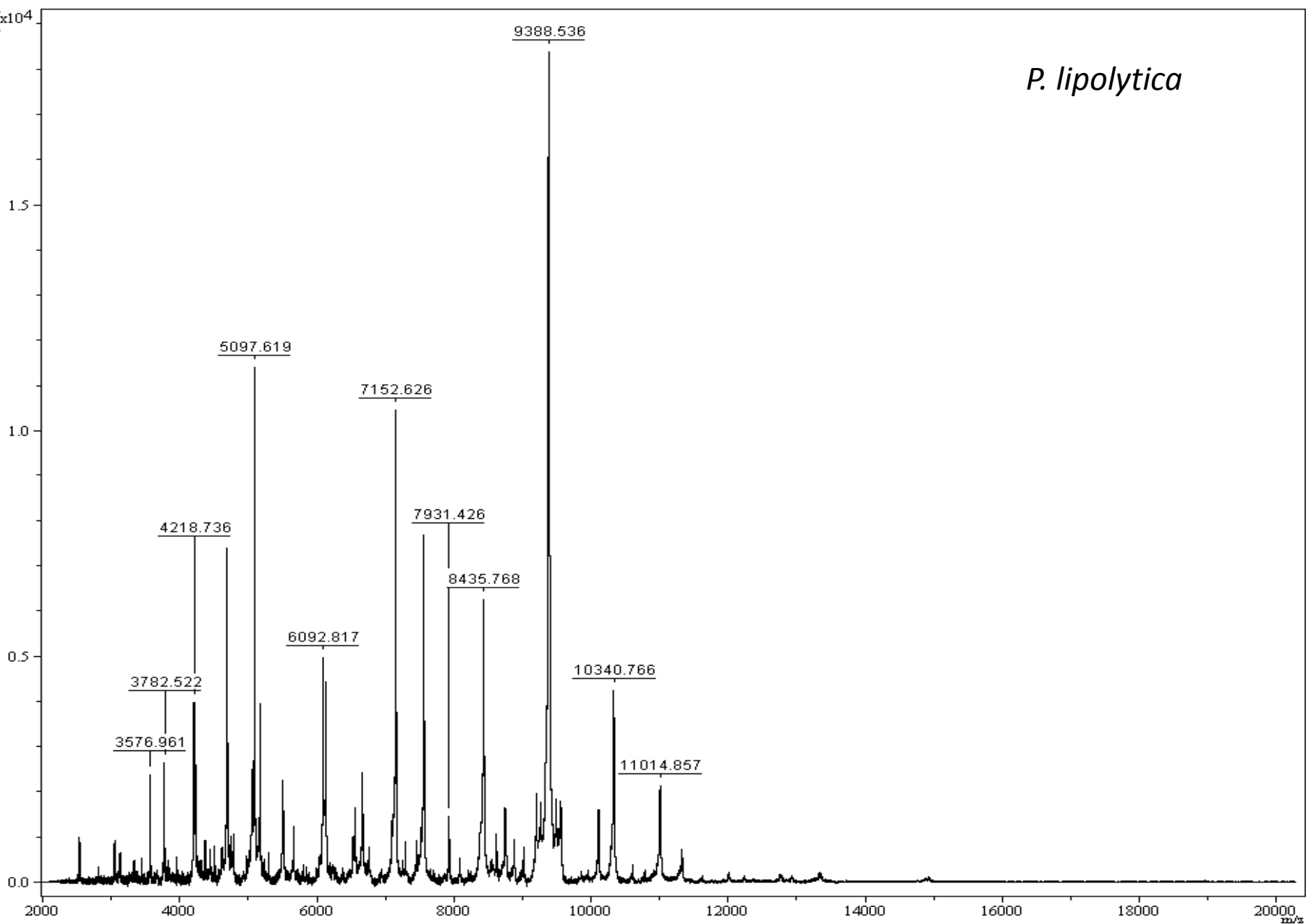

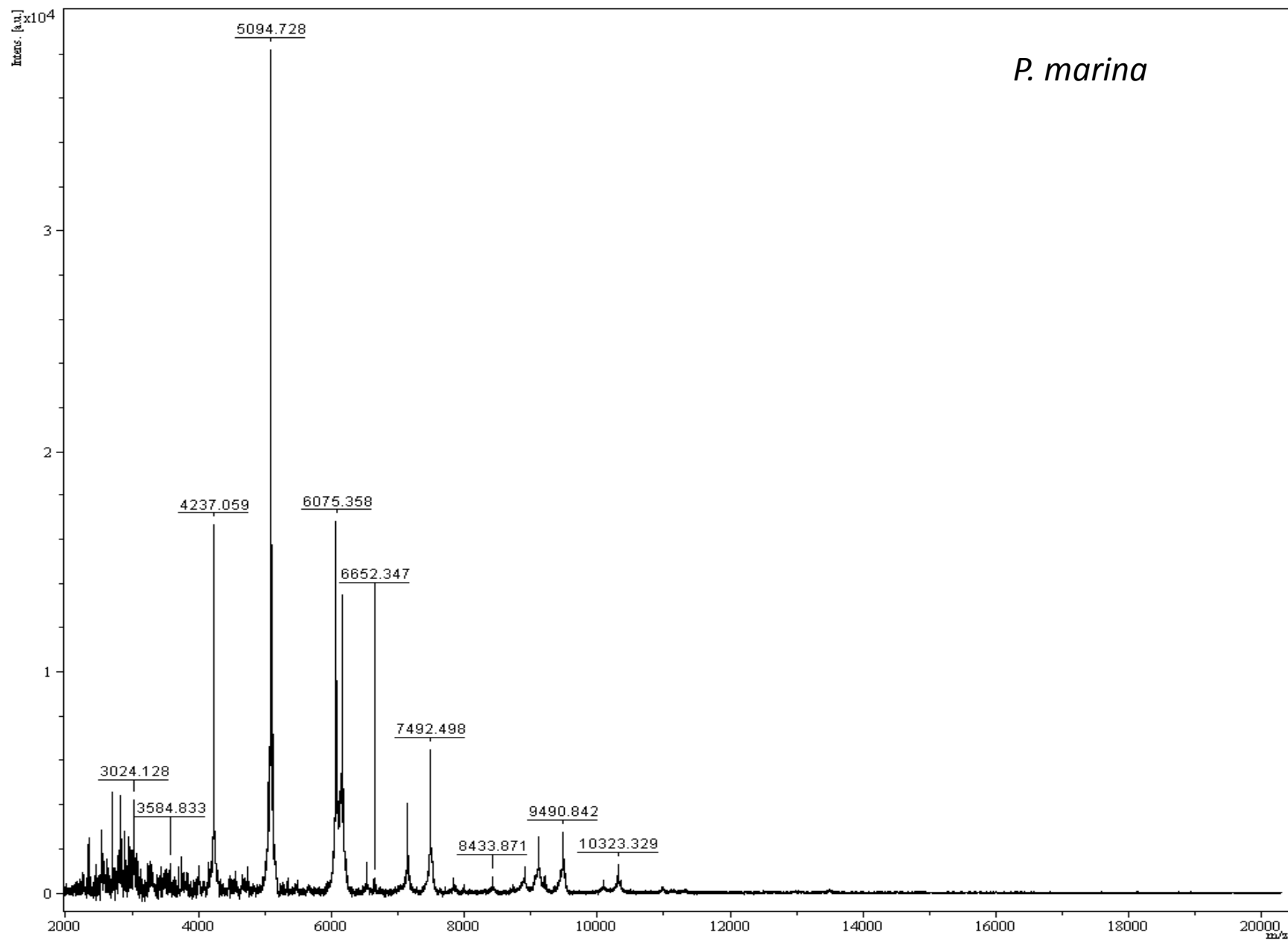

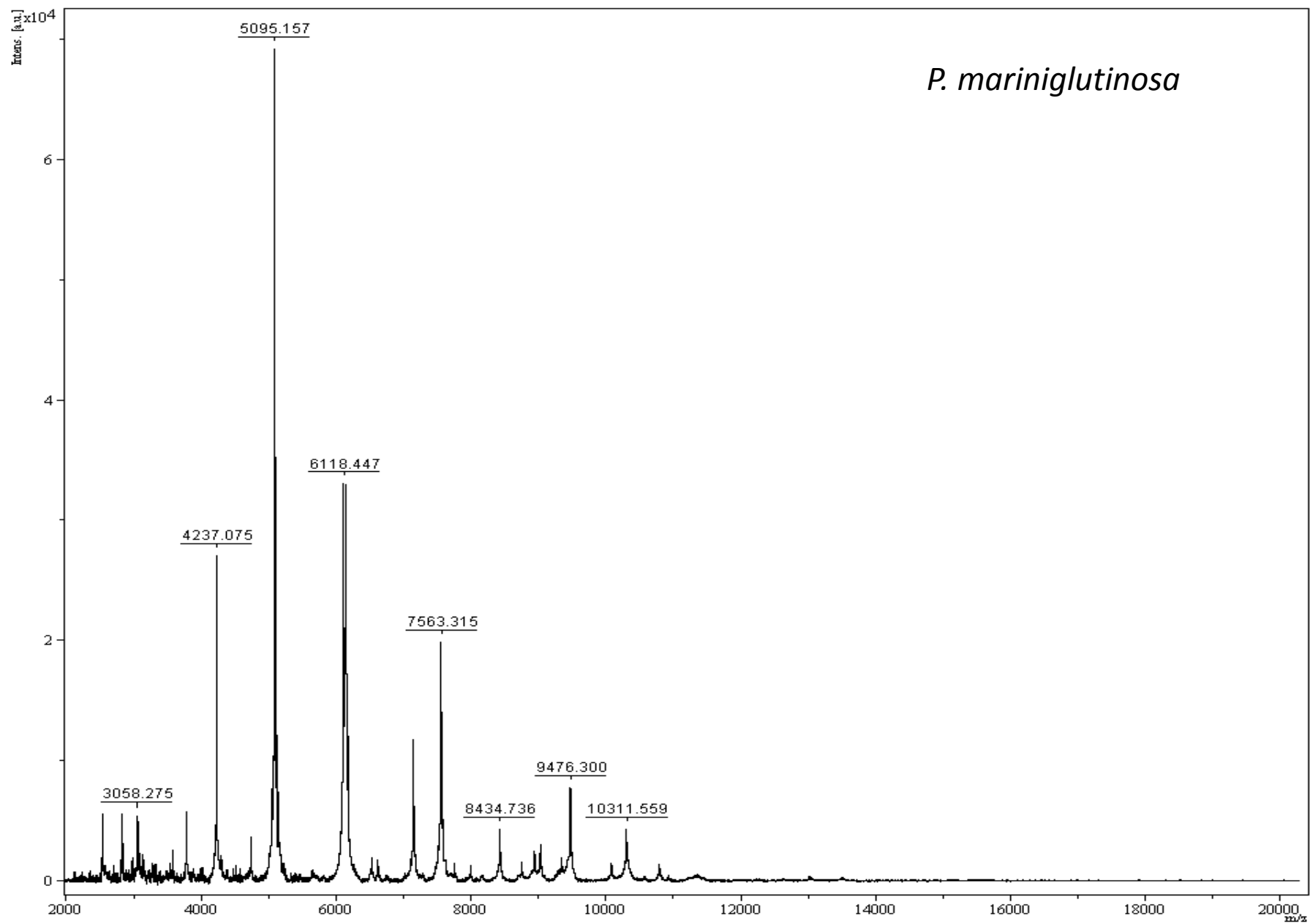

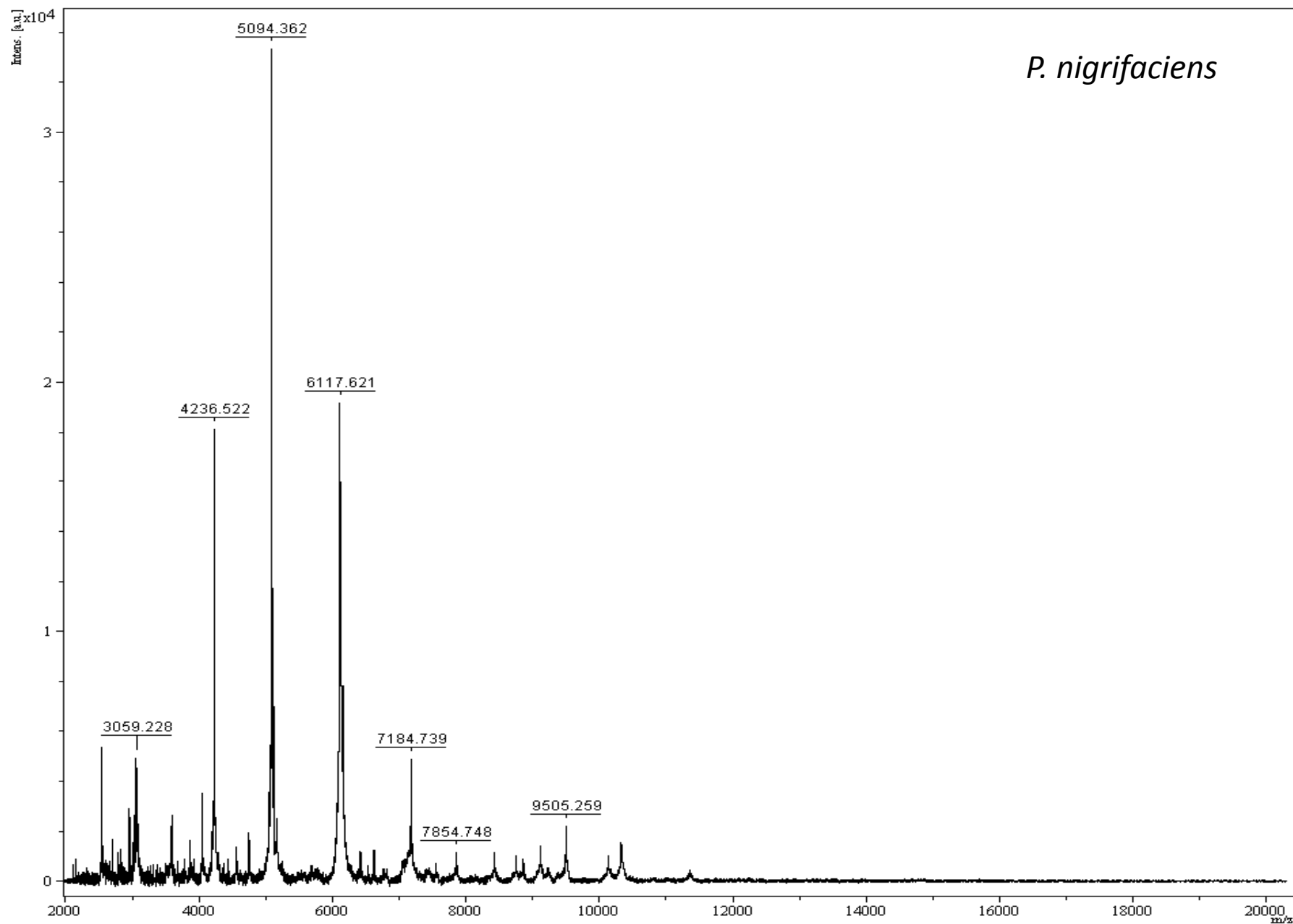

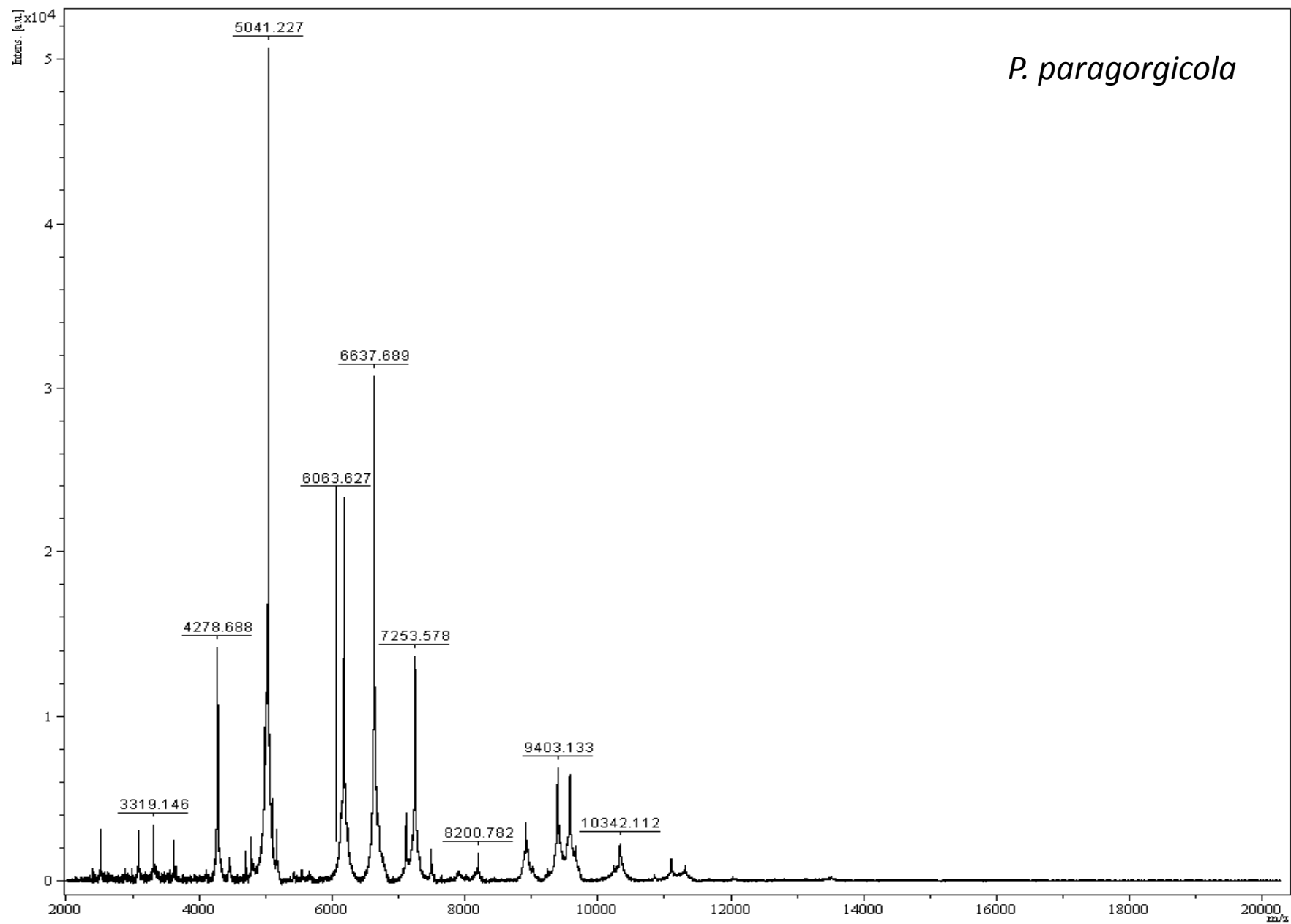

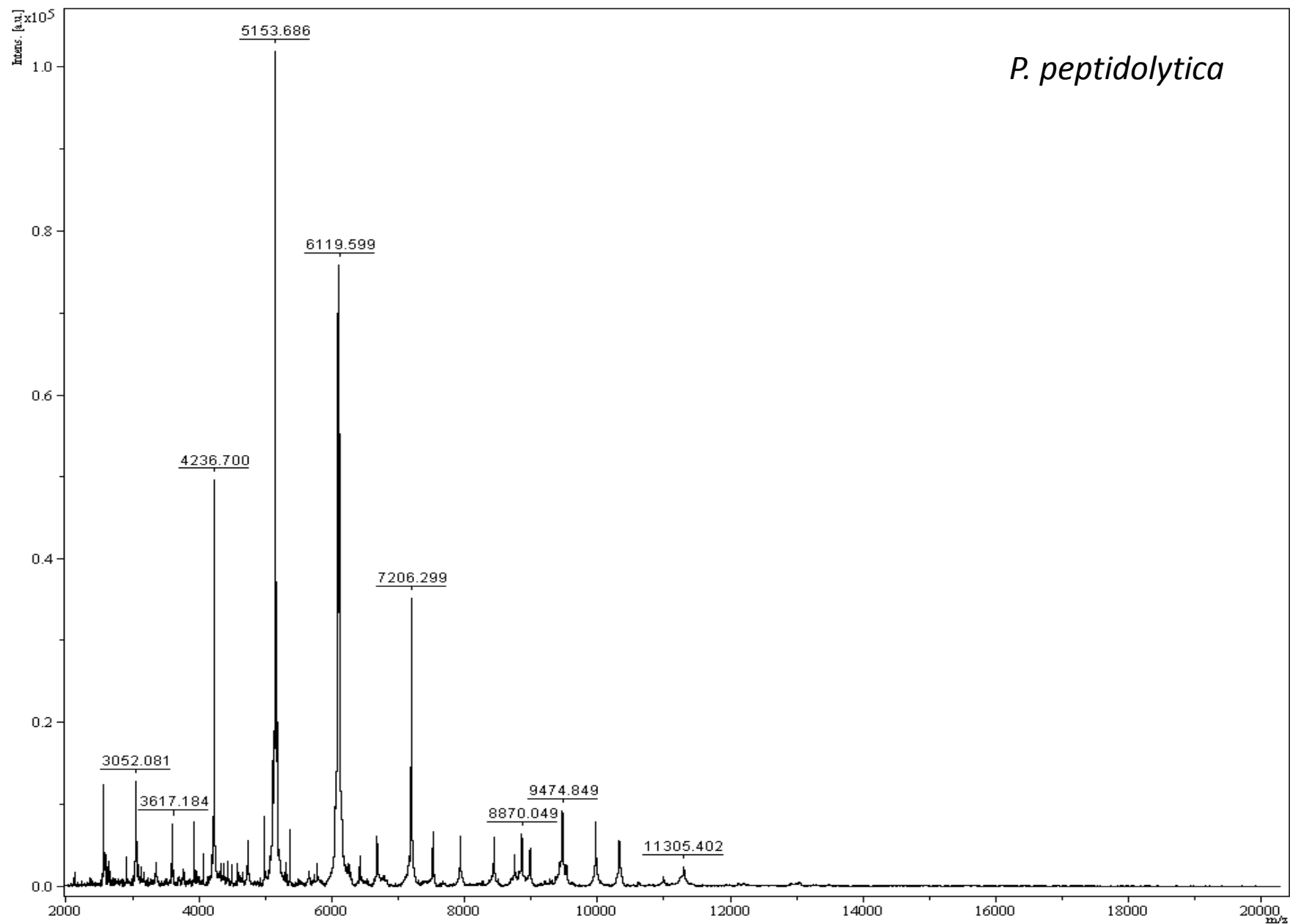

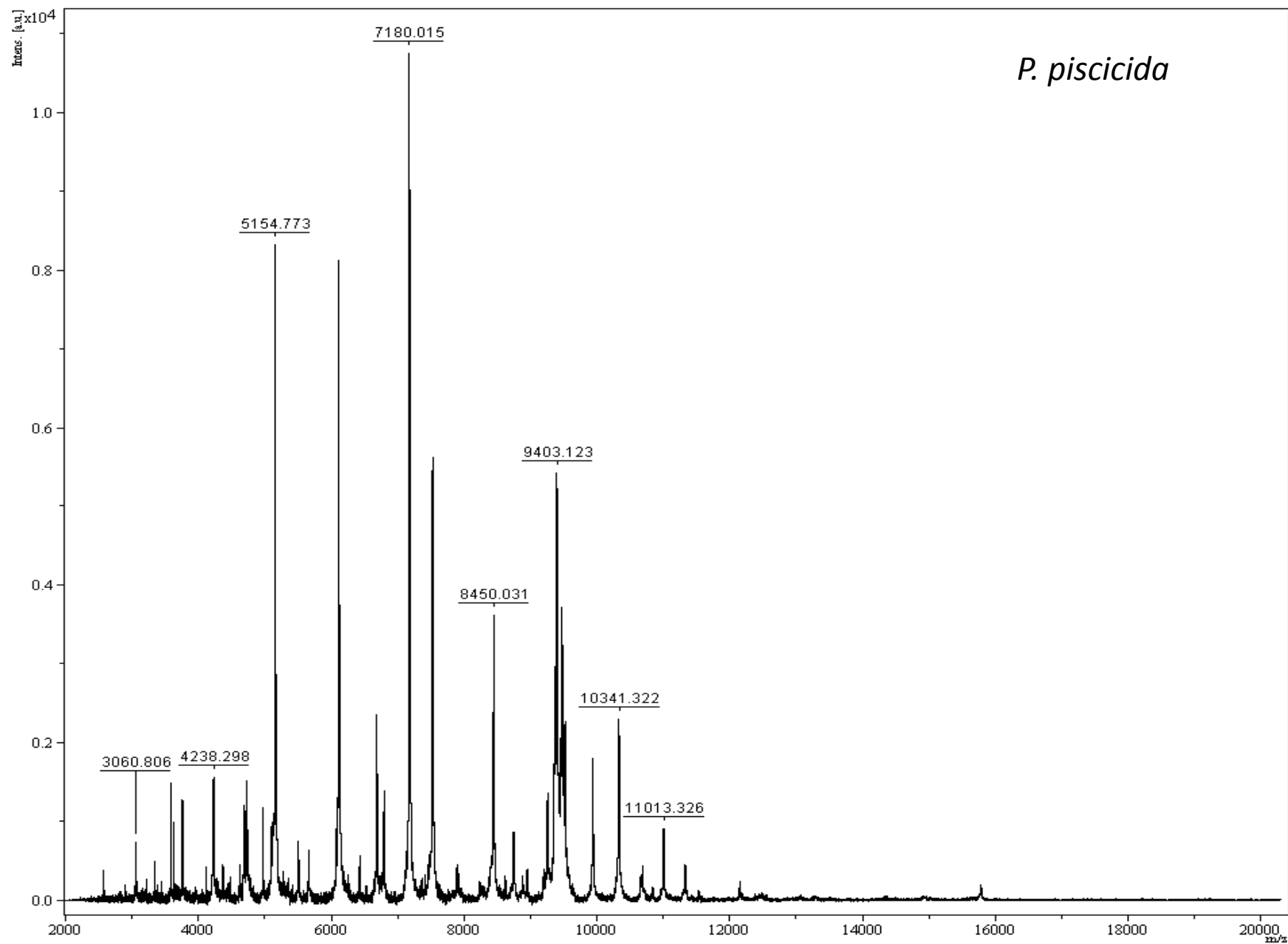

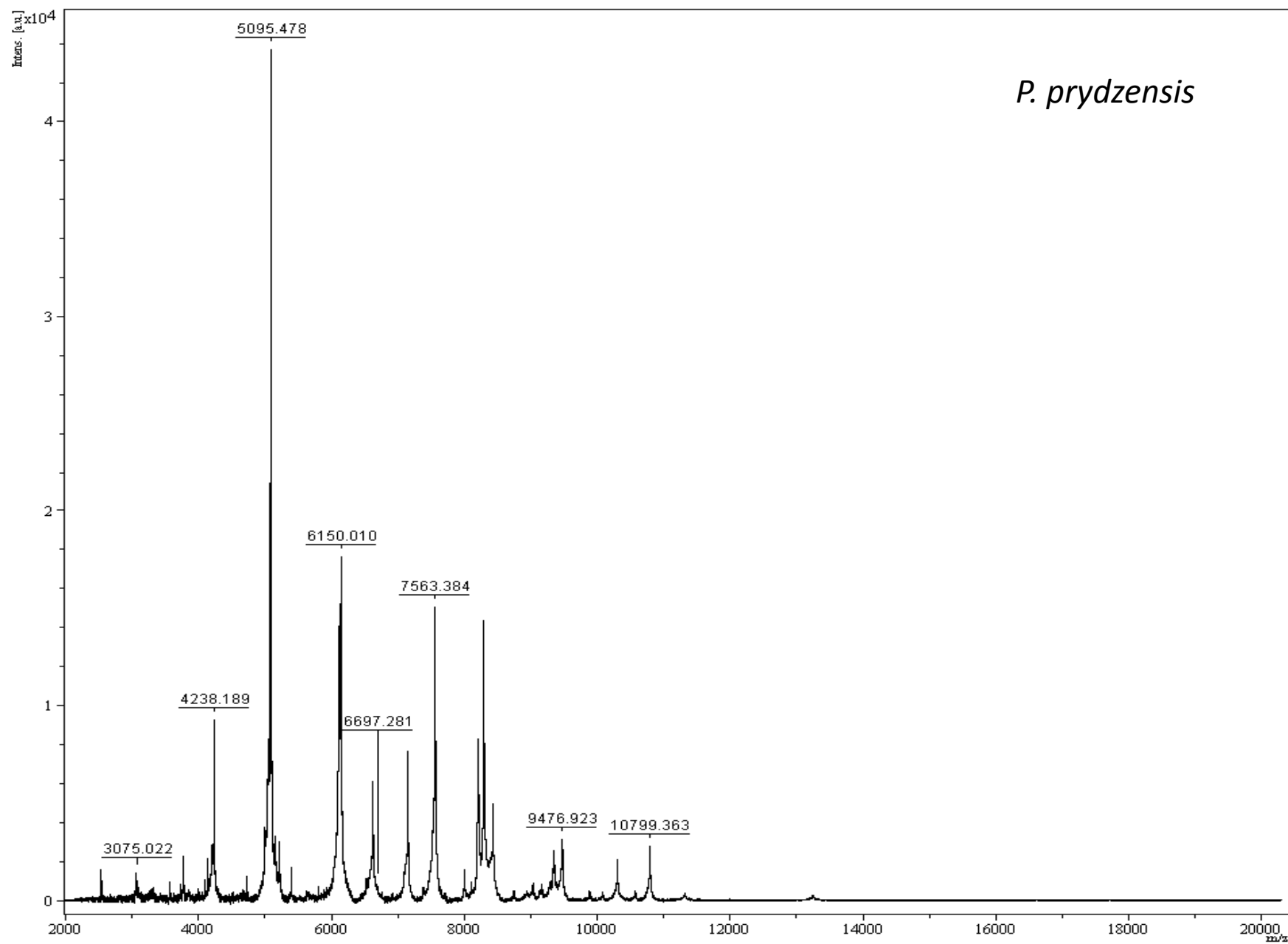

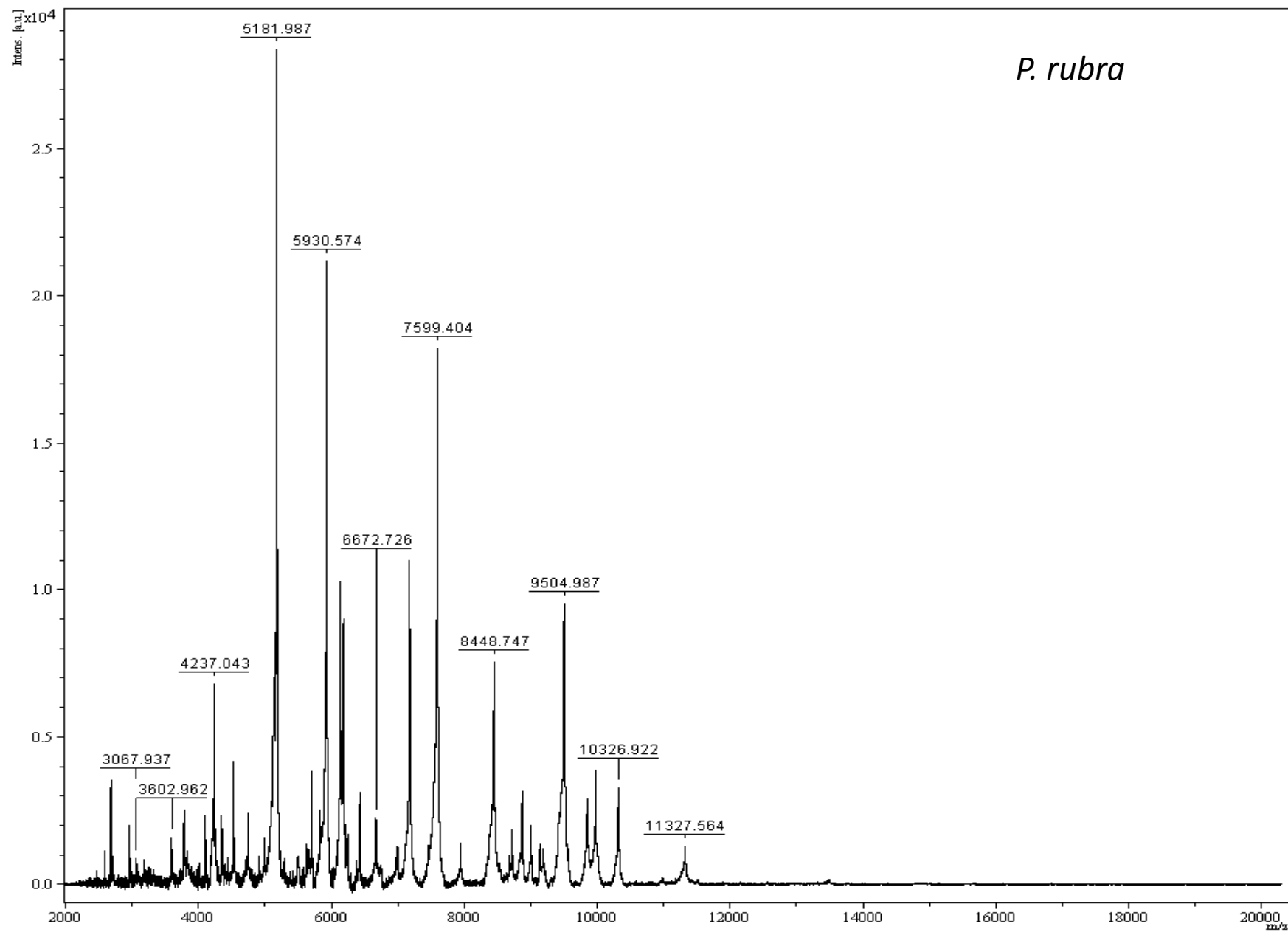

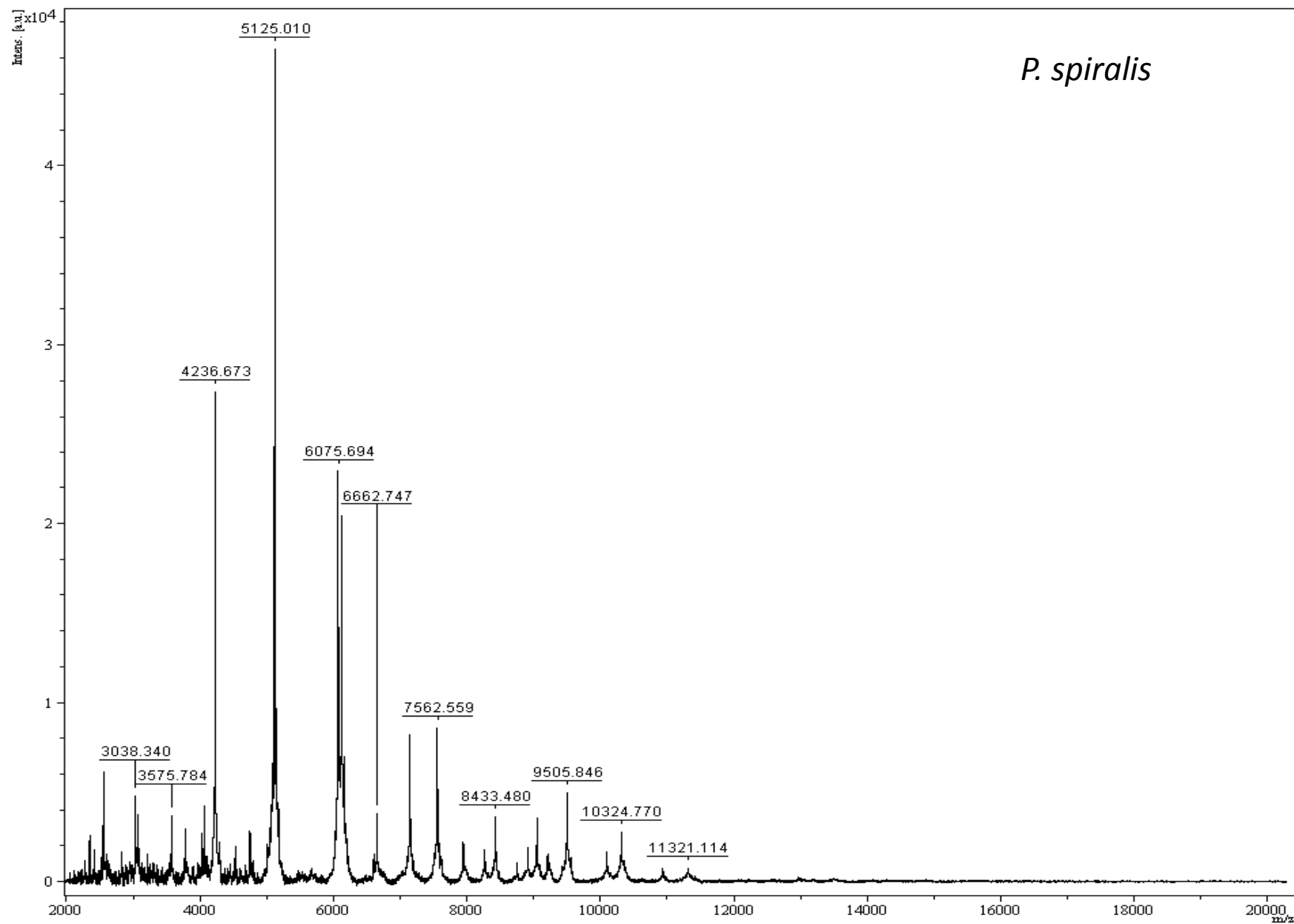

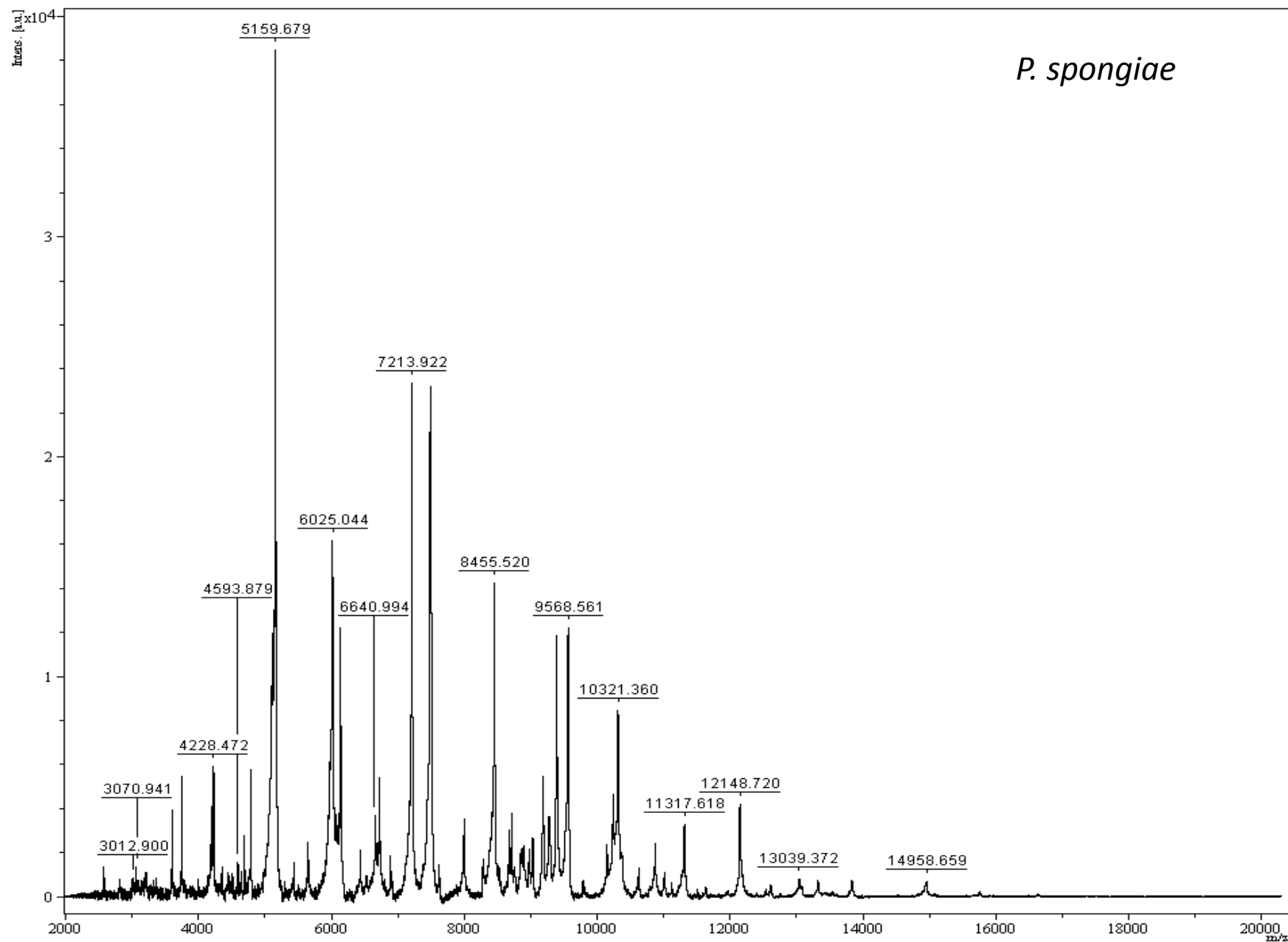

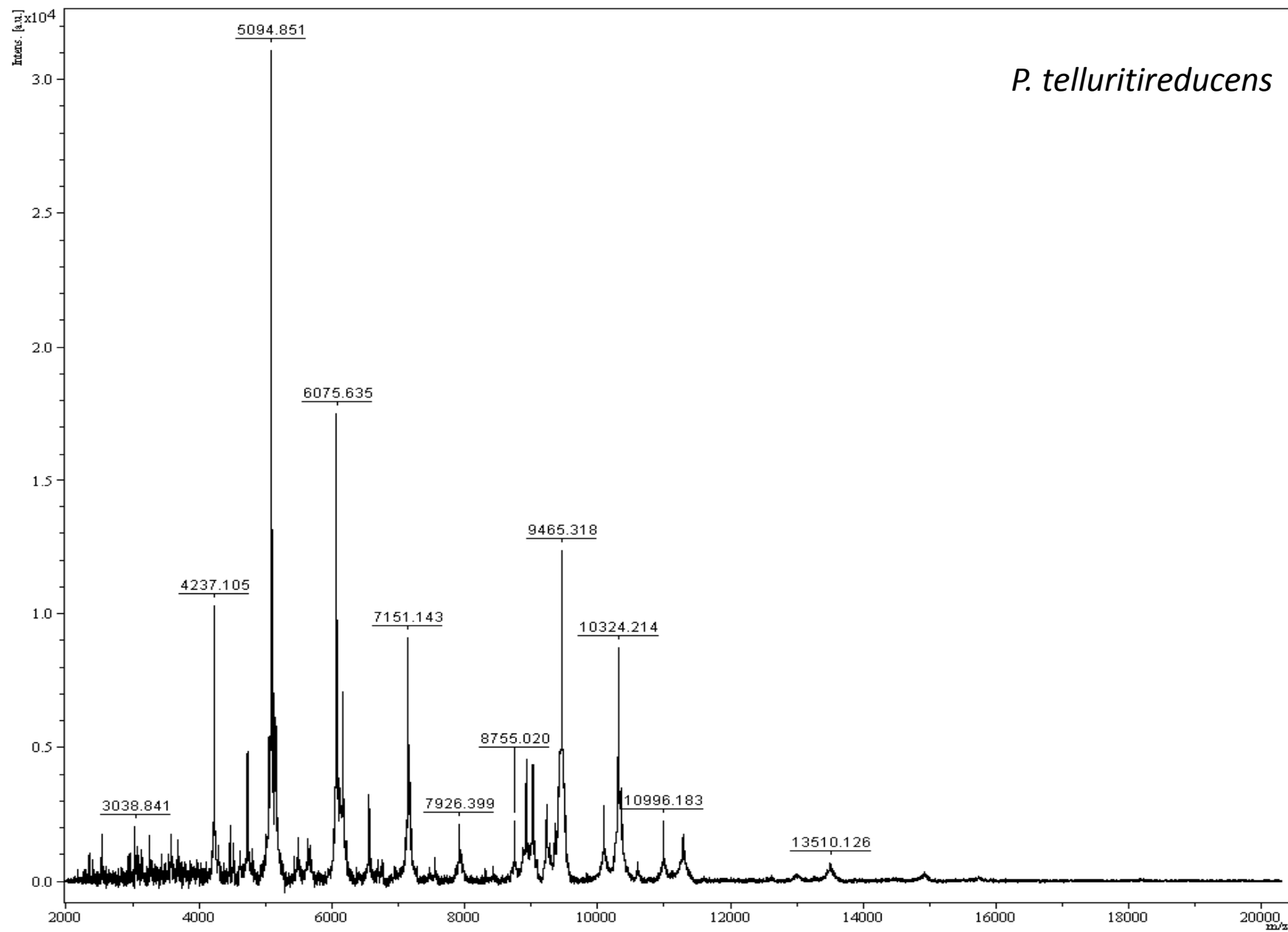

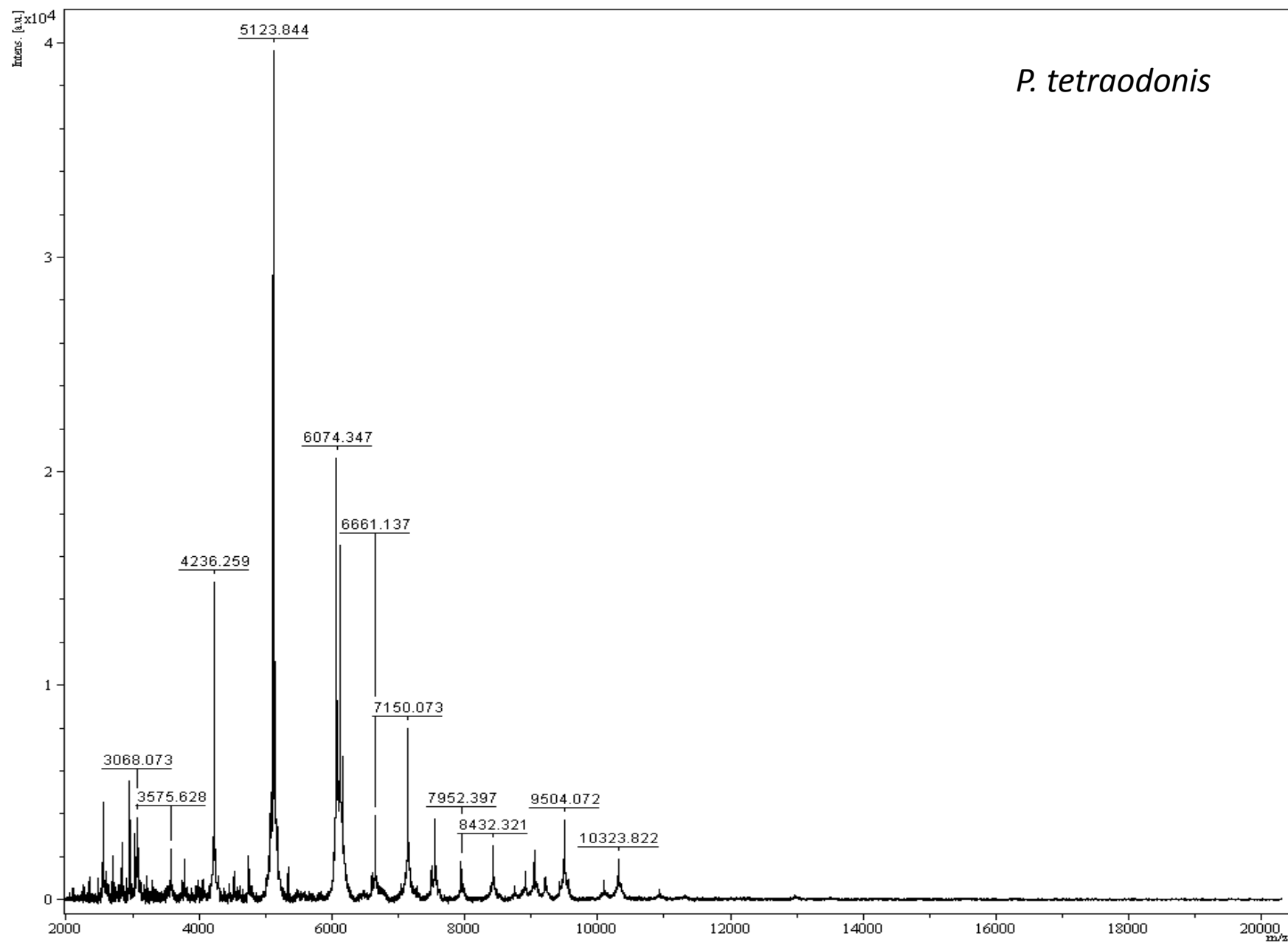

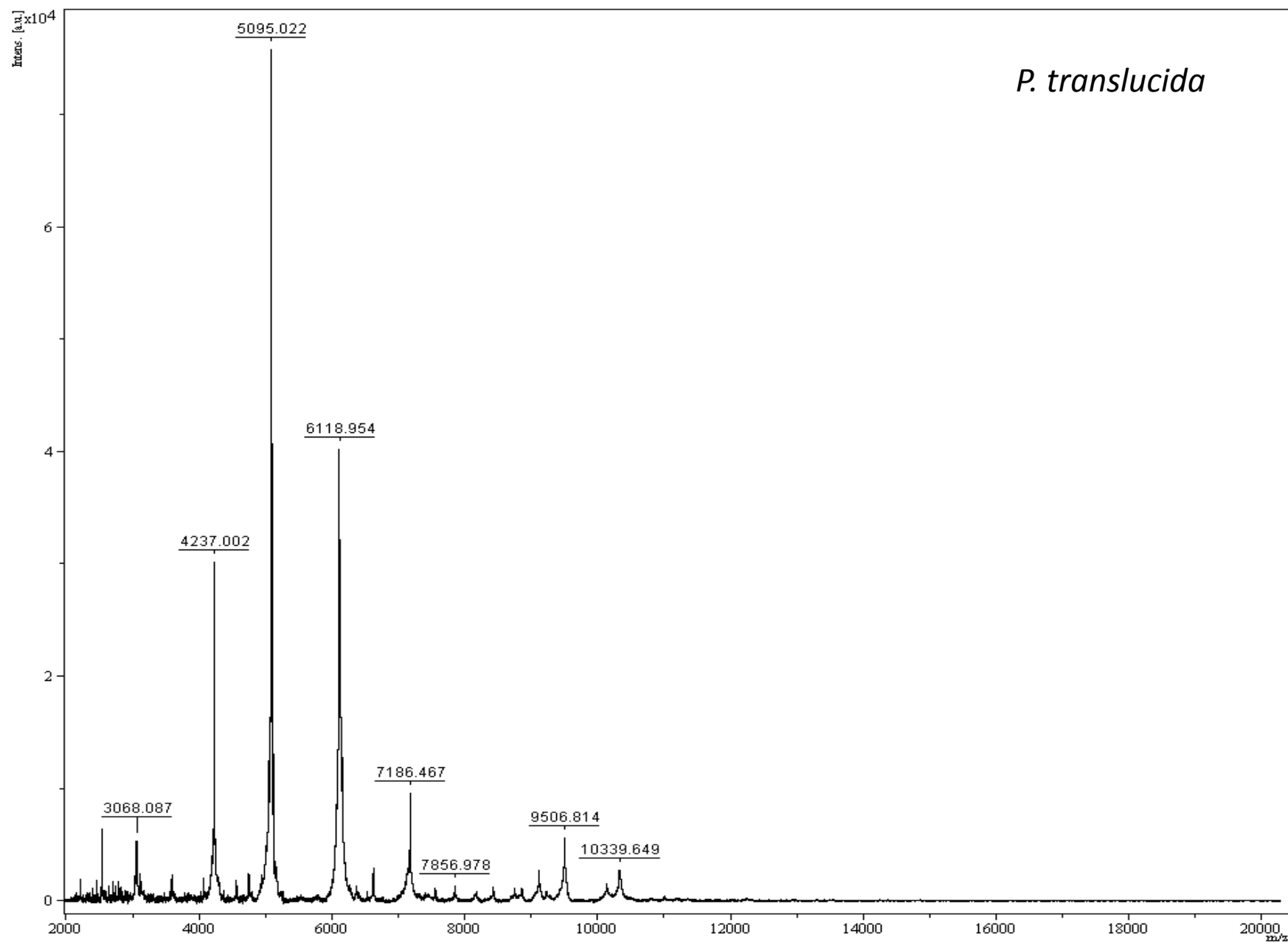

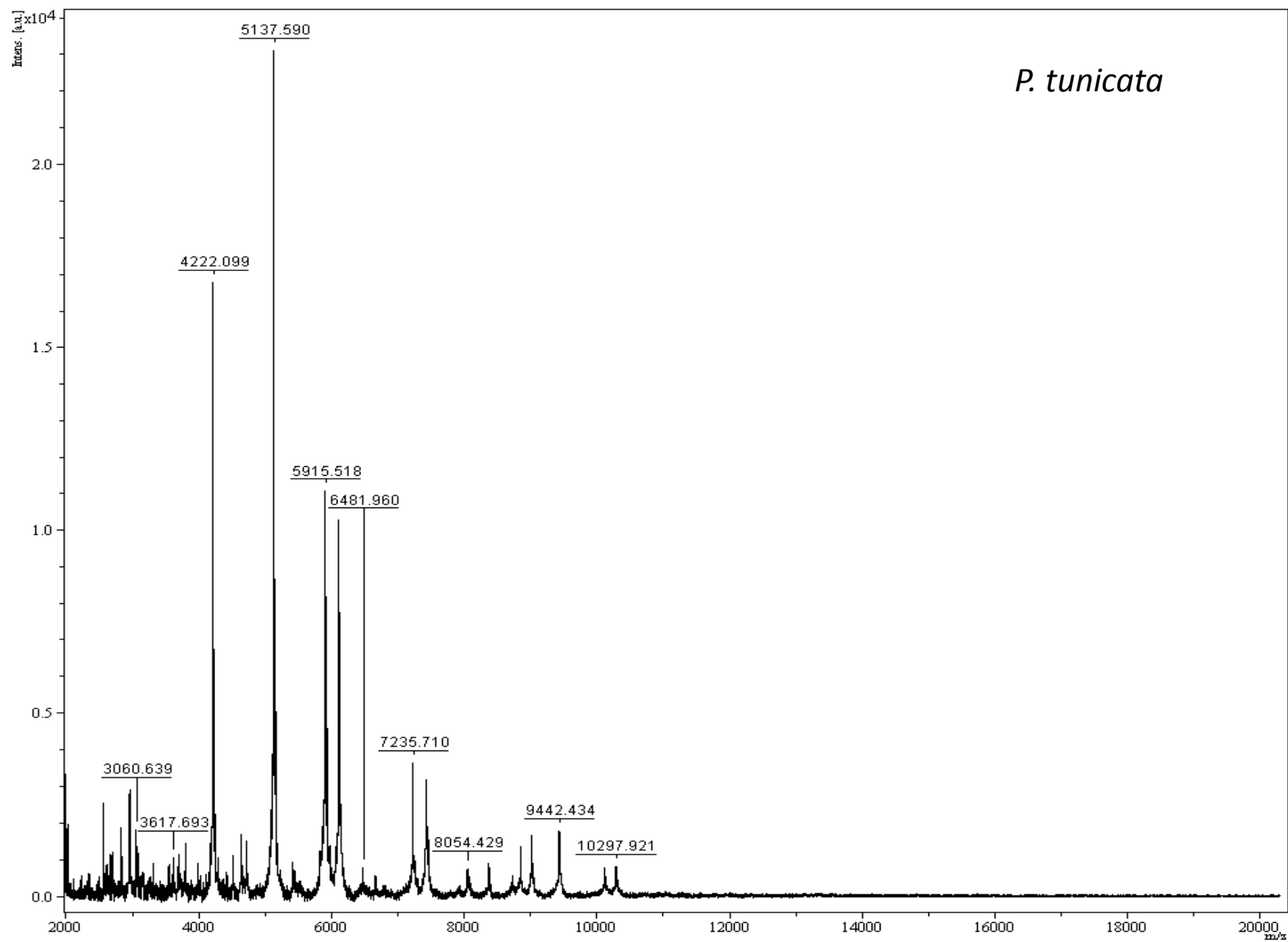

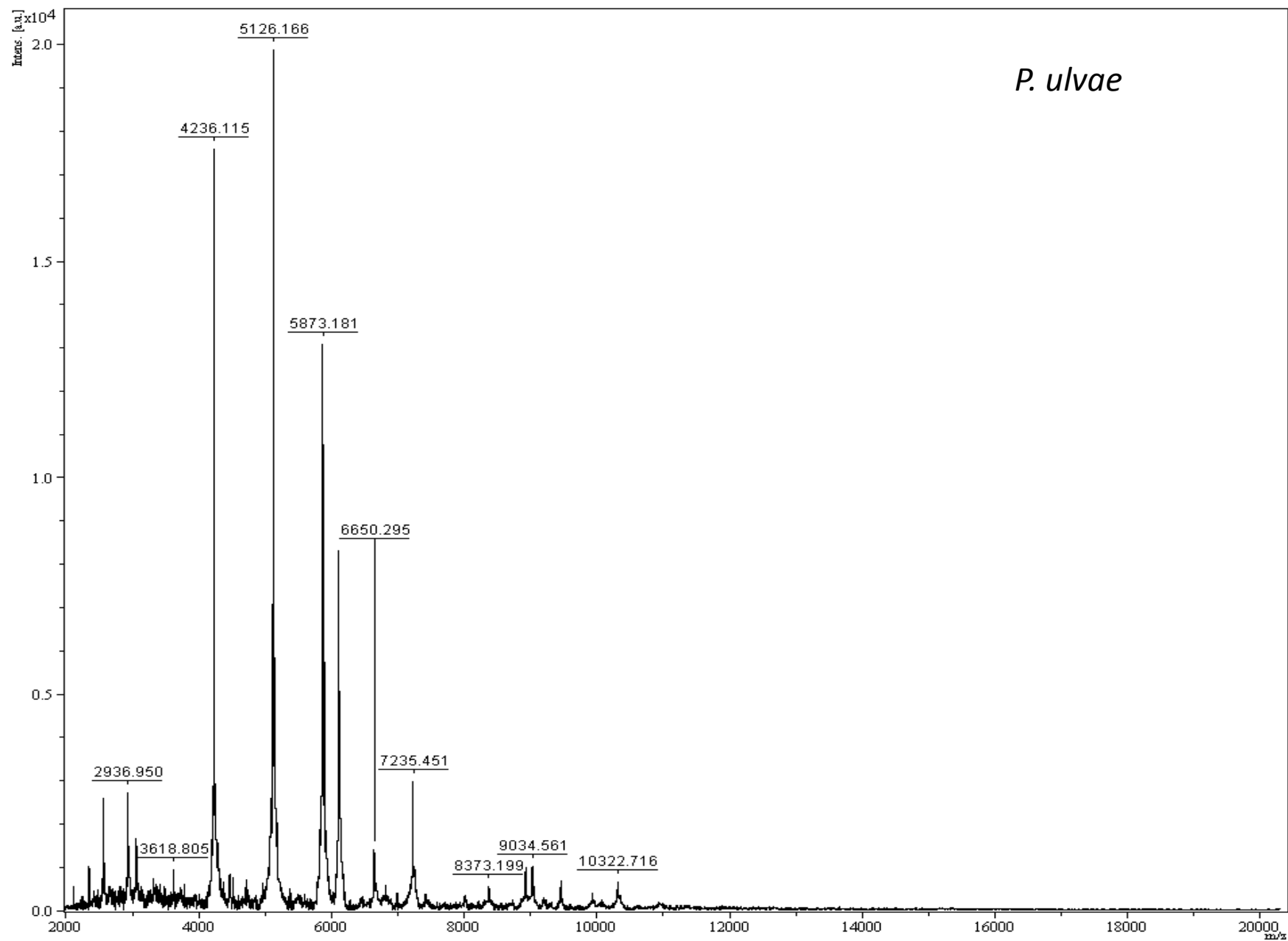

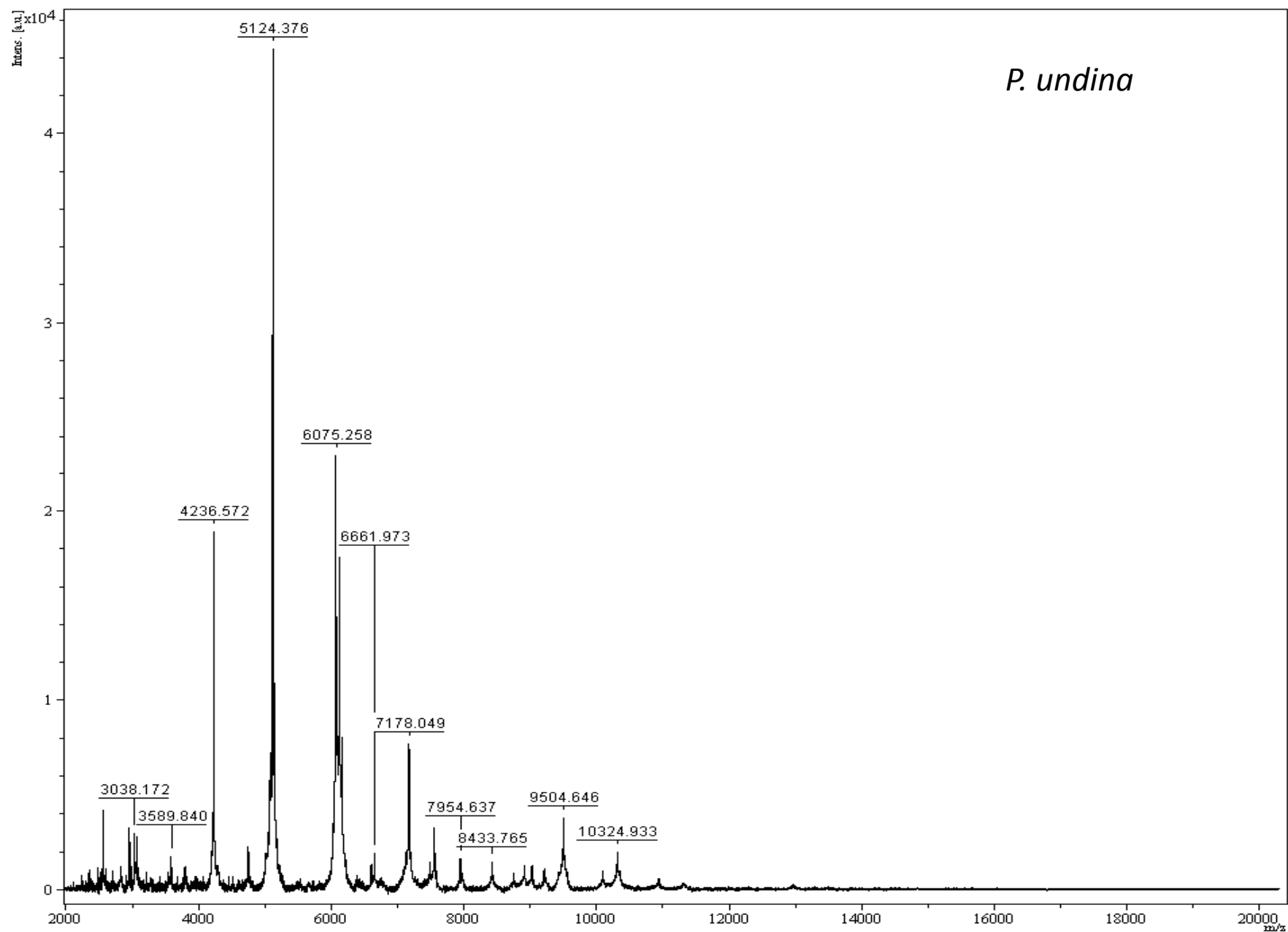

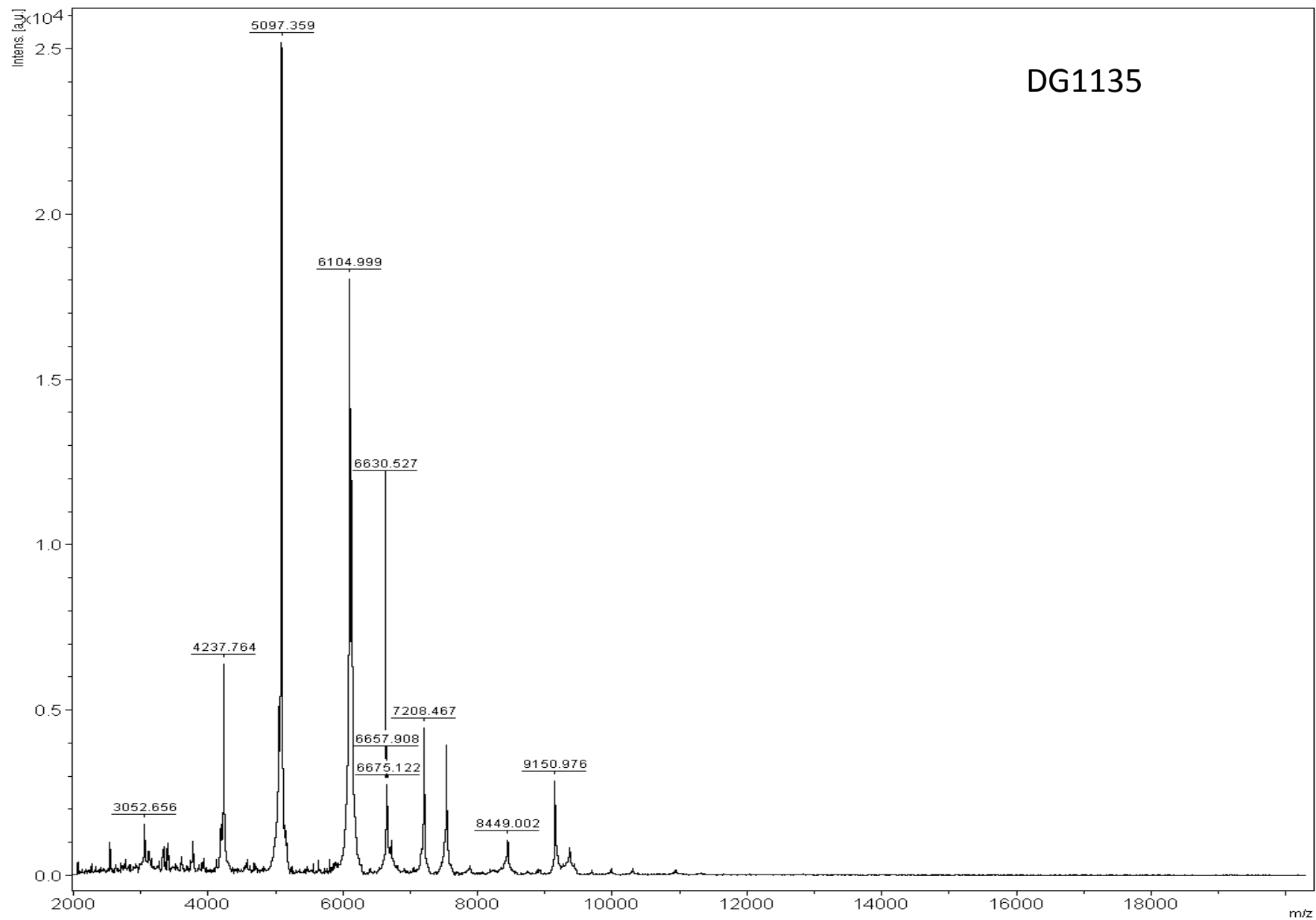

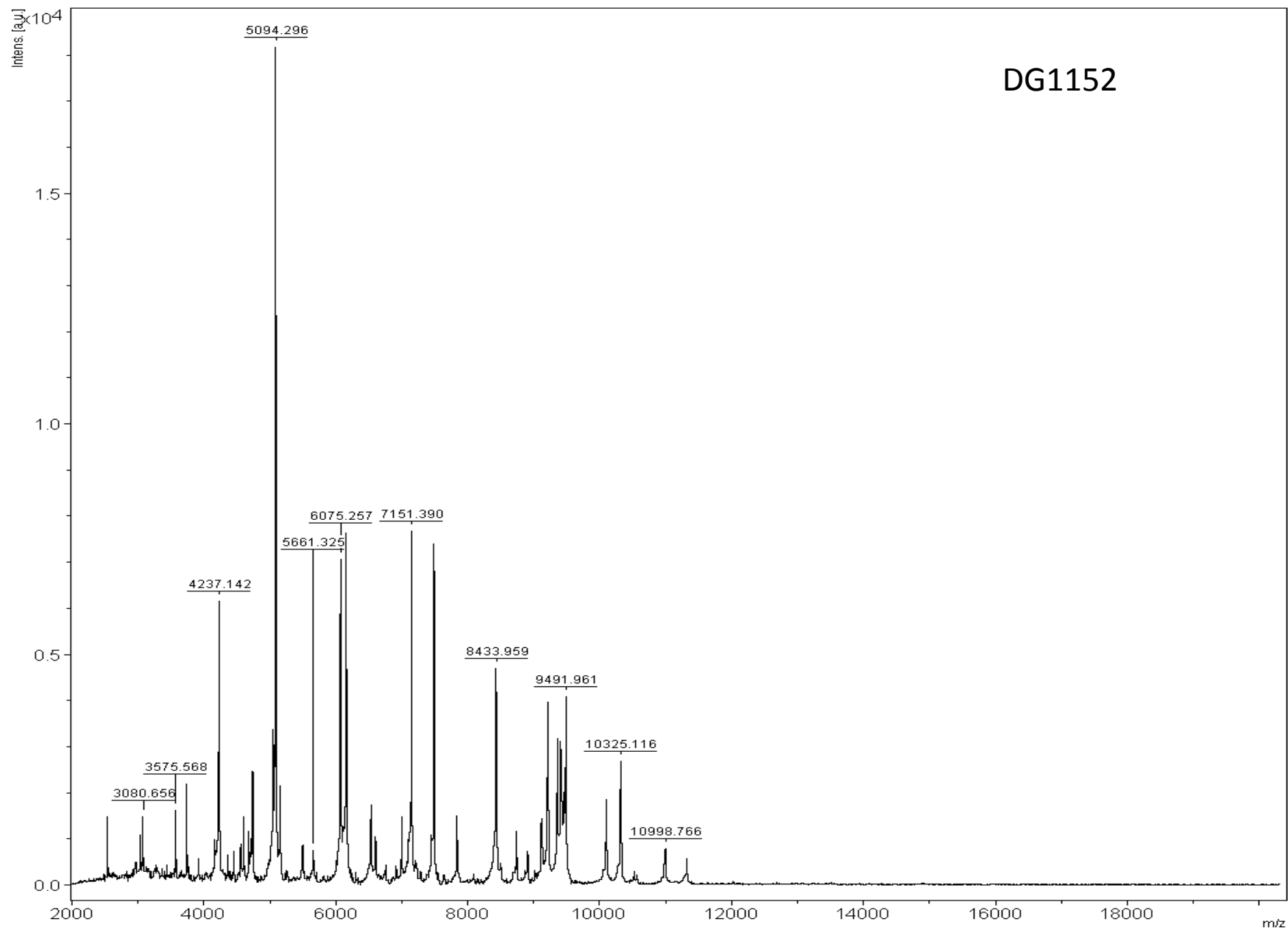

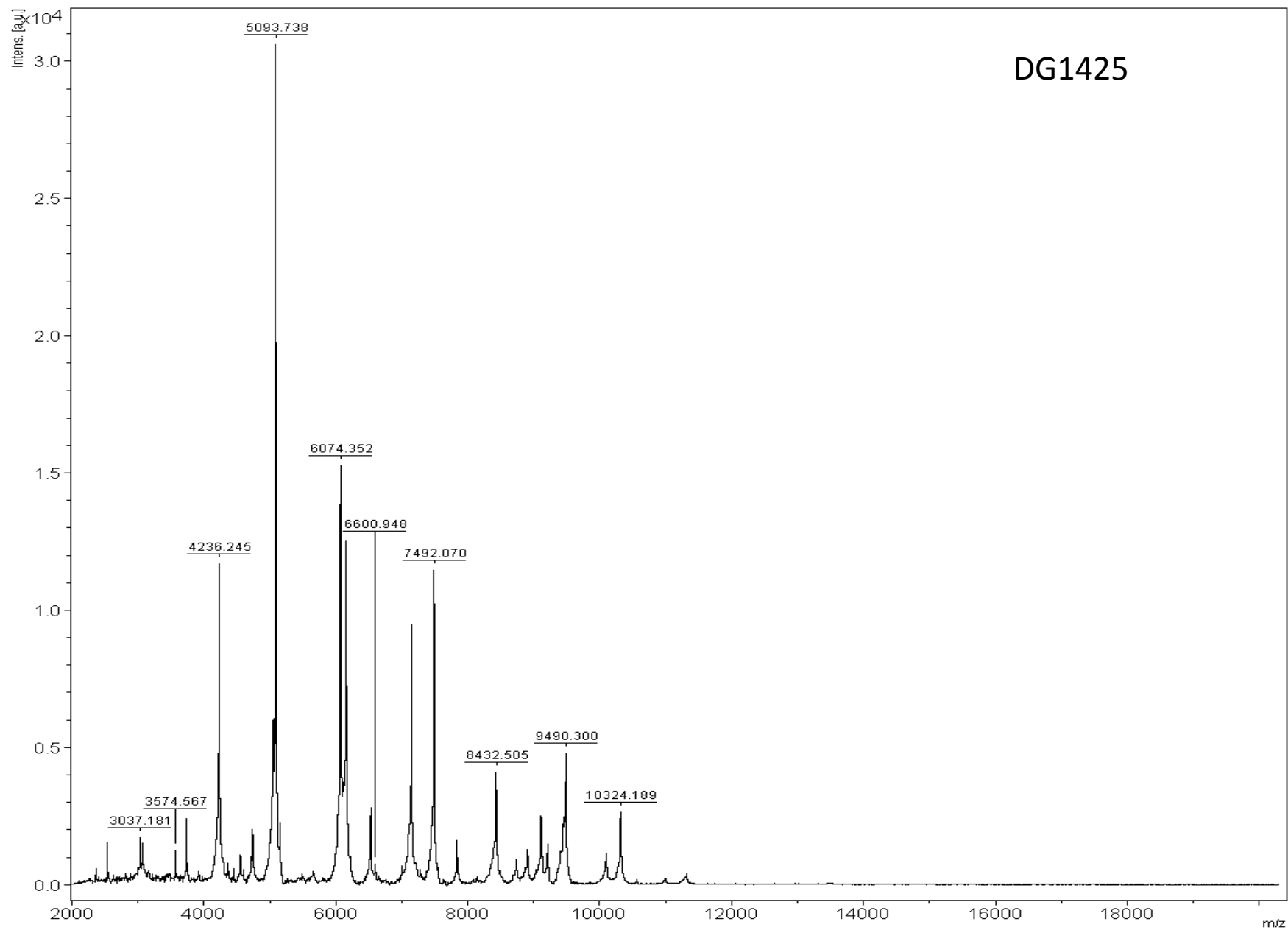

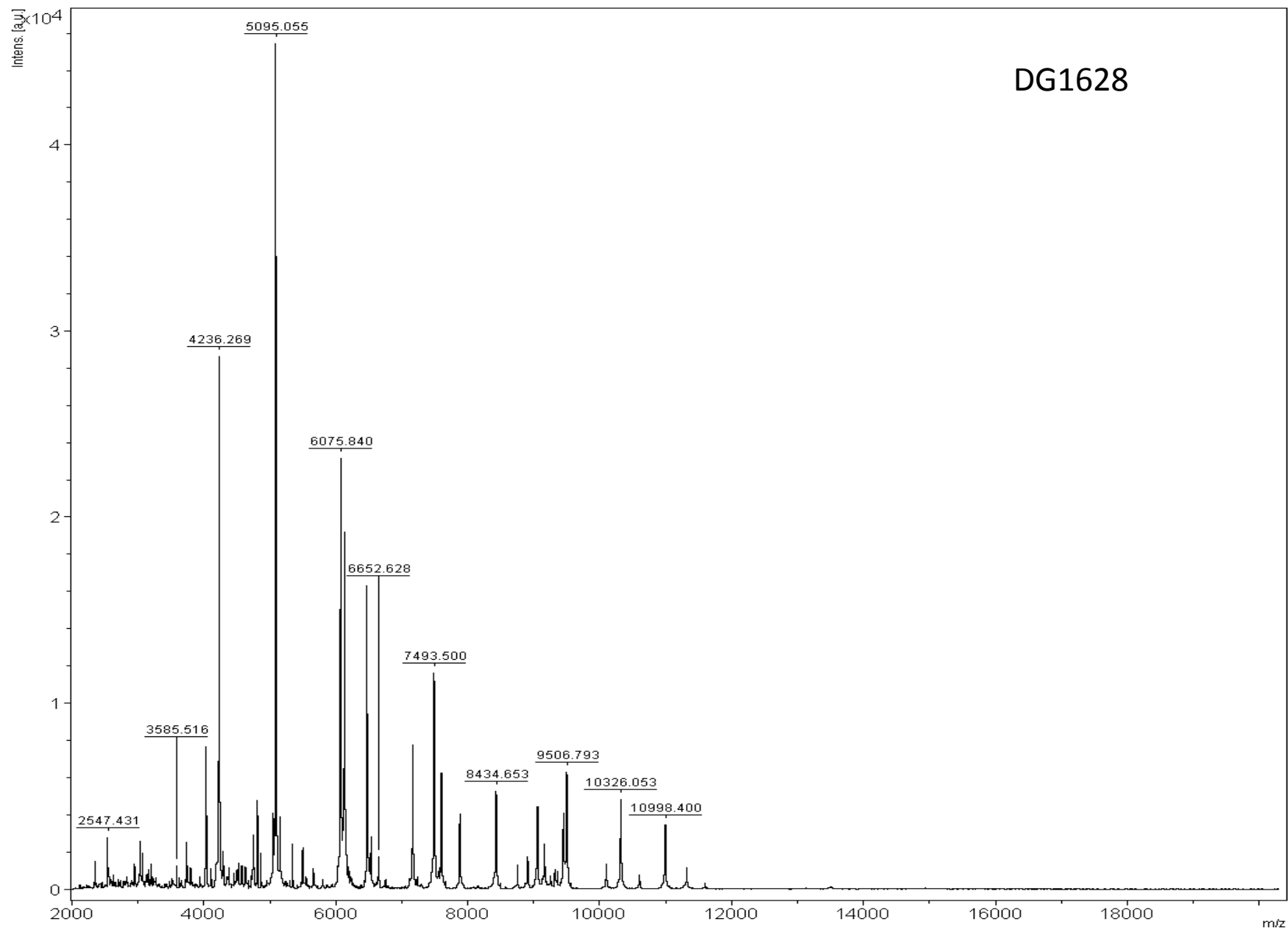

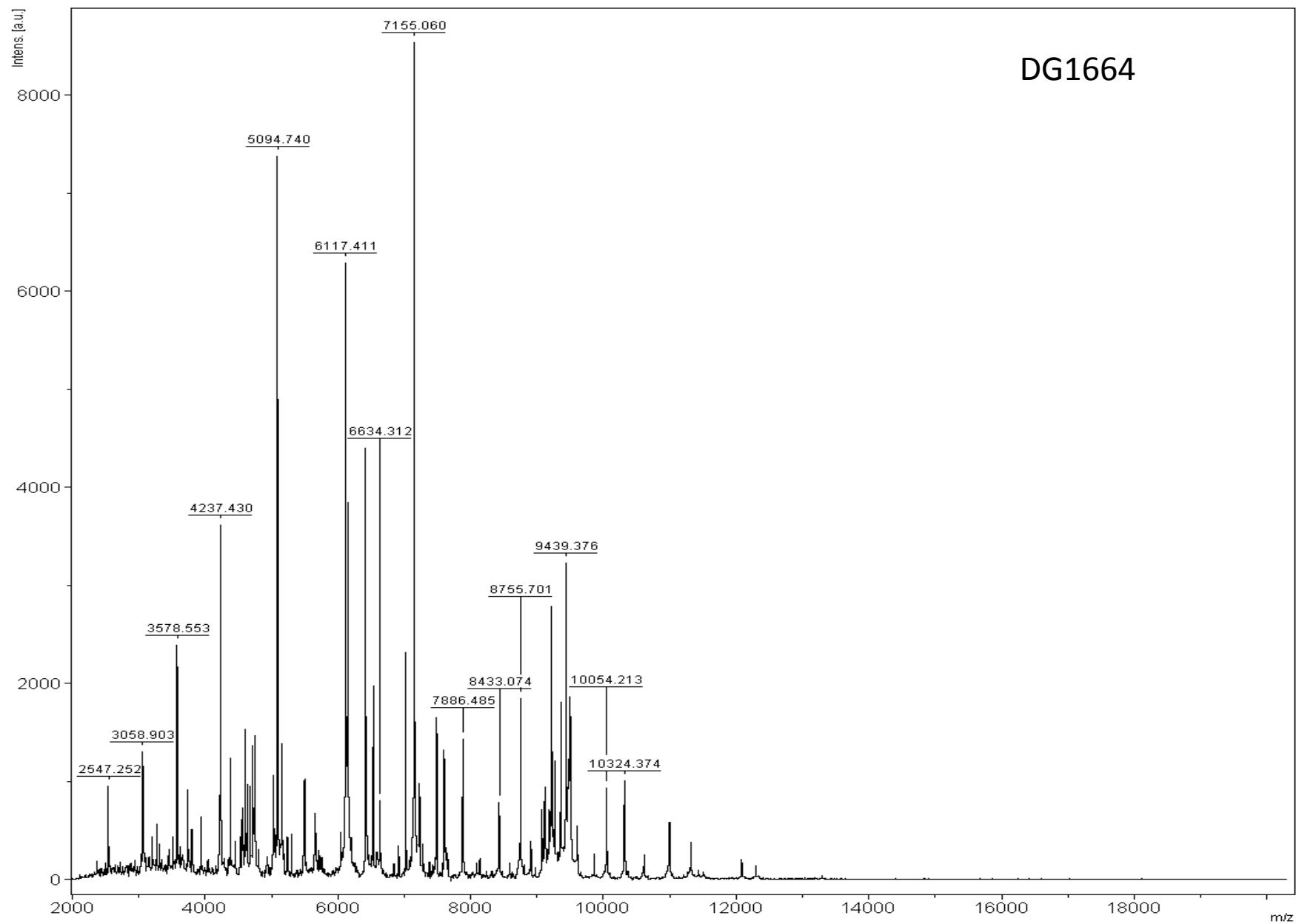

DG1664

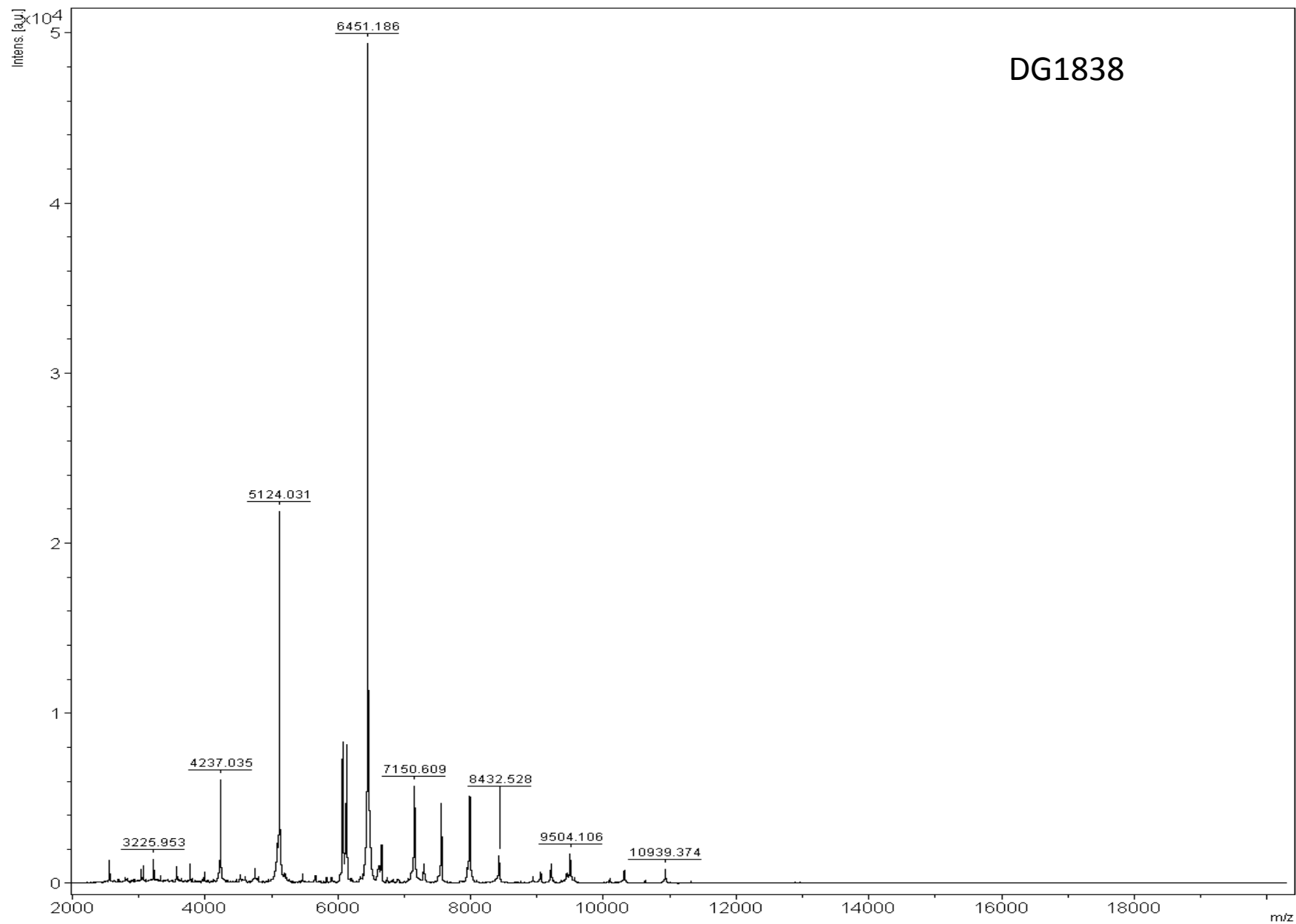

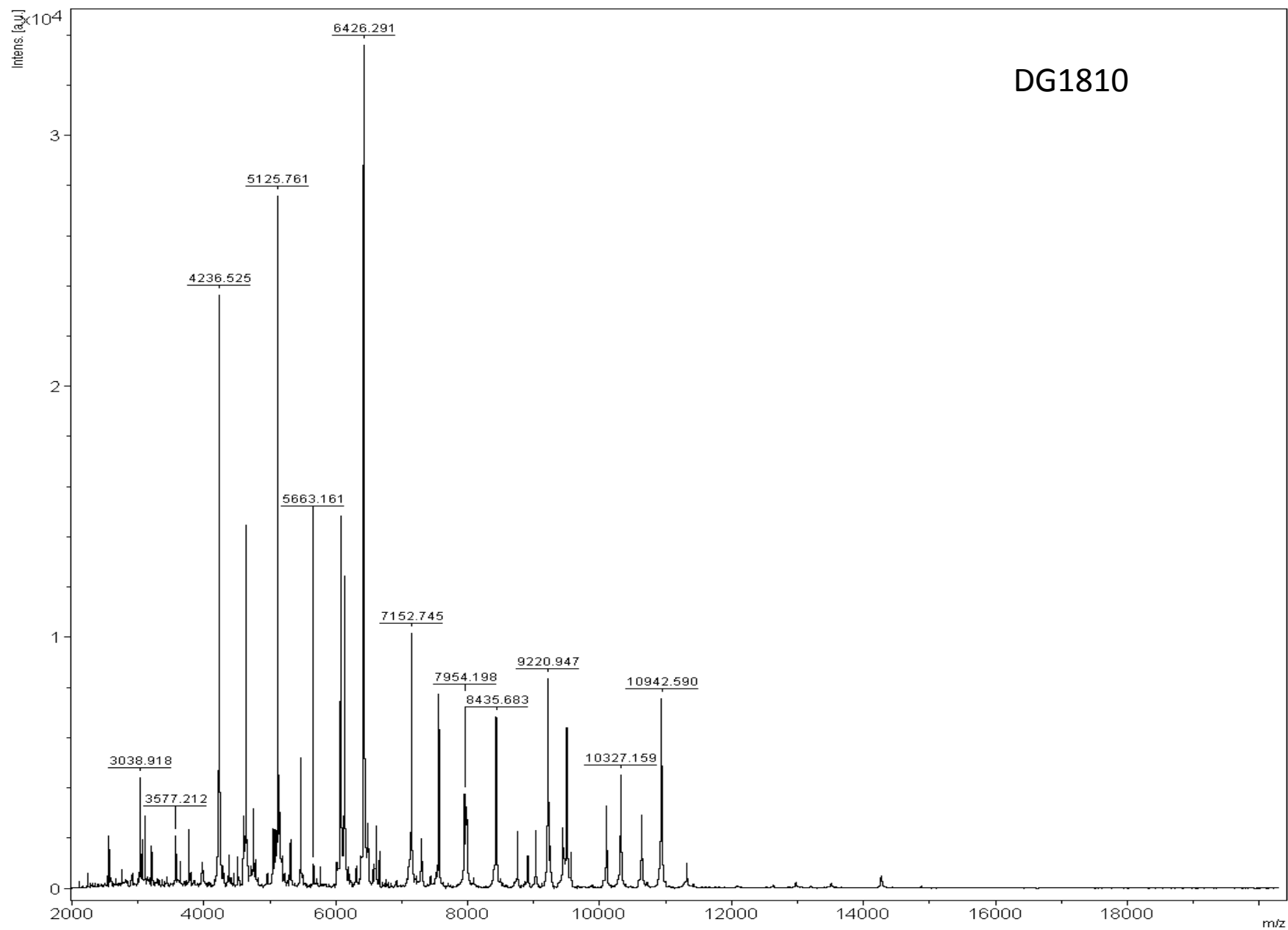

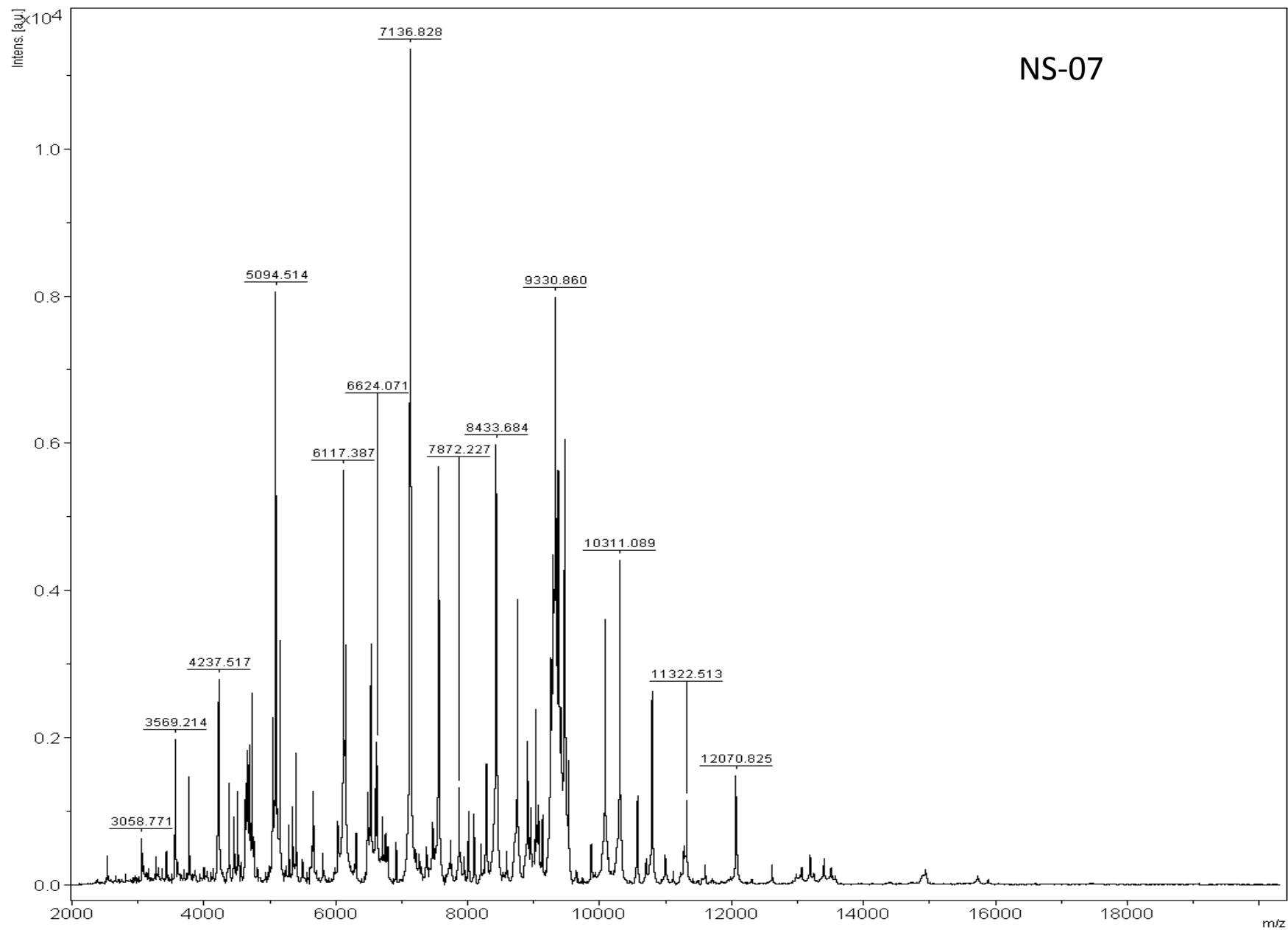

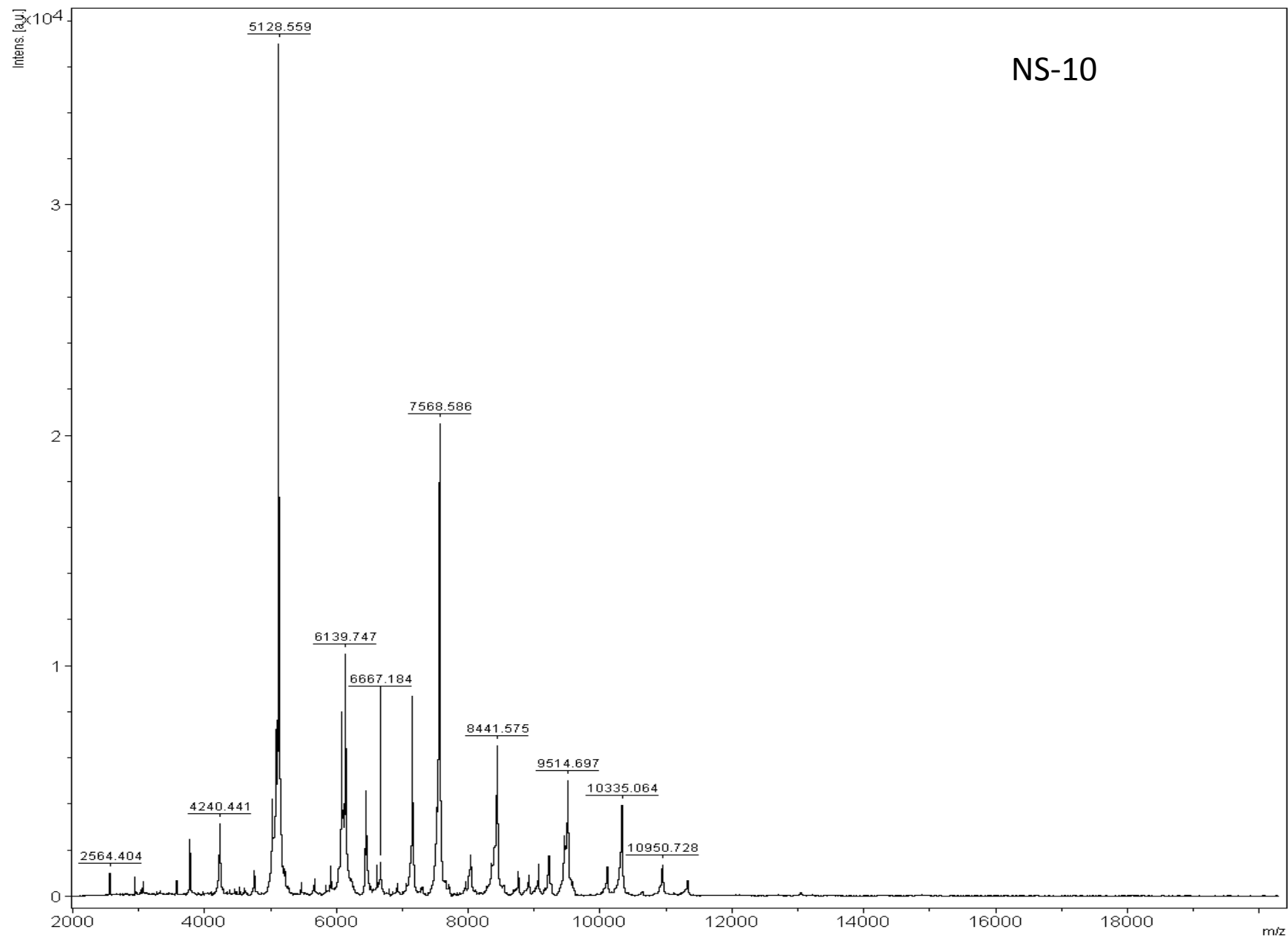

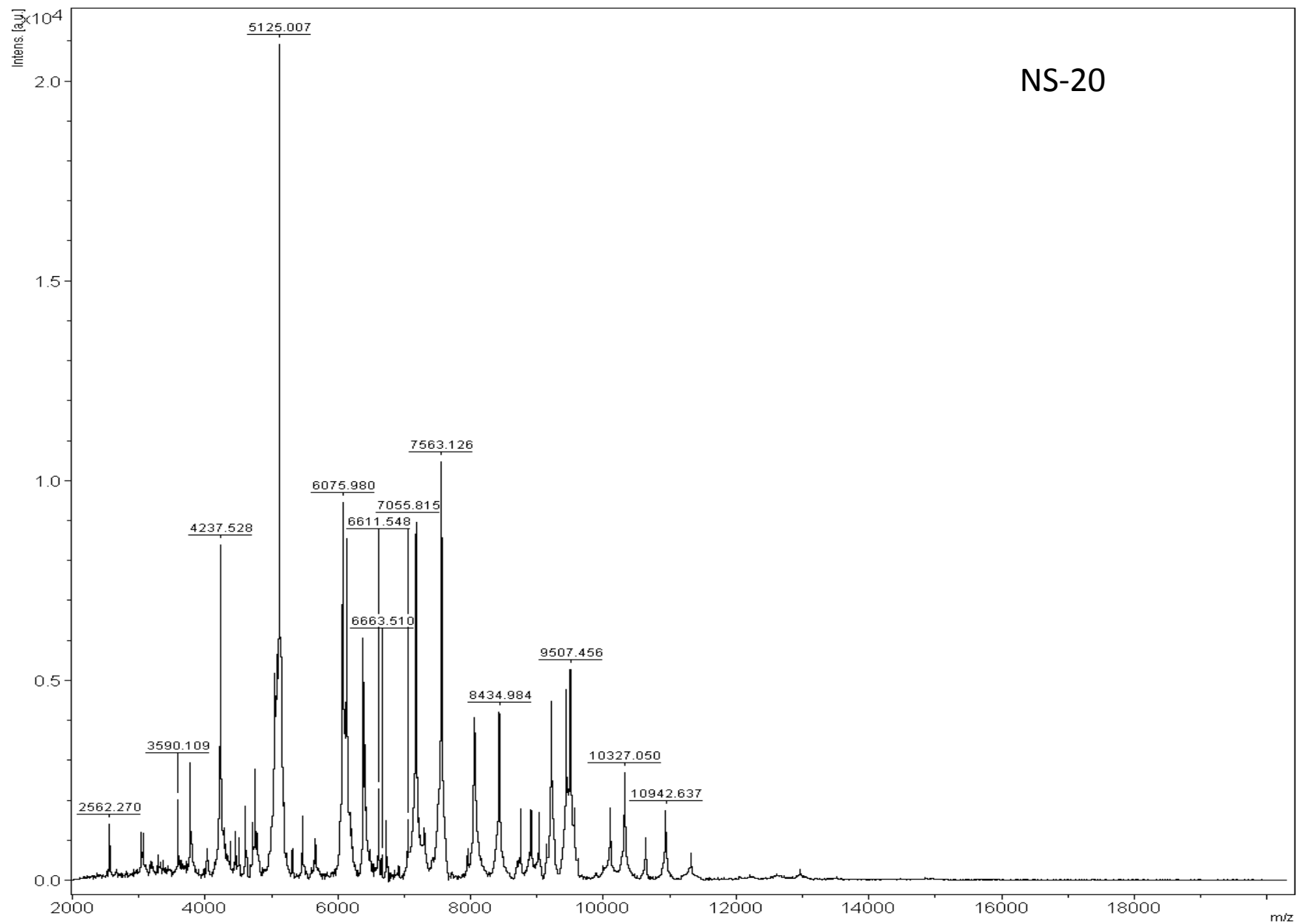

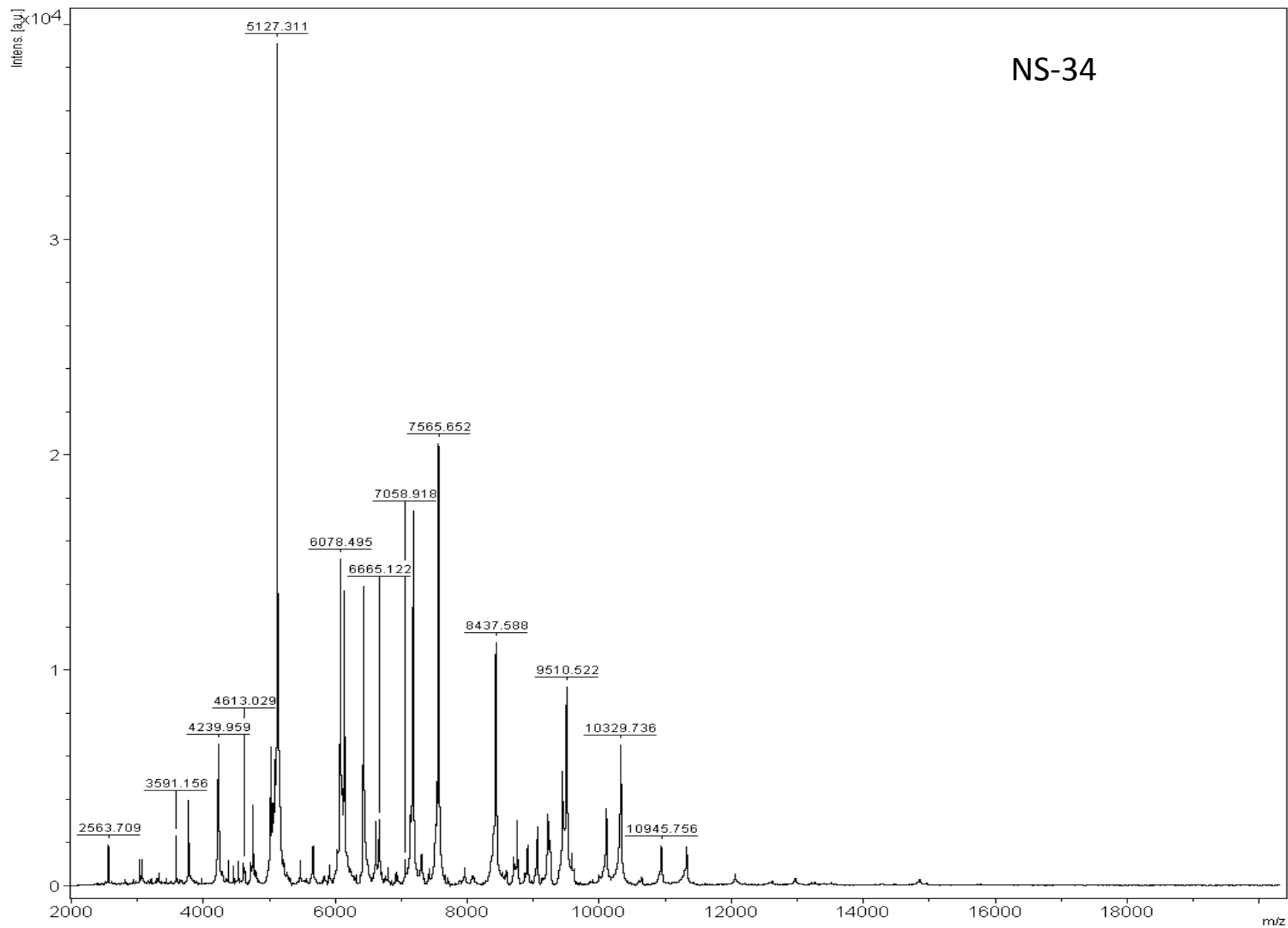

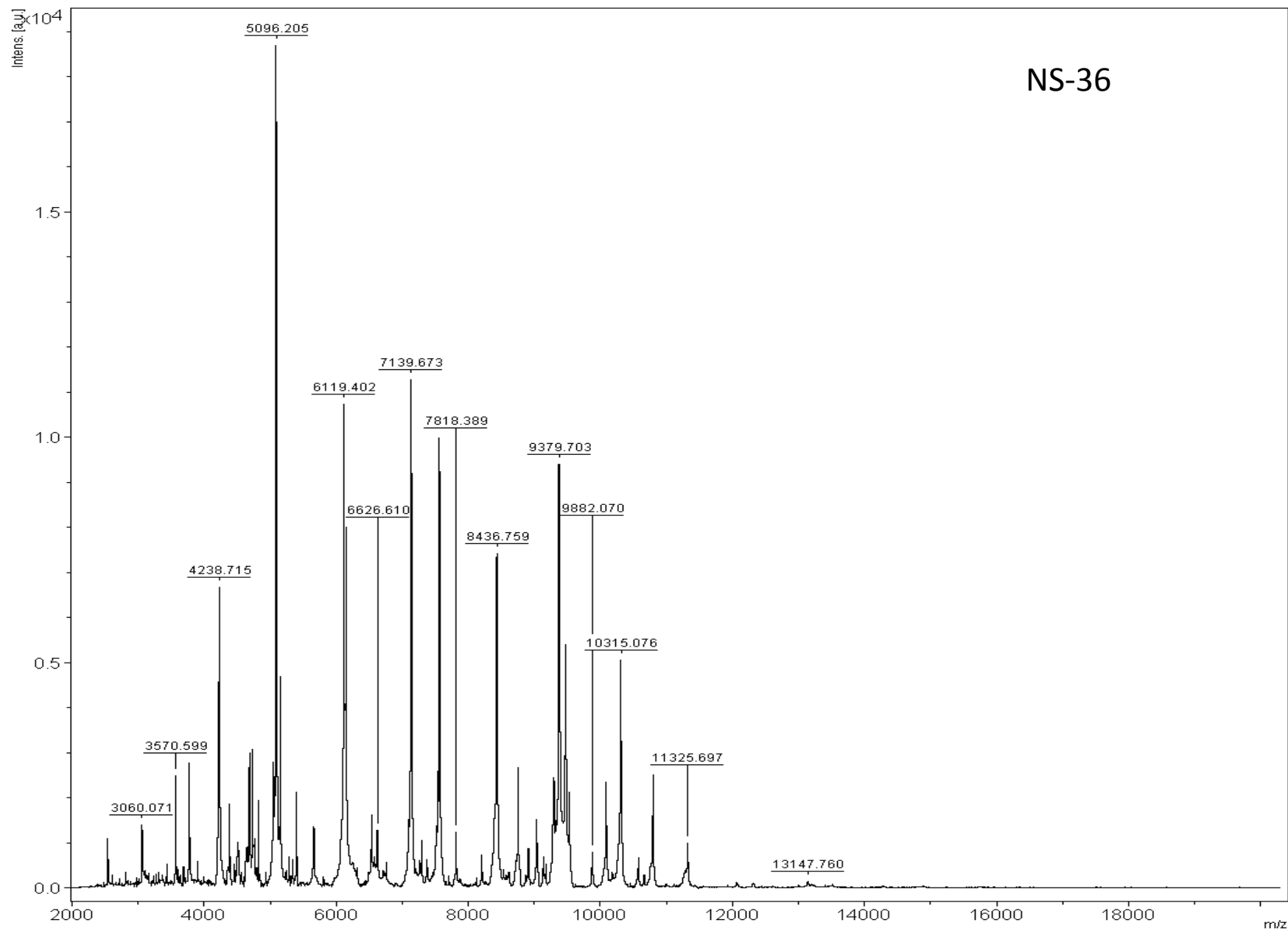

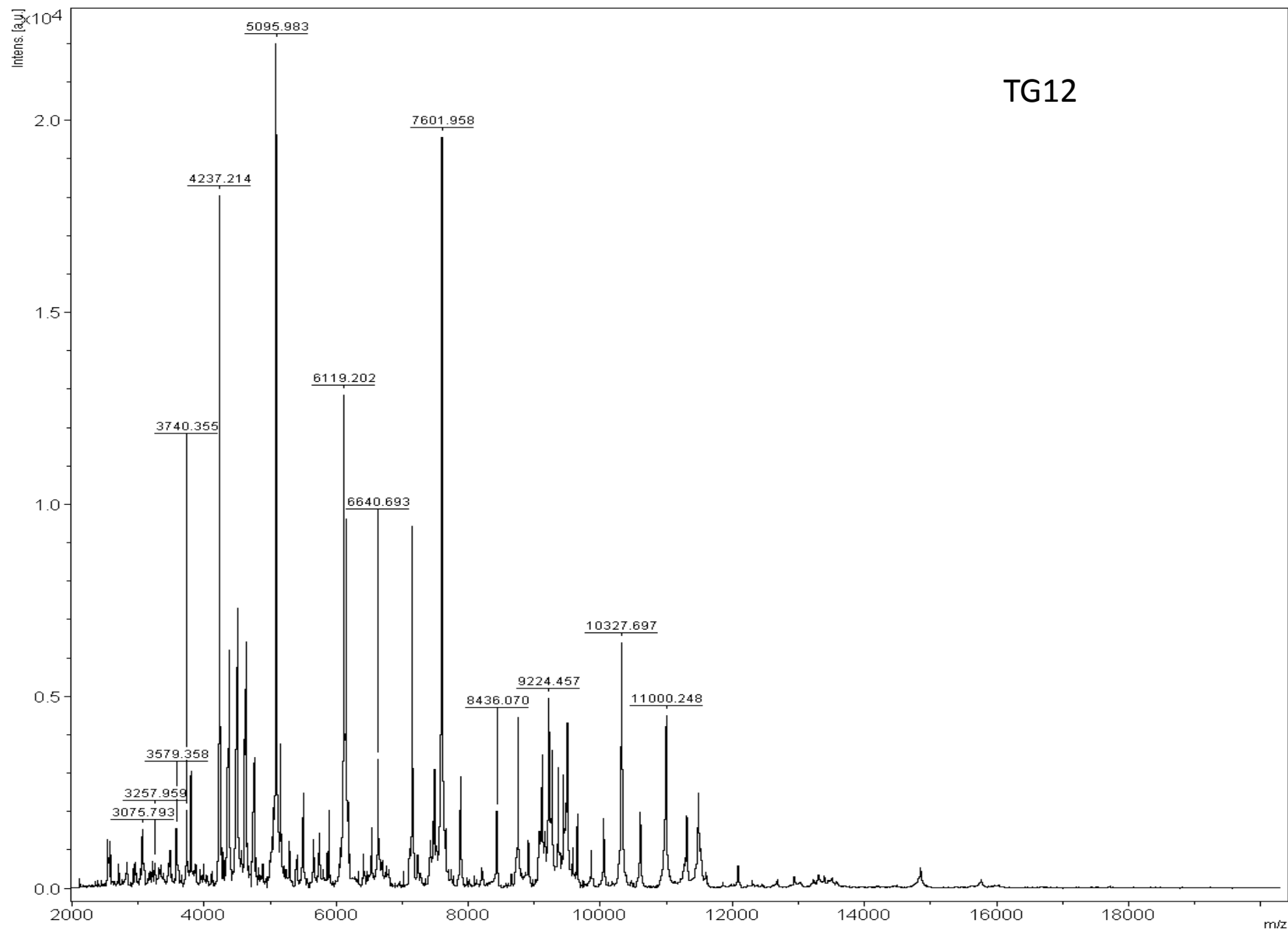

TG12

Supplement: Supplementary Figure 1 — Mass spectra of Pseudoalteromonas type strains. The strains are in alphabetical order as listed in the Materials and Methods Section. [file Image1.PDF]

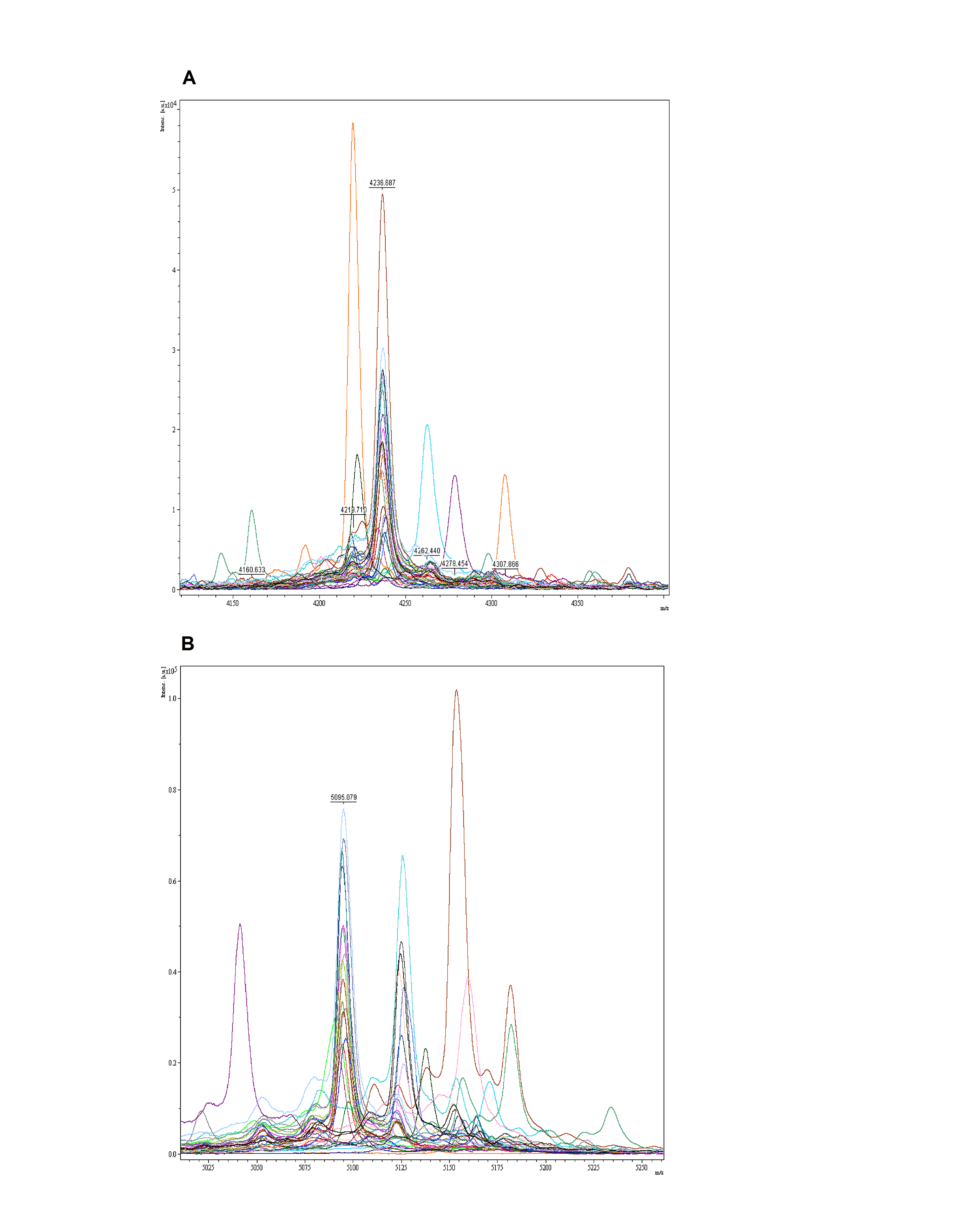

Supplement: Supplementary Figure 2 — Overlaid mass spectra of Pseudoalteromonas type strains where the most common peaks were observed. Nearly all the isolates have a peak at m/z 4236 (A) and most of them have a peak at m/z 5095 (B). [file Image2.TIF]
